# γ-PGA Hydrolases of Phage Origin in *Bacillus subtilis* and Other Microbial Genomes

**DOI:** 10.1371/journal.pone.0130810

**Published:** 2015-07-09

**Authors:** Stefania Mamberti, Paola Prati, Paolo Cremaschi, Claudio Seppi, Carlo F. Morelli, Alessandro Galizzi, Massimo Fabbi, Cinzia Calvio

**Affiliations:** 1 Dept. of Biology and Biotechnology, Università degli Studi di Pavia, Pavia 27100 (I), Italy; 2 Istituto Zooprofilattico Sperimentale della Lombardia e dell'Emilia Romagna, Sezione Diagnostica di Pavia, Pavia 27100 (I), Italy; 3 Institute of Molecular Genetics, National Research Council of Italy, Pavia 27100 (I), Italy; 4 Department of Chemistry, Università degli Studi di Milano, Milan 20133 (I), Italy; 5 Italian Interuniversity Consortium on Materials, Science, and Technology (INSTM), Pavia Research Unit, Pavia 27100 (I), Italy; ContraFect Corporation, UNITED STATES

## Abstract

Poly-γ-glutamate (γ-PGA) is an industrially interesting polymer secreted mainly by members of the class *Bacilli* which forms a shield able to protect bacteria from phagocytosis and phages. Few enzymes are known to degrade γ-PGA; among them is a phage-encoded γ-PGA hydrolase, PghP. The supposed role of PghP in phages is to ensure access to the surface of bacterial cells by dismantling the γ-PGA barrier. We identified four unannotated *B*. *subtilis* genes through similarity of their encoded products to PghP; in fact these genes reside in prophage elements of *B*. *subtilis* genome. The recombinant products of two of them demonstrate efficient polymer degradation, confirming that sequence similarity reflects functional homology. Genes encoding similar γ-PGA hydrolases were identified in phages specific for the order Bacillales and in numerous microbial genomes, not only belonging to that order. The distribution of the γ-PGA biosynthesis operon was also investigated with a bioinformatics approach; it was found that the list of organisms endowed with γ-PGA biosynthetic functions is larger than expected and includes several pathogenic species. Moreover in non-Bacillales bacteria the predicted γ-PGA hydrolase genes are preferentially found in species that do not have the genetic asset for polymer production. Our findings suggest that γ-PGA hydrolase genes might have spread across microbial genomes via horizontal exchanges rather than via phage infection. We hypothesize that, in natural habitats rich in γ-PGA supplied by producer organisms, the availability of hydrolases that release glutamate oligomers from γ-PGA might be a beneficial trait under positive selection.

## Introduction

Poly-γ-glutamic acid (γ-PGA) is a high molecular weight homo-polyamide, composed of glutamic acid monomers connected by amide linkages between the α-amino and γ-carboxylic groups, which is synthesized by several microorganisms mainly belonging to the *Bacilli* class [1]. The polymer is endowed with a number of favourable characteristics, being nontoxic, biodegradable, highly anionic, water soluble and rather stable [2]. For this reason it represents a promising biomaterial in a plurality of biotechnological applications, ranging from eco-friendly superabsorbent disposable diapers to scaffolds for tissue engineering or drug carriers [3]. Since chemical synthesis of such long polymers is currently unfeasible, quantitative and cost-effective γ-PGA purification from producer microbial strains is mandatory for its successful industrial exploitation [4]. Improvement of bacterial γ-PGA productivity has been addressed in a number of studies, many of which have been conducted in *Bacillus subtilis* strains. This bacterium is not only the model organism for gram-positive bacteria but also presents simple growth requirements, offers an extreme ease of genetic manipulation plus a variety of standardized reagents and techniques, and has a long established history as a safe γ-PGA producer; in fact the traditional Asian foods made of soybeans fermented by *Bacillus subtilis* species, known as *Natto* in Japan and *Cheonggukjang* in Korea, show a typical slimy texture due to the high content of γ-PGA [5].

In *B*. *subtilis* γ-PGA synthesis is carried out by a trans-membrane protein complex, namely the γ-PGA synthase, which is composed of 4 polypeptides (PgsB, PgsC, PgsAA, PgsE) encoded by the *pgs* operon; the synthase secretes γ-PGA during polymerization, allowing free polymer accumulation in the culture media [6, 7]. γ-PGA isolated from *B*. *subtilis* is a heterochiral polymer composed of D- and L-glutamic acid isomers (γ-DL-PGA) in which D-Glu represents between 50 and 80% of the total glutamic acid content [1, 8]. It has been suggested that release of polymer chains from the synthase complex is mediated by the γ-PGA-endo-hydrolase PgdS, whose gene is immediately downstream of the *pgs* operon [9–11].

In *Bacillus anthracis*, another gram-positive bacterium, γ-PGA has a 100% D-Glu composition (γ-D-PGA) [8] and is covalently anchored to the peptidoglycan layer; there, the non-immunogenic capsule protects the bacterium from the host’s immune response [12, 13]. Hence in this organism the homologous synthase components are called CapB, CapC, CapA and CapE, from capsule. *B*. *anthracis cap* genes are not chromosomally encoded but are localized on the virulence plasmid pXO2 [14–16]. On such plasmid the *cap* operon contains an additional gene absent in *B*. *subtilis*, *capD*, that codes for an *N*-terminal nucleophile hydrolase of the γ-glutamyltranspeptidase (GGT) family, responsible for connecting γ-PGA chains to the cell wall [17, 18].

Due to the particular linkage between glutamic acid residues γ-PGA is resistant to common proteases and can only be hydrolyzed by specific γ-PGA-degrading enzymes [2, 19]. In *B*. *subtilis* only two γ-PGA degrading enzymes are currently known: PgdS, which reduces polymer size through endo-hydrolysis [10, 11, 20], and GGT, whose gene is unlinked to the *pgs* operon, which removes single subunits from the *N*-terminal side of the polymer [21]. Both PgdS and GGT are secreted enzymes and therefore have free access to the accumulating polymer [21–23]. Indeed, removal of the genes coding for PgdS and GGT in a γ-PGA producer strain doubles polymer recovery, thus increasing overall bacterial productivity [24]. In *B*. *anthracis*, overexpression of the GGT-family member CapD leads to capsular polymer degradation reducing bacterial virulence [25–28].

γ-PGA-degrading enzymes represent a serious industrial problem in modern *Natto*/*Cheonggukjang* production plants, since they compromise the typical viscous texture associated with the extremely long polymer chains. In those settings, degradation of high molecular weight γ-PGA is occasionally observed and related to phage infections [29]. In 2003 Kimura and collaborators isolated phage ΦNIT1 from spoiled *Natto* and used it to infect *B*. *subtilis natto*; from the supernatant of the infected cultures they laboriously purified a new type of γ-PGA depolymerase that was named PghP (γ-PGA hydrolase from phage) [30]. PghP is a 23-kDa enzyme able to degrade high molecular weight γ-PGA. Structurally it is a metallopeptidase related to carboxypeptidase A and in fact it requires Zn^2+^ in order to exert its hydrolytic activity [31, 32].

Several hypotheses have been advanced to explain the biological function of γ-PGA in bacteria, including a protective effect against dehydration and heavy metal toxicity, respectively attributed to the high hydrophilic capacity and the strong anionic charge of the polymer; indeed, those favorable characteristics are also at the basis of the biotechnological attractiveness of γ-PGA [33]. In pathogenic species such as *B*. *anthracis*, the main role of γ-PGA is probably to enhance virulence by shielding the bacterium from the host’s immune surveillance. In fact, deletion of *cap* genes drastically attenuates virulence in mouse models of *B*. *anthracis* infection [34]. Yet, the existence of phage-encoded γ-PGA-specific degradative enzymes supports the hypothesis that the polymer might also provide an anti-phage shield. Indeed, Kimura and coworkers showed that ΦNIT1 could successfully replicate in *B*. *subtilis* subsp. *natto* throughout the growth cycle while BS5, a non PghP-carrying phage, was virulent only during the exponential phase, in which γ-PGA is not produced, and was not able to infect the strain during γ-PGA-production. BS5 gained infectivity in stationary phase if supplemented with purified PghP [30]. These results strongly suggest that γ-PGA constitutes an efficient physical barrier against phage attacks.

In this work four different *pghP* homologue genes, coding for products currently annotated as phage-related proteins of unknown function, have been identified in prophage or putative prophage regions of the *B*. *subtilis* genome. Two of these *pghP*-like genes have been cloned and expressed in *E*. *coli* and their γ-PGA hydrolytic activity has been confirmed by degradation of *B*. *subtilis* γ-PGA.

Homologous *pghP-*like genes were identified in a large number of sequenced microbial genomes, and it was found that their presence is not a distinctive feature of organisms synthesizing γ-PGA. The analysis of the data suggests that genes coding for γ-PGA-degrading enzymes might be under positive selection once integrated in microbial genomes.

Moreover, genes encoding the γ-PGA biosynthetic machinery have been recognized in more than 300 microbial species, largely expanding the current list of potential γ-PGA producer strains.

## Materials and Methods

### Bacterial strains and growth conditions

*Bacillus subtilis* strain 168 (PB1831 [35] and E*scherichia coli* strain DH5α (*supE44 lacU169* [Δ80*lacZ*ΔM15] *hsdR17*[r_K_^−^m_K_^+^] *recA1 endA1 gyrA96 thi-1 relA1*), used for molecular cloning, and BL21(DE3) (F^−^ *ompT hsdS*_B_[r_B_^−^ m_B_^−^] *gal dcm lon* λ[DE3]), used for protein purification, were grown at 37°C in LB broth (tryptone, 10 g; yeast extract, 5 g; NaCl, 10 g per liter). Media were routinely solidified with 1.5% agar. When required, media were supplemented with ampicillin (100 μg/ml). When appropriate IPTG (isopropyl-β-D-thiogalactopyranoside) was added to the media.

### Cloning of *yndL* and *yoqZ*

The deduced *yndL* 636 bp ORF was amplified from *B*. *subtilis* 168 DNA with primers ShFyndL (5'-GATTTTTCAT**ATG**TTTACTCCGATTTCTTC-3', *Nde*I site underlined) and RyndL (5'-TCCGCTCGAGGTAGCTATTTTTGAGAAAACGGAAC-3', *Xho*I site underlined). Similarly, the deduced *yoqZ* 639 bp ORF was amplified from *B*. *subtilis* 168 DNA with primers ShFyoqZ (5'-GGAATTGCAT**ATG**CTAGCTGCAGATAAGTATAG-3', *Nde*I site underlined) and RyoqZ (5'-ATCCTCGAGTGGTGCTACACCAGCCATACAATAC-3', *Xho*I site underlined). In both cases, the forward primer inserted an ATG codon for a methionine (in bold) instead of a GGT or GTT codon, respectively, coding for valine. Each fragment was separately inserted between the *Nde*I and *Xho*I sites of pET-23b (to exploit the C-terminal His tag) to generate pETSL and pETSZ containing *yndL* and *yoqZ* respectively. The cloned ORFs were verified by DNA sequencing.

### YndL and YoqZ expression and purification

Expression of *yndL* and *yoqZ* was induced from pETSL and pETSZ in BL21(DE3) cells by using 0.2 and 1 mM IPTG, respectively. Cell were harvested 3 h after induction and pellets were resuspended in PG1 buffer (50 mM Tris-HCl pH 7.5, 200 mM KCl, 0.5 mM ZnCl_2_ and 15 mM Imidazole). After sonication cell lysates were brought to 0.1% Triton-X100 and 0.1% Tween-20 and kept on ice under gentle rocking for 10 min before centrifugation at 12,000 g for 20 min at 4°C. SDS-PAGE electrophoresis indicated that proteins remained in the insoluble pellet. The pellet was dissolved in 6 M guanidine hydrochloride and incubated at 4°C for 10 min. Insoluble material was removed by centrifugation at 12.000 g for 10 min at 4°C and the liquid fraction was incubated with Ni-agarose beads previously equilibrated in PG2 buffer (50 mM Tris-HCl pH 7.5, 200 mM KCl, 0.5 mM ZnCl_2_, 15 mM Imidazole, 0.1% Triton-X100 and 0.1% Tween-20) for 1 hour at 4°C. After extensive washing with buffer PG2 YndL and YoqZ proteins were eluted with 500 mM imidazole in PG2 buffer; eluted fractions were pooled and dialyzed against buffer C (50 mM Tris-HCl pH 7.5, 150 mM KCl, 5 mM ZnCl_2_ and 30% Glycerol). Proteins were judged to be more than 98% pure by gel electrophoresis. By Bradford assay YndL concentration was calculated 1.9 mg /ml and YoqZ 0.5 mg/ml.

### Isolation of γ-PGA

Crude *B*. *subtilis* γ-PGA was collected from culture supernatant of PB5383 strain by precipitation with 3 volumes of cold methanol as described [24]. The polymer was suspended in water, brought to pH 2.2 with HCl and precipitated again with 3 volumes of cold methanol. After resuspension in water the acid polymer was extensively dialyzed against water and subsequently brought to pH 8 with concentrated KOH. γ-PGA from *B*. *anthracis* was isolated essentially as described [36] with minimal modifications. In detail, *Bacillus anthracis* strain IZSLER- PV1989 (corresponding to isolate n. 183 described in [37] was grown on nutrient agar containing 0.7% NaHCO_3_ and incubated at 37°C for 24 hours with 20% CO_2_ [38]. Bacteria were suspended in water, autoclaved for 45 min at 121°C and centrifuged at 10,000 g for 10 min. The supernatant was treated with DNase I and RNase A in nuclease buffer (0.1 M Tris-HCl pH 8.0, 10 mM MgCl_2_, 0.1 M NaCl, 0.02% (w/v) NaN_3_) for 1 h at 37°C. At this stage the solution was divided in two aliquots: trichloroacetic acid was added to one of the aliquots to a final 5% (w/v) concentration, kept on ice for 30 min and centrifuged 30 min at 12,000 g at 4°C. The supernatant was neutralized to pH 7 with 5 M NaOH, dialyzed against water before vacuum drying and then suspended in water. The second aliquot of *B*. *anthracis* γ-PGA was directly precipitated with 3 volumes of methanol and the pellet was suspended in water after vacuum drying. Aliquots of *B*. *anthracis* polymer deriving from the two procedures were visually compared by agarose gel electrophoresis before and after enzymatic digestion. No difference in polymer behavior could be detected between the two purification protocols (data not shown).

### Enzymatic digestion of γ-PGA

Enzymatic hydrolysis assays were routinely carried out in 7 μl-reactions containing: 2.5 μl γ-PGA (approximately 12 μg), 100 mM Tris-HCl pH 8.5 and different amounts of YndL or YoqZ as specified in each fig legend. Reactions were incubated at 37°C and stopped by inactivation at 95°C for 3 min after addition of 1 μl of γ-PGA loading dye (5 mg/mL Bromophenol Blue, 50% (v/v) Glycerol in TAE buffer). Electrophoretic separation was carried out in 1.5% agarose gels in TAE buffer. γ-PGA was visualized by staining with 0.5% methylene blue in 3% acetic acid for 30 min and destaining in H_2_O. Each single enzyme was assayed against γ-PGA more than 30 times and representative results are shown in Figs 3 and 5.

For the experiment *in vivo* shown in Fig 4, encapsulated *B*. *anthracis* cells were smeared on a microscope slide and treated with 0.5 μg of YoqZ in 50 μL of buffer (50 mM Tris-HCl pH 7.5, 200 mM KCl, 0.5 mM ZnCl_2_) for 10 minutes at 37°C. Non-capsulated cells were obtained by growing spores in nutrient agar in the absence of NaHCO_3_. Cells were stained with polychrome methylene blue [38] and visualized using an Eclipse E200 microscope (Nikon) with a 100× oil immersion objective; images were recorded with a CCD digital camera (CCD 560 Nikon). Due to intrinsic risk associated with manipulation of a virulent *B*. *anthracis* strain, the experiment shown in Fig 4 was performed only once.

### Bioinformatics Analysis

FASTA files for PghP, PgsB and PgsC were retrieved from the Universal Protein Resource Knowledgebase (http://www.uniprot.org/) and carried the following IDs: [UniProt: Q852V1] for PghP (Q852V1_9CAUD); [UniProt: P96736] for the product of *pgsB* (CAPB_BACSU, PGA synthase CapB); [UniProt: P96737] for the product of *pgsC* (CAPC_BACSU, PGA synthase CapC); except for PghP, all sequences belong to *Bacillus subtilis* subsp. *subtilis*, strain 168. PghP protein sequence was used to screen *B*. *subtilis* subsp. *subtilis* str. 168 genome (taxID:224308) with BLASTp algorithm with default parameters [39]. Four hits were retrieved, namely BSU20460, BSU12480, BSU17270 and BSU17820. The corresponding FASTA files were downloaded from Uniprot and carried the following IDs [UniProt: O31913] for the product of *yoqZ* (SPBc2 prophage-derived protein); [UniProt: O34785] for the product of *yjqB* (PBSX phage-related replication protein); [UniProt: O31789] for the product of *ymaC* (phage-related replication protein); [UniProt: O31815] for the product of *yndL* (phage-related replication protein). Sequence alignment was performed with the Clustal Omega web tool (http://www.ebi.ac.uk/Tools/msa/clustalo/) using default parameters [40]. The comparative analysis of PghP with YjqB, YmaC, YoqZ and YndL was carried out with the Emboss Needle web tool (http://www.ebi.ac.uk/Tools/psa/emboss_needle/) using default parameters.

PghP, PgsB and PgsC homology search was performed (on January 28^th^, 2015) on the “non-redundant protein sequence (nr)” database with the Protein Blast web tool (http://blast.ncbi.nlm.nih.gov/Blast.cgi) using the BLASTp algorithm with default parameters with the exception of the “Expect threshold” parameter that was set to 0.001 to improve the significance of the results. All results were retrieved without any restriction on sequence similarity and identity. An R script (http://www.r-project.org/) was created to map each sequence obtained to the related Taxonomic Lineage using the NCBI Taxonomy database [41] available through the NCBI REST Web Service. All gaps in Taxonomy were marked as “unclassified” in order to create a balanced tree. All GIs lacking the species Taxonomy level were discarded.

All the results were grouped based on two different criteria: i) For data shown in the S1 Table all GIs were grouped at the species level. For each species we identified the presence of at least one hit from the BLASTp results (Query) and the number of hits associated to the species (Counts) including the GIs mapped to related subspecies. ii) For data shown in the S2 Table all GIs were grouped using their complete Taxonomy description, including the strain ID. For each Taxonomy ID we identified the presence of at least on hit from the BLASTp results (Query), the number of hits associated with each Taxonomy ID (Counts), and the range (min and max) in the number of amino acids from the Query and the hit (Sbjct) involved in the alignment, Alignment length, the percentage (min and max) of Identity and Similarity.

To map *pghP*-like genes with respect to predicted prophage regions, the GI of 55 randomly selected PghP-like ORFs, belonging to most genera of non-Bacillales species lacking *pgsB*, were used to retrieve the GI of the encoding nucleotide sequence (contig or entire genome) from the NCBI database and to map the *pghP*-like gene in its corresponding genomic sequence. Presence and map position of prophage regions were determined by analyzing each genome/contig with the Phage Search Tool (PHAST) web server [42] by using the genomic GI. Potential co-localization of *pghP*-like genes within prophage regions was manually examined. GI and map position of *pghP*-like genes, GI of genomes or contigs and their size, number of prophagic regions identified within, and co-localization results are given in the worksheet “Table of PghP and PgsB” contained in the S1 Table.

Moreover, a tBLASTn search was run with the PghP protein sequence [UniProt: Q852V1] and the Taxonomy BLAST Report (S1 File) was analyzed to determine the number of *pghP*-like paralogues per genome.

The Venn diagram has been obtained with the PNNL Venn Diagram Plotter software accessible at the OMICS.PNL.GOV website.

## Results and Discussion

### Identification of *pghP*-like genes in the *B*. *subtilis* genome

PghP is a very efficient -PGA endo-hydrolase, isolated and cloned from the *B*. *subtilis* phage ΦNIT1 by Kimura and Ito; through the coordination of a zinc ion it degrades very long -PGA polymeric chains producing small fragments composed of γ-glutamyl tri- tetra- and penta-peptides [32].

**Fig 1:**
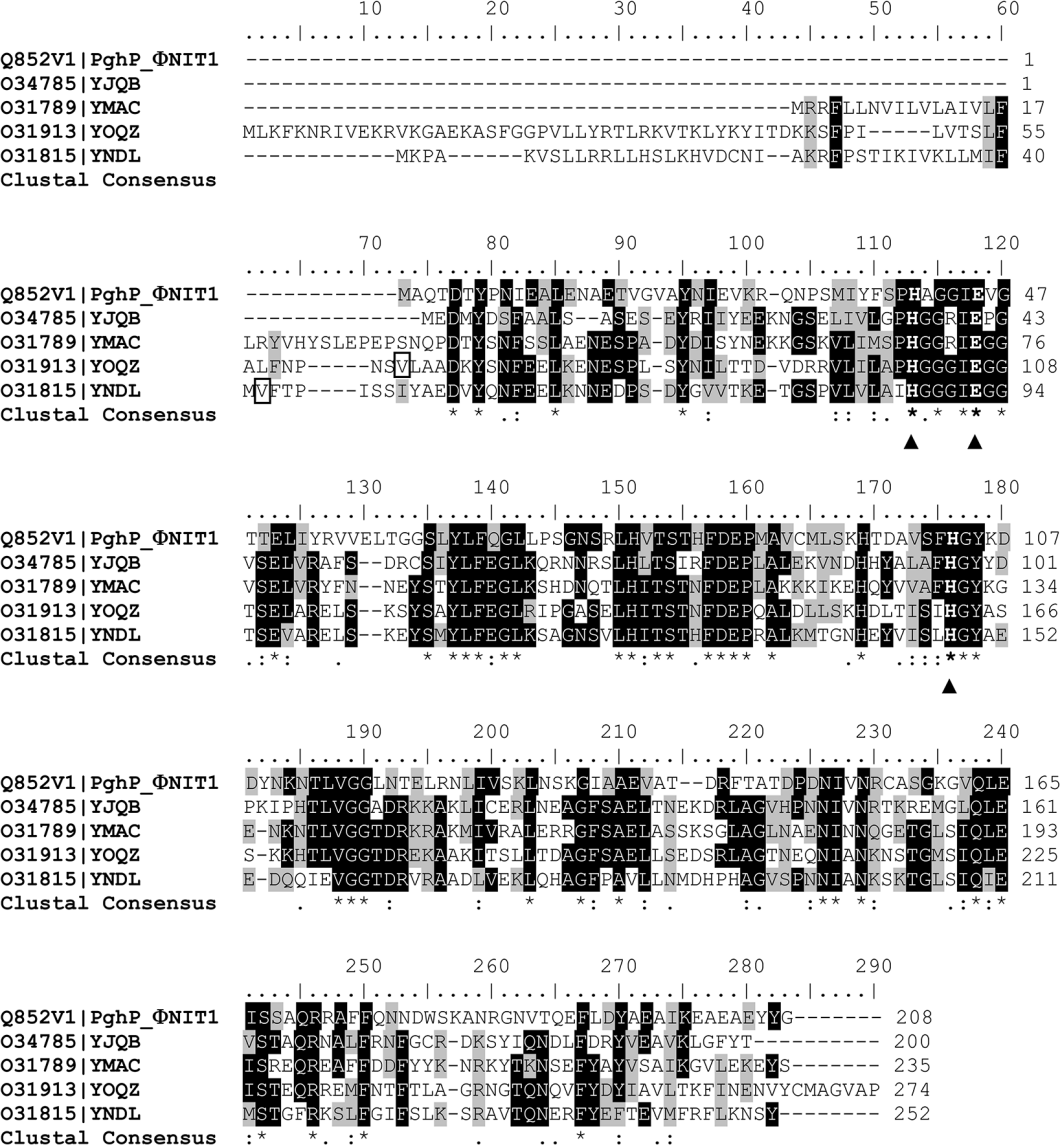
Clustal alignment of *B*. *subtilis* gene products showing similarity to *Bacillus* phage ΦNIT1 PghP. Black triangles below the sequences point to residues involved in Zn coordination according to the PghP structure [31, 32]. The valine residues enclosed in a rectangle in YoqZ and YndL sequences were transformed in the initial methionine in the recombinant proteins. An * (asterisk) in the clustal consensus line indicates positions which have a single, fully conserved residue. A: (colon) indicates conservation between groups of amino acids with strongly similar properties. A. (period) indicates conservation between groups of amino acids with weakly similar properties.

In an extension of our efforts to improve -PGA productivity in *B*. *subtilis* [24] a BLAST search for PghP homologue enzymes was performed on *B*. *subtilis* strain 168 genome ORFs using the phage protein sequence as a query. Four different hits were identified which correspond to the predicted products of *yjqB*, *ymaC*, *yoqZ* and *yndL* [43]. Sequence alignment confirmed that the four gene products are highly similar amongst themselves and display outstanding similarities to PghP, ranging from 53.8% to 41% (Fig 1 and Table 1) [44]. The four genes are encased in prophages or putative prophage sequence relics present in the *B*. *subtilis* chromosome: *yjqB* is located in the PBSX prophage element at 1,390 kb; *yoqZ* is in the SPβ prophage region at 2,190 kb [44], *ymaC* and *yndL* are in the prophage-like element 5 (numbered in order of appearance according to Kunst et al. [43]) at 1,864 and 1,915 kb, respectively. In all the databases the four genes are currently annotated as coding for phage-related (replication) proteins, although no functional studies for any of them have been conducted to support such a prediction. The encoded proteins share amongst themselves and with phagic PghP the signature of a sequence-based domain of unknown function, namely DUF867, equivalent to the InterPro family IPR008585 or to Pfam PF05908 (S2 File) [45–47]. The DUF867 protein family lacks a functional annotation although it is sometimes connected with the crystal structure of the characterized protein PghP from ΦNIT1 [PDB: 3a9l] [32].

**Table 1:** Comparison of *B*. *subtilis* paralogues with PghP. Similarity and identity values were obtained by comparative analysis of *B*. *subtilis* PghP-like proteins with PghP as specified in Methods.

Gene product	Similarity	Identity
	*versus* PghP	
YjqB	53.8%	36.8%
YmaC	47.2%	33.6%
YoqZ	41.6%	30.6%
YndL	41.0%	27.2%

### Functional validation of two *B*. *subtilis* PhgP-like proteins

**Fig 2:**
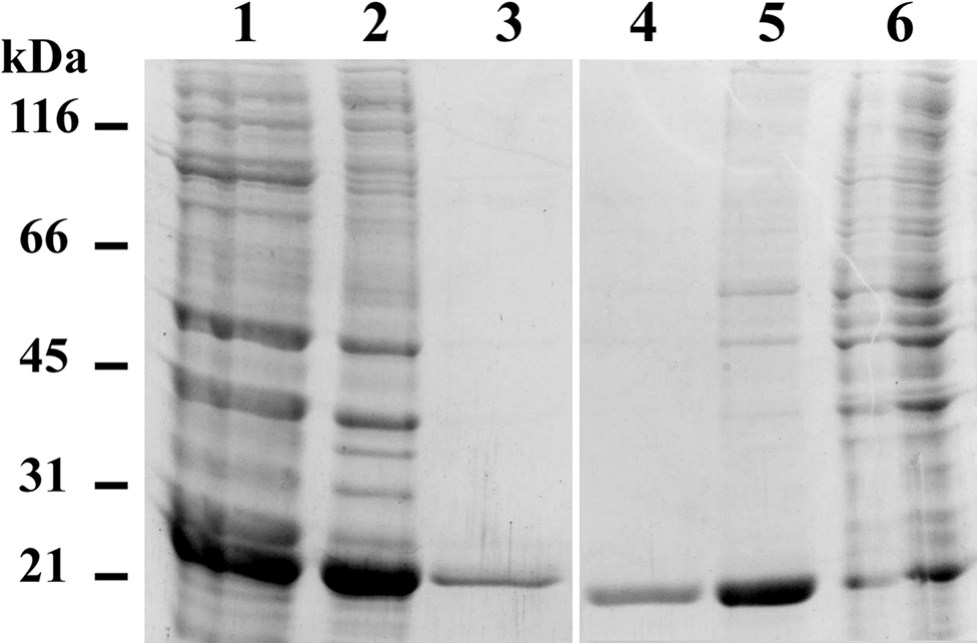
Purification of YndL and YoqZ. His-tagged YndL and YoqZ were purified from the insoluble *E*. *coli* lysate fraction using Ni-NTA agarose beads under denaturing conditions as described in Material and Methods. Lanes 1 and 6: 12 μL flow-through fractions; lanes 2 and 5: 1 μL beads; lanes 3 and 4: 6 μL eluted proteins. Lanes 1–3 refer to YndL purification; lanes 4–6 refer to YoqZ purification. Molecular weight markers are indicated on the left.

The high similarity observed between the four bacterial ORFs and phage PghP is not *per se* sufficient to classify them as γ-PGA hydrolases. To validate their annotation functional assays were performed. To this end the two ORFs with lower similarity to PghP, *yoqZ* and *yndL*, were cloned and expressed in *E*. *coli*. All attempts to express the *yndL*-encoded protein from the first methionine predicted in the 1997 *B*. *subtilis* genome annotation [43] were unsuccessful. To overcome this problem, possibly related to the inaccuracy of automatic annotation systems, PCR primers were designed for both genes that allowed positioning of the initial ATG in proximity of the PghP initiator codon (Fig 1). The two PCR products were cloned in frame with a C-terminal histidine-tag that was exploited for protein purification. Despite several attempts with different protocols induction of expression invariably led to accumulation of the recombinant proteins in the insoluble fraction. Cell pellets were thus denatured before Ni-affinity purification. Purified proteins were soluble and stable upon storage at -20°C for several months (Fig 2).

**Fig 3:**
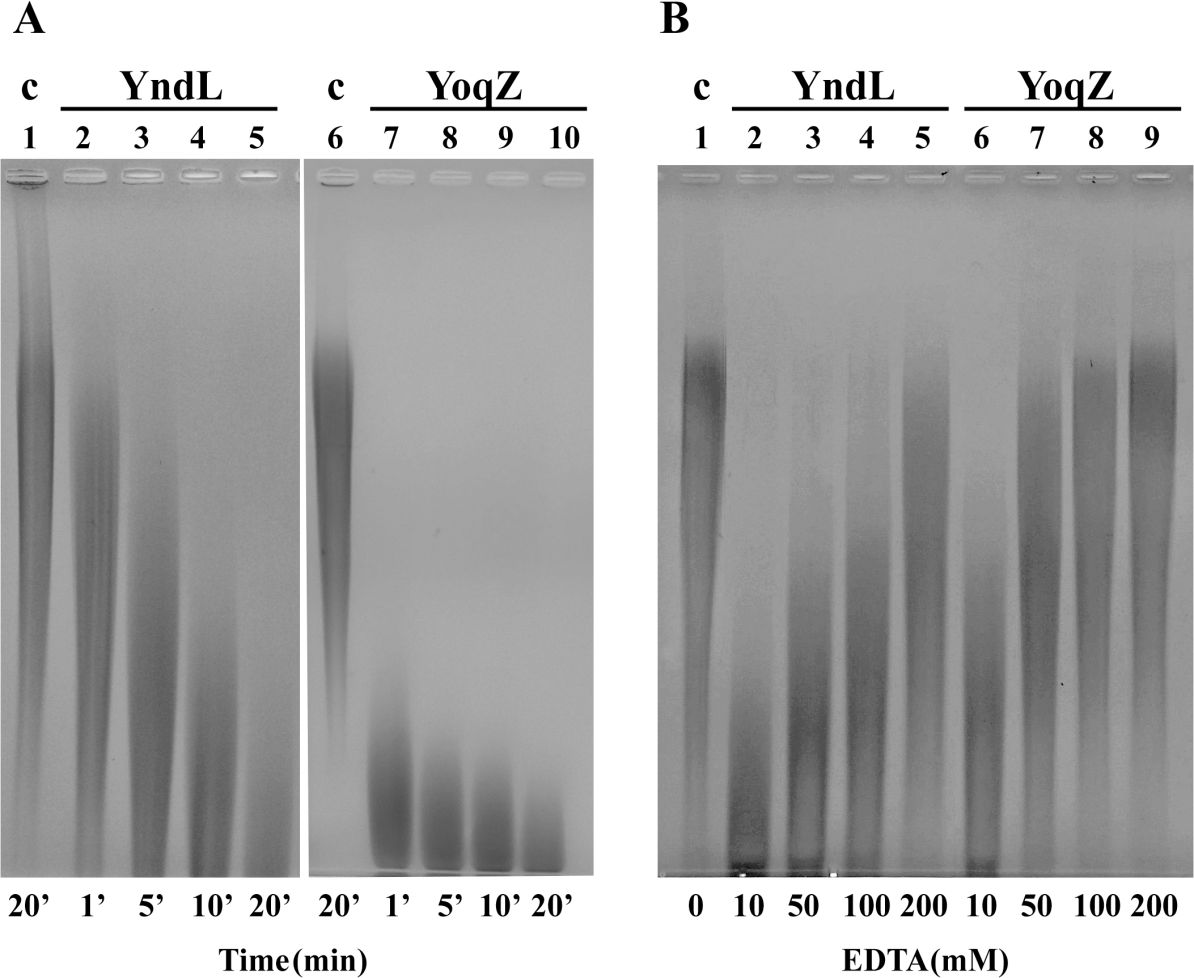
Degradation of *B*. *subtilis* γ-DL-PGA by YndL and YoqZ. A. *B*. *subtilis* γ-DL-PGA incubated with 0.045 μg YndL (lanes 2–5) or 0.009 μg YoqZ (lanes 7–10) at 37°C before separation on an agarose gel. Reactions were stopped after 1’ (lanes 2 and 7), 5’ (lanes 3 and 8), 10’ (lanes 4 and 9) and 20’ (lanes 5 and 10) by heating at 95°C for 3 min. Control reactions in lanes 1 and 6 were incubated for 20’ in the same conditions without enzyme. **B**. *B*. *subtilis* γ-DL-PGA was incubated at 37°C for 60’ with 0.5 μg YndL (lanes 2–5) or 0.5 μg YoqZ (lanes 6–9) with the addition of 10 mM (lanes 2 and 6), 50 mM (lanes 3 and 7), 100 mM (lanes 4 and 8) and 200 mM (lanes 5 and 9) EDTA. No enzyme was added in the control reaction in lane 1.

Functional assays were performed by incubating *B*. *subtilis* γ-DL-PGA with YndL or YoqZ for increasing amounts of time and visually verifying their effect on polymer size by gel separation. As observed in Fig 3A the molecular weight of the polymer was progressively reduced over time by the activity of both enzymes. The inhibitory effect of EDTA (Fig 3B) confirmed a metal-dependence for the activity of YndL and YoqZ [32]. The extremely high concentration of EDTA required for full inhibition is most likely due both to the abundance of cations carried over by γ-PGA during recovery from the culture medium and to the presence of excess zinc in the enzymes’ preparations.

### Action of bacterial PghP-like proteins against *B*. *anthracis* γ-PGA

**Fig 4:**
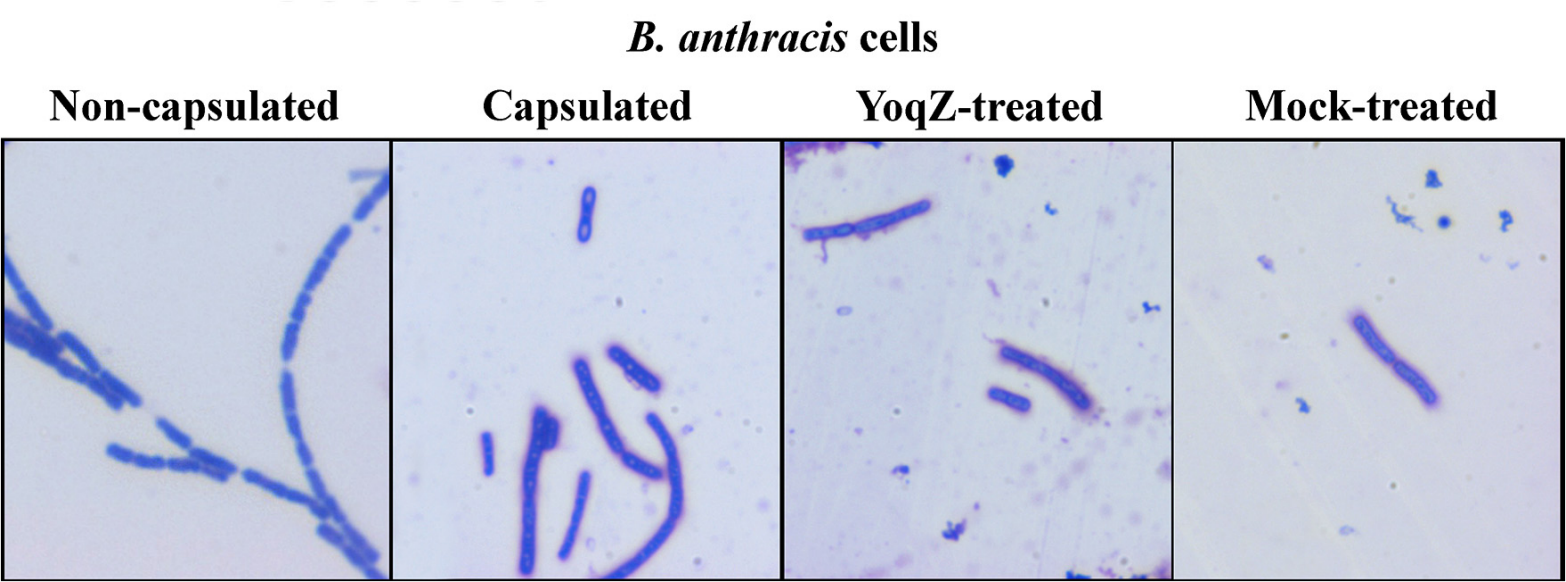
Resistance to enzymatic treatment of the *B*. *anthracis* capsule. *B*. *anthracis* cells were grown under conditions that allowed (B) or did not allow (A) capsule production. Encapsulated cells were either incubated for 10 min at 37°C with YoqZ in digestion buffer (C) or with buffer alone (D) before staining (original magnification 100x).

**Fig 5:**
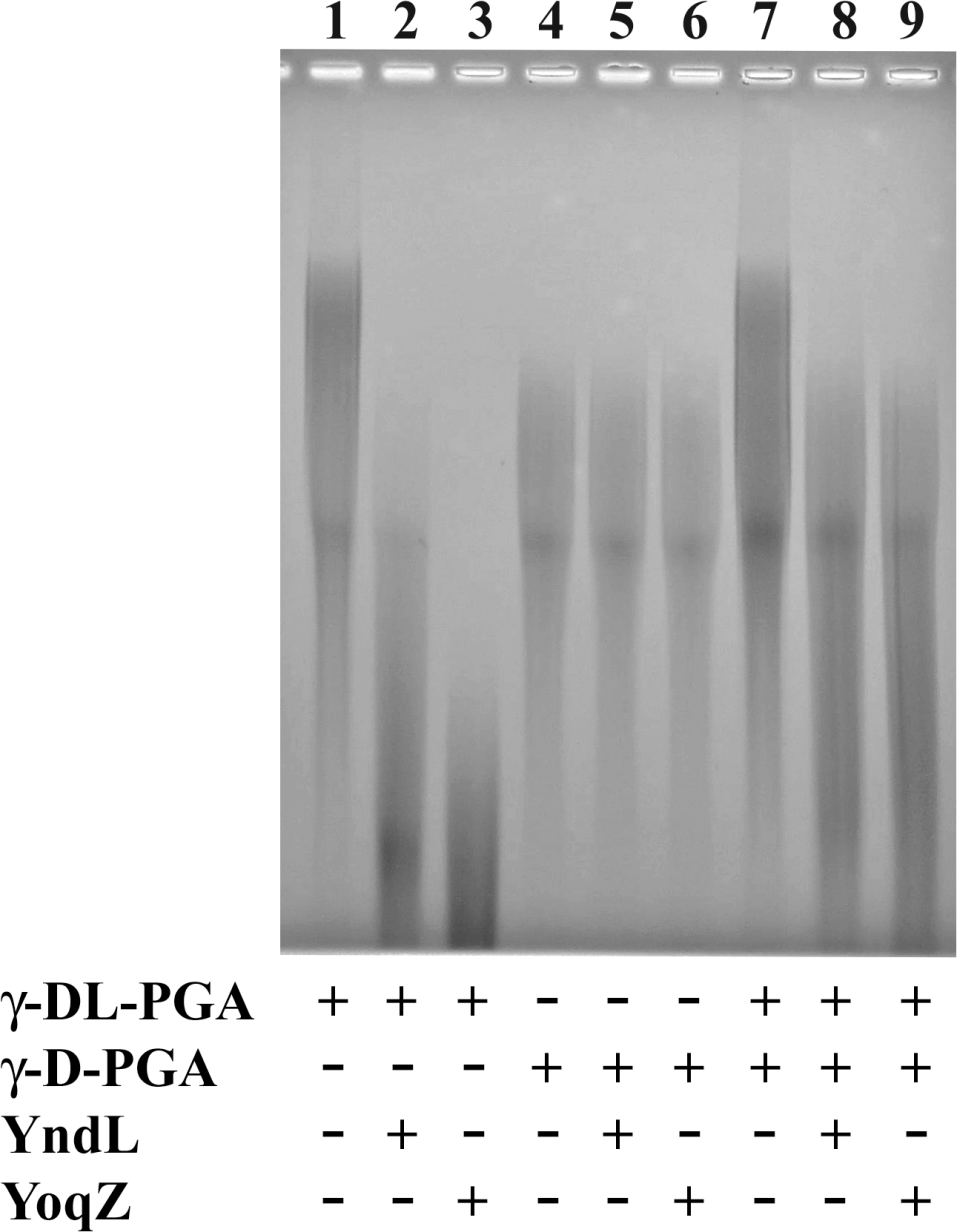
*B. anthracis* γ-D-PGA is not a substrate for YndL and YoqZ. *B*. *subtilis* γ-DL-PGA (2 μL in lanes 1–3), *B*. *anthracis* γ-D-PGA (2.5 μL lanes 4–6) or a mixture of both (2+2.5 μL in lanes 7–9) were incubated at 37°C for 60’ in the absence (lanes 1, 4, 7) or in the presence of 0.5 μg YndL (lanes 2, 5, 8) or 0.5 μg YoqZ (lanes 3, 6, 9). Enzymatic activity was stopped by heating at 95°C for 3 min before gel separation.

As the γ-PGA capsule constitutes a major virulence determinant in *B*. *anthracis* infections the availability of enzymes able to efficiently degrade it could facilitate development of new drugs to treat the disease [27, 28]. Indeed, phagic PghP was reported to degrade the capsule of live *B*. *anthracis* cells, although the same recombinant protein was ineffective against purified *B*. *anthracis* γ-D-PGA and had no consequence on macrophage phagocytosis and neutrophyl killing [27]. To verify the substrate specificity of the bacterially encoded PghP-like enzymes, resistance of *B*. *anthracis* γ-D-PGA to degradation was tested both *in vivo* and *in vitro*. Encapsulated *B*. *anthracis* cells were incubated with purified YoqZ and then visualized by methylene blue staining. No difference could be detected in the integrity of *B*. *anthracis* γ-D-PGA in terms of capsule visibility between the treated and untreated samples, in contrast to what reported for PghP [27] (Fig 4). However, in agreement with other authors [27] and with the results obtained *in vivo*, resistance of γ-D-PGA to enzymatic treatment was observed in *in vitro* degradation assays of the *B*. *anthracis* polymer in parallel with *B*. *subtilis* γ-DL-PGA. As shown in lanes 5 and 6 in Fig 5, the molecular weight of γ-D-PGA was not modified by incubation with either enzyme, in contrast to the degradation of *B*. *subtilis* γ-DL-PGA. One possible cause for this negative result is the existence of an inhibitor molecule co-purified with *B*. *anthracis* γ-D-PGA that is able to prevent enzymatic degradation of the polymer. If this is the case, the presence of γ-D-PGA should also compromise γ-DL-PGA degradation. To test this hypothesis we took advantage of the distinct migration profile of the two polymers upon electrophoresis, which is related to their respective average molecular weight: γ-DL-PGA from *B*. *subtilis* PB5383 is characterized by an extremely high average molecular weight (>1.5 MDa, as previously determined [24]) and migrates mainly in the upper part of gels whereas, in the same conditions, *B*. *anthracis* γ-D-PGA migrates faster, localizing halfway through the gel. Hence, γ-PGA derived from both microorganisms was mixed before enzyme treatment and then separated on a gel. As shown in lanes 8 and 9 in Fig 5, the high molecular mass fraction of the polymer derived from *B*. *subtilis* could still be degraded by the PghP-like enzymes, while *B*. *anthracis* γ-D-PGA remained intact upon treatment. This result, in line with previous findings [27], ruled out the hypothesis that an inhibitory activity is the basis of γ-D-PGA’s resistance to degradation. Overall, the data show that the chromosomally encoded *pghP* homologues are indeed γ-PGA hydrolases that display a marked substrate specificity equivalent to the phagic PghP enzyme.

In light of the results obtained, we suggest renaming the *B*. *subtilis yjqB*, *ymaC*, *yndL* and *yoqZ* genes as *pghB*, *pghC*, *pghL* and *pghZ* respectively (phage-derived gamma-PGA hydrolase). To impose an appropriate name upon an ORF is not just an academic exercise, rather it is a central task in better understanding the genetic make-up of a bacterium and its ecological and evolutionary history.

Moreover, since the signature domain DUF867 (alias PF05908 or IPR008585) is 189 amino acids-long and spans the entire PghP, PghB, PghC, PghL and PghZ length (http://pfam.xfam.org/family/PF05908; S2 File), we suggest removing DUF867 from the list of domains of unknown function and assigning the caption “gamma_PGA_hydrolase” signature domain to PF05908 and IPR00858, thus contributing to the improvement of the annotation process of a large number of microbial genomes.

### Distribution of *pghP*-like genes in microbial genomes

In order to determine whether PghP-hydrolases are present in other bacteria a BLAST search was carried out using ΦNIT1PghP as a query. For each hit taxonomic data were retrieved at the level of species. Sequences with high similarity to PghP were found in 261 organisms (as of January 2015). Most of the entries (236) are found in bacteria, particularly in *Mycobacterium tuberculosis*, but PghP-like proteins are also present in archaea (5), eukaryota (3) and, of course, in 17 phages (S1 Table). The phagic gene products are found exclusively in phages specific for *Bacillus* and *Staphylococcus* species (14 and 3 hits, respectively). Such specificity is reflected by the high incidence of *pghP*-like genes in *Bacilli* and *Staphylococci* in which the genes are likely due to direct prophage insertions. Furthermore in these two genera multiple entries per organism are found, as in *B*. *subtilis*; the number of *pghP*-like paralogues per genome varies between 1 and 5 (S1 File) and variability in gene number can be observed among members of the same species. Other bacterial genera frequently carrying *pghP*-like genes in their genome are *Mycobacteria* and *Streptomycetes*, which usually limit the number of *pghP*-like genes to 1 copy per genome. PghP-like ORFs are also found in 53 other bacteria genera (a detailed list of the taxonomic distribution of *pghP*-like genes is given in the pivot tables in the S1 Table). Most of the γ-PGA-hydrolase genes appeared to occur in genera for which γ-PGA production had not been reported and, in fact, *pghP*-containing phages that could have targeted those species were not identified in our search. Thus, the conserved γ-PGA-hydrolases found in those genera are unlikely to be due to phage infections.

### Distribution of γ-PGA biosynthetic genes in microbial genomes

Currently, reports of γ-PGA-producing organisms are limited to *Bacilli* and a few other bacterial, archaeal and eukaryotic species [1] although new γ-PGA-producing species are progressively being added to the list [48, 49]. To our knowledge no studies have specifically addressed the distribution of γ-PGA biosynthetic apparatus using a bioinformatics approach; it was thus necessary to assess whether γ-PGA synthesis was indeed confined to very few microorganisms in order to better understand the reason for the distribution of γ-PGA-hydrolases in so many microbial genomes.

A BLAST search was carried out for organisms containing sequences displaying a significant homology to *B*. *subtilis* PgsB and PgsC, the conserved components of the γ-PGA synthetic complex [49]. In this case complete taxonomic data were used, in order to easily identify the two genes in identical organisms (through their sequential GI numbers or strain-specific origin). Surprisingly, PgsB and PgsC homologues were identified in a larger number of organisms than expected. A substantial overlap between the PgsB and PgsC hits was observed, although the PgsB-based search allowed retrieval of a higher number of species than PgsC. This finding might be due to a lower conservation of the PgsC protein sequence or to its shorter length; in fact, PgsC was not found in archaeal species although they are known as polymer producers [50]. The combination of both PgsB- and PgsC-homologues was found in 250 bacterial species belonging to 58 genera (the complete dataset is provided in the S2 Table). Many of those species had not been reported as γ-PGA producers before. Thus, the list provided in the S2 Table is the first realistic estimate of organisms that, at least in some physiological conditions, can rely upon γ-PGA synthesis to protect themselves from environmental insults, although we are aware that being endowed with the coding capacity for γ-PGA biosynthesis does not necessarily imply the effective and constitutive expression of the genes [51].

### Correlation between production and hydrolysis of γ-PGA

**Fig 6:**
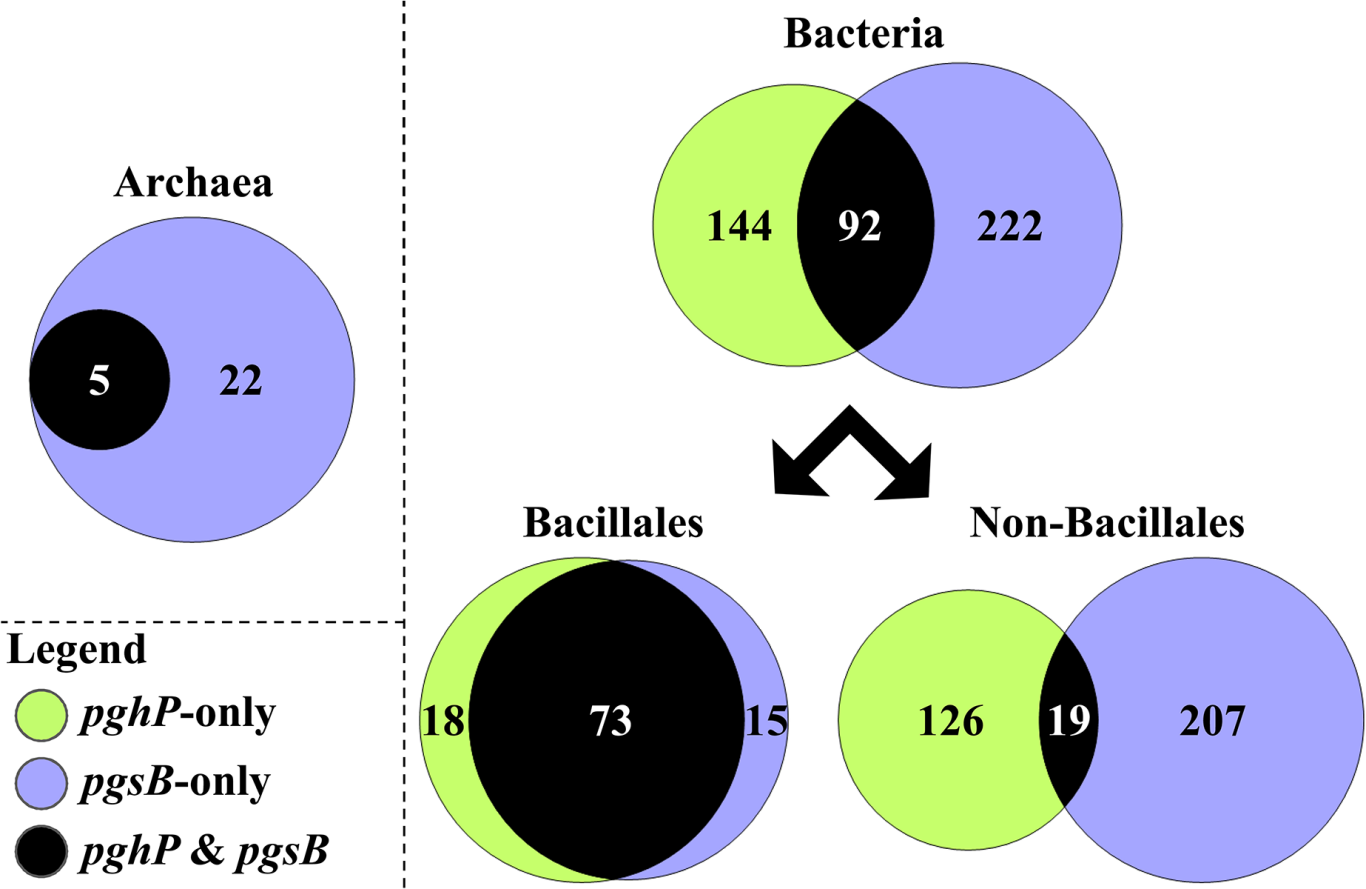
Relative distribution of *pghP* in bacteria and archaea genomes with respect to *pgsB*. In the Venn diagram green circles represent species that contain at least one *pghP*-like gene; violet circles represent species that contain *pgsB*. Numbers inside circles refer to the number of species containing either *pghP* or *pgsB*. Species that contain both genes are represented by the overlapping region in black (number inside). For each group (namely Archaea, total Bacteria, Bacillales, non-Bacillales) the size of circles and their overlapping region is drawn to scale. The raw data are available in the worksheet “Table of PghP and PgsB” contained in the S1 Table.

When the taxonomic distribution of PghP-like hydrolases and PgsB homologues were compared, at the level of species, it immediately struck our attention that there was a the peculiar correlation between γ-PGA-producing and PghP-containing organisms (Fig 6 and S1 Table). In fact, while for the archaea and the order Bacillales (in which *Bacilli* and *Staphylococci* are grouped) PghP is preferentially found in species containing PgsB (in 100% and 80% of the species, respectively), in non-Bacillales bacteria *pghP* is associated with the presence of the γ-PGA biosynthetic gene *pgsB* in only 13% of the cases. As already noted, the presence of *pghP* in *Bacilli* and *Staphylococci* phages justifies the strict association between the two genes in Bacillales: these organisms synthesize the polymer, i.e. have *pgsB*, and thus their phages carried—and settled in their genome—the hydrolases necessary for invasion. Conversely, in other bacterial orders, which include a large number (226) of putative γ-PGA-producers, *pghP* (145 hits) was found associated with *pgsB* in 19 species only. The presence of conserved *pghP*-like hydrolases in only five γ-PGA-producing archaea, for which no dedicated phage was identified in our PghP search, does not allow us to draw any significant conclusion.

Several speculations can be made for the inverse correlation between the two genes in non-Bacillales bacteria; the 87% of non-Bacillales species that do not produce γ-PGA but contain the γ-PGA hydrolases gene might be due to: i) loss of γ-PGA biosynthetic functions from those microbial genomes after prophage establishment; ii) a broader than expected host-range specificity for the *pghP*-containing *B*. *subtilis* phages, which thus carried their hydrolases along although they were not required for the actual target [52]; iii) the fact that *pghP*-like genes confer an evolutionary advantage during growth in natural habitats and thus spread among non-Bacillales species through horizontal gene transfer rather than through de-novo phage infections. In the former two cases *pghP*-like genes would be expected to localize within prophage regions. However, when presence and map location of prophage elements were predicted in 55 of those non-Bacillales species by using the sensitive Phage Search Tool (PHAST) web server [42], there was no evidence of co-localization (S1 Table). Therefore *pghP*-like genes found in the analyzed genomes are most consistent with a spread through horizontal gene transfer rather than phage infection. The relevance of γ-PGA hydrolases in microbial fitness might reside in the possibility of endowed organisms to feed on short glutamate oligomers released from γ-PGA secreted in the common environment by other producing species. According to this hypothesis, *pghP*-like genes could be considered phage functions that increase bacterial survival, similarly to “morons” [53].

In *B*. *subtilis* the hypothesis of an evolutionary advantage conferred by γ-PGA hydrolases is strengthened by the fact that *yjqB*, *yoqZ*, *ymaC* and *yndL* are expressed [54, 55] and that four different paralogues are maintained in the genome of several strains. Karamata and coworkers [44] have postulated that the maintenance of *B*. *subtilis* prophages is under positive selective pressure; the data shown in Fig 6 are in agreement with the proposition that γ-PGA hydrolase genes are partly involved in driving such positive selection.

## Conclusion

Four “*y*” genes present in the *B*. *subtilis* genome, buried in genetic elements of prophage origin, have been identified as coding for γ-PGA hydrolases. The existence of this type of enzyme in the genetic asset of four original *B*. *subtilis*-infecting phage strongly reinforces the notion that γ-PGA constitutes an effective anti-viral defense in bacteria.

Additionally, homologous *pghP*-like genes have been found in a large number of microbial genomes, which in many cases do not carry the γ-PGA biosynthetic genes. In the latter group, we could not find evidence of localization of such *pghP*-like genes in prophage elements. This finding can be interpreted with the hypothesis that the presence of this type of enzyme endows bacteria with the possibility to feed on glutamic acid released by free γ-PGA present in their environmental niche, and thus constitute a beneficial trait that justifies its spreading to such a large number of soil organisms, including two Fungi.

Moreover, a large number of potential γ-PGA producers were uncovered among bacteria and archaea by using a bioinformatics approach.

The work presented here also aimed at determining whether PghP-like γ-PGA hydrolases could be used for dismantling the γ-D-PGA capsule, a major virulence determinant of *B*. *anthracis*. The data collected, both *in vitro* and *in vivo*, demonstrate that, unlike γ-DL-PGA from *B*. *subtilis*, γ-D-PGA purified from *B*. *anthracis* is not a substrate for this class of enzymes. The reason for such dissimilar activity upon the two polymers remains to be firmly established; the simplest explanation is their differential enantiomeric composition, with the presence of L-Glu in *B*. *subtilis* γ-PGA [8]. *In vitro* studies on synthetic oligomers with defined stereochemical composition are currently underway to definitely elucidate this issue. These studies might help in characterizing the determinants of substrate specificity of PghP-like enzymes in order to design appropriate protein engineering approaches aimed at developing new therapeutic tools against *B*. *anthracis* infections by modulating their ability to accept γ-D-PGA as a substrate. Efforts are also being devoted to determine whether *B*. *subtilis pghP-*like genes have a detrimental effect on the stability of γ-PGA in producer strains.

## Supporting Information

S1 File Taxonomy BLAST reports for PghP. A pdf file of the Taxonomy report generated through the tBLASTn search with PghP (as described in Methods); the four hits from the *B. subtilis* subsp *subtilis* strain 168 paralogues are highlighted.
(PDF)

S2 File DUF867/PF05908 description page. Pfam web page reporting the description (and length) of the DUF867/ PF05908 domain, including the seed alignment used to generate the domain signature.
(PDF)

S1 Table Distribution of PghP-like /and PgsB-like proteins in the database. Excel file listing the hits (grouped at the level of species, as specified in Methods) identified through the PghP-based BLASTp search (PghP data) followed by the relevant pivot table; PghP hits are also presented together with those identified through the PgsB-based search (PghP and PgsB data) followed by a report table in which occurrence of the two hits is differentially highlighted. Data on the localization of a subset of *pghP*-like genes outside prophage elements are also given.
(XLSX)

S2 Table PgsB and PgsC distribution in microbial genomes. Excel file listing the hits identified through the PgsB- and PgsC-based BLASTp searches. Data are separated in three different worksheets according to superkingdom (bacteria, archaea and eukaryota); data for PgsB and PgsC homologues in bacteria are presented in pair, whereas species for which the two genes could not be assigned to the same strain are presented in a separate worksheet (unpaired bacteria). Pivot tables for bacteria and archaea are also given.
(XLSX)
