# Supplementary material for: γ-PGA Hydrolases of Phage Origin in Bacillus subtilis and Other Microbial Genomes
Source: PLoS One. 2015 Jul 9;10(7):e0130810. doi: 10.1371/journal.pone.0130810 (PMC4497714; doi:10.1371/journal.pone.0130810)
Supplement: S1 File — A pdf file of the Taxonomy report generated through the tBLASTn search with PghP (as described in Methods); the four hits from the B. subtilis subsp subtilis strain 168 paralogues are highlighted. (PDF) [file pone.0130810.s001.pdf]

Lineage Report (From NCBI BLAST)

. Caudovirales			[viruses]	
. . unclassified Caudovirales			[viruses]	
. . . Bacillus phage phiNIT1	406	1 hit	[viruses]	Bacillus phage phiNIT1 DNA, complete genome
. . . Bacillus phage Grass	398	1 hit	[viruses]	Bacillus phage Grass, complete genome
. . Bacillus phage PM1	192	1 hit	[viruses]	Bacillus phage PM1 DNA, complete genome
. . Bacillus phage Bobb	186	1 hit	[viruses]	Bacillus phage Bobb, complete genome
. . Bacillus phage phiAGATE	182	1 hit	[viruses]	Bacillus phage phiAGATE, complete genome
. . Bacillus phage SPP1	162	1 hit	[viruses]	Bacillus phage SPP1, complete genome
. . Bacillus phage SP10	144	1 hit	[viruses]	Bacillus phage SP10, complete genome
. . Bacillus phage SPbeta	127	1 hit	[viruses]	Bacillus phage SPBc2, complete genome
. . Staphylococcus phage StB20	123	1 hit	[viruses]	Staphylococcus phage StB20, complete genome
. . Bacillus phage CampHawk	111	1 hit	[viruses]	Bacillus phage CampHawk, complete genome
. . Bacillus phage SPO1	98	1 hit	[viruses]	Bacillus phage SPO1, complete genome
. Bacillus sp. FJAT-14515	167	1 hit	[firmicutes]	Bacillus sp. FJAT-14515 Scaffold2_1, whole genome shotgun s
. Bacillus sp. DW5-4	152	2 hits	[firmicutes]	Bacillus sp. DW5-4 contig12, whole genome shotgun sequence
. Bacillus pumilus SAFR-032	151	1 hit	[firmicutes]	Bacillus pumilus SAFR-032 chromosome, complete genome
. Bacillus sp. 171095_106	151	3 hits	[firmicutes]	Bacillus sp. 171095_106 H139DRAFT_scaffold00003.3_C, whole
. Bacillus xiamenensis	151	2 hits	[firmicutes]	Bacillus xiamenensis strain HYC-10 contig31, whole genome s
. Bacillus megaterium WSH-002	150	1 hit	[firmicutes]	Bacillus megaterium WSH-002 chromosome, complete genome
. Bacillus pumilus	150	5 hits	[firmicutes]	Bacillus pumilus strain 3-19 contig00006, whole genome shot
. Bacillus sp. JGI 001006-L10	150	2 hits	[firmicutes]	Bacillus sp. JGI 001006-L10 C598DRAFT_contig_0.1_C, whole g
. Bacillus megaterium DSM 319	150	1 hit	[firmicutes]	Bacillus megaterium DSM 319 chromosome, complete genome
. Bacillus megaterium NBRC 15308 = ATCC 14581	150	4 hits	[firmicutes]	Bacillus megaterium NBRC 15308 = ATCC 14581 Cont154, whole
. Bacillus megaterium	150	1 hit	[firmicutes]	Bacillus megaterium strain BMS scaffold1, whole genome shot
. Bacillus megaterium NCT-2	149	4 hits	[firmicutes]	Bacillus megaterium NCT-2 contig53, whole genome shotgun se
. Bacillus pumilus ATCC 7061	149	1 hit	[firmicutes]	Bacillus pumilus ATCC 7061 BAT.Contig118, whole genome shot
. Bacillus pumilus S-1	149	2 hits	[firmicutes]	Bacillus pumilus S-1 scaffold52, whole genome shotgun seque
. Bacillus sp. Aph1	148	4 hits	[firmicutes]	Bacillus sp. Aph1 Contig122, whole genome shotgun sequence
. Bacillus aryabhattai	148	3 hits	[firmicutes]	Bacillus aryabhattai strain GZ03 contig1_scaffold1, whole g
. Bacillus flexus 27Col1.1E	148	3 hits	[firmicutes]	Bacillus flexus 27Col1.1E H543DRAFT_contig000009.9_C, whole
. Bacillus sp. RP1137	147	1 hit	[firmicutes]	Bacillus sp. RP1137 contig_10, whole genome shotgun sequence
. Bacillus megaterium QM B1551	147	1 hit	[firmicutes]	Bacillus megaterium QM B1551 chromosome, complete genome
. Bacillus altitudinis	147	2 hits	[firmicutes]	Bacillus altitudinis strain B-388 contig00013, whole genome
. Bacillus pumilus INR7	147	2 hits	[firmicutes]	Bacillus pumilus INR7 contig44, whole genome shotgun sequen
. Bacillus pumilus BA06	147	1 hit	[firmicutes]	Bacillus pumilus BA06 BA_1108, whole genome shotgun sequence
. Bacillus altitudinis 41KF2b	147	1 hit	[firmicutes]	Bacillus altitudinis 41KF2b contig1, whole genome shotgun s
. Bacillus sp. UNC125MFCrub1.1	147	1 hit	[firmicutes]	Bacillus sp. UNC125MFCrub1.1 N521DRAFT_scaffold00001.1, who
. Bacillus sp. 278922_107	145	2 hits	[firmicutes]	Bacillus sp. 278922_107 H622DRAFT_scaffold00001.1, whole ge
. Bacillus safensis FO-36b	144	1 hit	[firmicutes]	Bacillus safensis FO-36b contig3, whole genome shotgun sequ
. Bacillus safensis VK	144	2 hits	[firmicutes]	Bacillus safensis VK contig00009, whole genome shotgun sequ
. Bacillus pumilus CCMA-560	144	2 hits	[firmicutes]	Bacillus pumilus CCMA-560 NODE_36.3_length_190507_cov_801.7
. Bacillus safensis	144	2 hits	[firmicutes]	Bacillus safensis strain CFA06 contig_40, whole genome shot
. Bacillus firmus DS1	140	1 hit	[firmicutes]	Bacillus firmus DS1 scaffold12, whole genome shotgun sequen
. Bacillus licheniformis G-1	138	2 hits	[firmicutes]	Bacillus licheniformis G-1 CONTIG_30, whole genome shotgun
. Bacillus sp. CPSM8	138	3 hits	[firmicutes]	Bacillus sp. CPSM8 SM8.a5.scaffold1.1, whole genome shotgun
. Bacillus sp. MSP5.4	138	3 hits	[firmicutes]	Bacillus sp. MSP5.4 contig00003, whole genome shotgun seque
. Bacillus sp. SB47	138	1 hit	[firmicutes]	Bacillus sp. SB47 scaffold00001, whole genome shotgun seque
. Bacillus licheniformis CG-B52	137	3 hits	[firmicutes]	Bacillus licheniformis CG-B52 scaffold39, whole genome shot
. Bacillus licheniformis 10-1-A	137	3 hits	[firmicutes]	Bacillus licheniformis 10-1-A contig000022, whole genome sh
. Bacillus licheniformis 5-2-D	137	3 hits	[firmicutes]	Bacillus licheniformis 5-2-D contig000043, whole genome sho
. Bacillus licheniformis F2-1	137	2 hits	[firmicutes]	Bacillus licheniformis F2-1 CONTIG_35, whole genome shotgun
. Bacillus licheniformis F1-1	137	2 hits	[firmicutes]	Bacillus licheniformis F1-1 CONTIG_01, whole genome shotgun
. Bacillus licheniformis	137	3 hits	[firmicutes]	Bacillus licheniformis strain 3F-3 NODE_1_length_1486511_co
. Bacillus licheniformis WX-02	137	1 hit	[firmicutes]	Bacillus licheniformis WX-02 MUY_1, whole genome shotgun se
. Bacillus licheniformis DSM 13 = ATCC 14580	137	2 hits	[firmicutes]	Bacillus licheniformis ATCC 14580 chromosome, complete geno

Bacillus sp. BT1B_CT2	137	1 hit	[firmicutes]	Bacillus sp. BT1B_CT2 supercont1.1, whole genome shotgun se
Staphylococcus aureus subsp. aureus CO-98	136	4 hits	[firmicutes]	Staphylococcus aureus subsp. aureus CO-98 contig00043, whol
Bacillus licheniformis 9945A	136	1 hit	[firmicutes]	Bacillus licheniformis 9945A, complete genome
Bacillus cereus AH1272	135	1 hit	[firmicutes]	Bacillus cereus AH1272 chromosome, whole genome shotgun seq
Bacillus cereus AH1273	135	1 hit	[firmicutes]	Bacillus cereus AH1273 chromosome, whole genome shotgun seq
Bacillus atrophaeus UCMB-5137	135	1 hit	[firmicutes]	Bacillus atrophaeus UCMB-5137 chromosome, whole genome shot
Bacillus sp. NSP9.1	135	1 hit	[firmicutes]	Bacillus sp. NSP9.1 scaffold00001, whole genome shotgun seq
Staphylococcus massiliensis CCUG 55927	135	1 hit	[firmicutes]	Staphylococcus massiliensis CCUG 55927 scaffold00001, whole
Bacillus licheniformis CGMCC 3963	134	3 hits	[firmicutes]	Bacillus licheniformis CGMCC 3963 contigs5, whole genome sh
Bacillus atrophaeus C89	134	3 hits	[firmicutes]	Bacillus atrophaeus C89 contig000079, whole genome shotgun
Bacillus atrophaeus BACI051-N	134	3 hits	[firmicutes]	Bacillus atrophaeus BACI051-N contig00014, whole genome sho
Bacillus atrophaeus BACI051-E	134	2 hits	[firmicutes]	Bacillus atrophaeus BACI051-E contig00023, whole genome sho
Bacillus atrophaeus 1013-2	134	3 hits	[firmicutes]	Bacillus atrophaeus 1013-2 contig00024, whole genome shotgu
Bacillus atrophaeus ATCC 49822-1	134	2 hits	[firmicutes]	Bacillus atrophaeus ATCC 49822-1 contig00033, whole genome
Bacillus atrophaeus ATCC 9372-2	134	3 hits	[firmicutes]	Bacillus atrophaeus ATCC 9372-2 contig00006, whole genome s
Bacillus atrophaeus ATCC 9372-1	134	2 hits	[firmicutes]	Bacillus atrophaeus ATCC 9372-1 contig00013, whole genome s
Bacillus atrophaeus Detrick-2	134	3 hits	[firmicutes]	Bacillus atrophaeus Detrick-2 contig00028, whole genome sho
Bacillus atrophaeus ATCC 49822-2	134	2 hits	[firmicutes]	Bacillus atrophaeus ATCC 49822-2 contig00002, whole genome
Bacillus atrophaeus Detrick-1	134	2 hits	[firmicutes]	Bacillus atrophaeus Detrick-1 contig00027, whole genome sho
Bacillus atrophaeus Detrick-3	134	2 hits	[firmicutes]	Bacillus atrophaeus Detrick-3 contig00006, whole genome sho
Bacillus atrophaeus 1013-1	134	1 hit	[firmicutes]	Bacillus atrophaeus 1013-1 contig00010, whole genome shotgu
Bacillus subtilis subsp. niger	134	1 hit	[firmicutes]	Bacillus subtilis subsp. niger strain PCI 246 scaffold1, wh
Bacillus atrophaeus 1942	134	1 hit	[firmicutes]	Bacillus atrophaeus 1942 chromosome, complete genome
Bacillus atrophaeus subsp. globigii	134	1 hit	[firmicutes]	Bacillus atrophaeus subsp. globigii strain BSS
Staphylococcus epidermidis NIHLM021	133	5 hits	[firmicutes]	Staphylococcus epidermidis NIHLM021 HMPREF9982_contig00053,
Bacillus sonorensis NBRC 101234 = KCTC 13918	132	3 hits	[firmicutes]	Bacillus sonorensis NBRC 101234 = KCTC 13918 contig55, whol
Bacillus endophyticus 2102	131	2 hits	[firmicutes]	Bacillus endophyticus 2102 contig52, whole genome shotgun s
Streptomyces roseochromogenus subsp. oscitans DS 12.976	131	1 hit	[high GC Gram+]	Streptomyces roseochromogenus subsp. oscitans DS 12.976 chr
Bacillus subtilis subsp. subtilis str. SC-8	129	3 hits	[firmicutes]	Bacillus subtilis subsp. subtilis str. SC-8 92_9, whole gen
Bacillus subtilis	127	10 hits	[firmicutes]	Bacillus subtilis strain KATMIRA1933 B_sub_1933_089, whole
Bacillus siamensis KCTC 13613	127	1 hit	[firmicutes]	Bacillus siamensis KCTC 13613 contig13, whole genome shotgu
Bacillus subtilis MB73/2	127	2 hits	[firmicutes]	Bacillus subtilis MB73/2 bacsub.contig.3, whole genome shot
Bacillus sp. EGD-AK10	127	1 hit	[firmicutes]	Bacillus sp. EGD-AK10 contig1, whole genome shotgun sequence
Bacillus subtilis E1	127	2 hits	[firmicutes]	Bacillus subtilis E1, whole genome shotgun sequence
**Bacillus subtilis subsp. subtilis str. 168**	**127**	**4 hits**	**[firmicutes]**	**Bacillus subtilis subsp. subtilis str. 168 scaffold12, whol**
Bacillus subtilis QB928	127	1 hit	[firmicutes]	Bacillus subtilis QB928 chromosome, complete genome
Bacillus subtilis subsp. subtilis str. JH642	127	1 hit	[firmicutes]	Bacillus subtilis subsp. subtilis str. JH642 chromosome, wh
Bacillus subtilis subsp. subtilis str. NCIB 3610	127	1 hit	[firmicutes]	Bacillus subtilis subsp. subtilis str. NCIB 3610 chromosome
Bacillus subtilis subsp. subtilis str. SMY	127	1 hit	[firmicutes]	Bacillus subtilis subsp. subtilis str. SMY chromosome, whol
Bacillus subtilis subsp. subtilis 6051-HGW	127	1 hit	[firmicutes]	Bacillus subtilis subsp. subtilis 6051-HGW, complete genome
Bacillus subtilis PS216	124	5 hits	[firmicutes]	Bacillus subtilis PS216 ctg7180000000298, whole genome shot
Bacillus subtilis Hal1	127	3 hits	[firmicutes]	Bacillus subtilis Hal1 Contig130, whole genome shotgun sequ
Staphylococcus epidermidis NIHLM040	127	4 hits	[firmicutes]	Staphylococcus epidermidis NIHLM040 HMPREF9986_contig00025,
Bacillus subtilis subsp. spizizenii DV1-B-1	126	1 hit	[firmicutes]	Bacillus subtilis subsp. spizizenii DV1-B-1 gbs3c.contig.14
Prauserella sp. Am3	126	1 hit	[high GC Gram+]	Prauserella sp. Am3 HQ32_scaffold_11.12, whole genome shotg
Bacillus amyloliquefaciens TA208	126	1 hit	[firmicutes]	Bacillus amyloliquefaciens TA208 chromosome, complete genome
Bacillus amyloliquefaciens XH7	126	1 hit	[firmicutes]	Bacillus amyloliquefaciens XH7 chromosome, complete genome
Bacillus amyloliquefaciens DSM 7	126	1 hit	[firmicutes]	Bacillus amyloliquefaciens DSM 7, complete genome
Bacillus amyloliquefaciens LL3	126	1 hit	[firmicutes]	Bacillus amyloliquefaciens LL3 chromosome, complete genome
Prauserella rugosa	126	1 hit	[high GC Gram+]	Prauserella rugosa strain NRRL B-2295 contig1.1, whole geno
Bacillus amyloliquefaciens CC178	126	1 hit	[firmicutes]	Bacillus amyloliquefaciens CC178, complete genome
Bacillus amyloliquefaciens subsp. plantarum str. FZB42	126	1 hit	[firmicutes]	Bacillus amyloliquefaciens FZB42, complete genome
Bacillus amyloliquefaciens	125	6 hits	[firmicutes]	Bacillus amyloliquefaciens strain LPL-K103 contig_8, whole
Bacillus sp. MSP13	125	2 hits	[firmicutes]	Bacillus sp. MSP13 contig00006, whole genome shotgun sequen
Bacillus vallismortis DV1-F-3	125	3 hits	[firmicutes]	Bacillus vallismortis DV1-F-3 scf7180000000934, whole genom
Staphylococcus epidermidis APO27	124	3 hits	[firmicutes]	Staphylococcus epidermidis APO27 CONTIG_219, whole genome s
Staphylococcus epidermidis VCU014	125	4 hits	[firmicutes]	Staphylococcus epidermidis VCU014 contig00023, whole genome

. Staphylococcus epidermidis VCU013	123	4 hits	[firmicutes]	Staphylococcus epidermidis VCU013 contig00065, whole genome
. Bacillus subtilis GB03	125	1 hit	[firmicutes]	Bacillus subtilis GB03 contig9, whole genome shotgun sequen
. Bacillus sp. 5B6	125	1 hit	[firmicutes]	Bacillus sp. 5B6 5B6, whole genome shotgun sequence
. Bacillus subtilis gtP20b	124	3 hits	[firmicutes]	Bacillus subtilis gtP20b, whole genome shotgun sequence
. Bacillus amyloliquefaciens HB-26	125	2 hits	[firmicutes]	Bacillus amyloliquefaciens HB-26 contig6, whole genome shot
. Bacillus amyloliquefaciens subsp. plantarum	125	5 hits	[firmicutes]	Bacillus amyloliquefaciens subsp. plantarum strain W2 rawli
. Bacillus amyloliquefaciens subsp. plantarum UCMB5036	125	1 hit	[firmicutes]	Bacillus amyloliquefaciens subsp. plantarum UCMB5036 comple
. Bacillus subtilis subsp. inaquosorum KCTC 13429	124	2 hits	[firmicutes]	Bacillus subtilis subsp. inaquosorum KCTC 13429 14.BSI.1_4,
. Laceyella sacchari 1-1	124	1 hit	[firmicutes]	Laceyella sacchari 1-1 GS_11_TGACCA_contig_19, whole genome
. Staphylococcus haemolyticus R1P1	124	2 hits	[firmicutes]	Staphylococcus haemolyticus R1P1 contig00007, whole genome
. Staphylococcus haemolyticus DNF00585	124	2 hits	[firmicutes]	Staphylococcus haemolyticus DNF00585 contig02, whole genome
. Bacillus subtilis subsp. subtilis str. AUSI98	124	4 hits	[firmicutes]	Bacillus subtilis subsp. subtilis str. AUSI98 contig7180000
. Staphylococcus capitis C87	124	2 hits	[firmicutes]	Staphylococcus capitis C87 supercont1.6, whole genome shotg
. Staphylococcus haemolyticus	124	10 hits	[firmicutes]	Staphylococcus haemolyticus strain C10D contig_9, whole gen
. Bacillus subtilis subsp. subtilis str. MP9	124	3 hits	[firmicutes]	Bacillus subtilis subsp. subtilis str. MP9 Contig170, whole
. Bacillus amyloliquefaciens Y2	124	1 hit	[firmicutes]	Bacillus amyloliquefaciens Y2 chromosome, complete genome
. Bacillus amyloliquefaciens subsp. plantarum YAU B9601-Y2	124	1 hit	[firmicutes]	Bacillus amyloliquefaciens subsp. plantarum YAU B9601-Y2, c
. Bacillus amyloliquefaciens subsp. plantarum NAU-B3	124	1 hit	[firmicutes]	Bacillus amyloliquefaciens subsp. plantarum NAU-B3, complet
. Bacillus subtilis SPZ1	124	4 hits	[firmicutes]	Bacillus subtilis SPZ1 contig11, whole genome shotgun seque
. Paenibacillus polymyxa ATCC 12321	124	4 hits	[firmicutes]	Paenibacillus polymyxa ATCC 12321 contig11_scaffold9, whole
. Bacillus amyloliquefaciens EGD-AQ14	124	2 hits	[firmicutes]	Bacillus amyloliquefaciens EGD-AQ14 contig1, whole genome s
. Bacillus subtilis subsp. subtilis str. MP11	124	3 hits	[firmicutes]	Bacillus subtilis subsp. subtilis str. MP11 Contig212, whol
. Bacillus subtilis subsp. subtilis str. BAB-1	124	1 hit	[firmicutes]	Bacillus subtilis subsp. subtilis str. BAB-1, complete geno
. Bacillus subtilis XF-1	124	1 hit	[firmicutes]	Bacillus subtilis XF-1, complete genome
. Bacillus subtilis S1-4	124	3 hits	[firmicutes]	Bacillus subtilis S1-4 S1-4_3, whole genome shotgun sequence
. Sporosarcina pasteurii NCIM 2477	124	3 hits	[firmicutes]	Sporosarcina pasteurii NCIM 2477 contig_6, whole genome sho
. Bacillus subtilis subsp. subtilis	124	4 hits	[firmicutes]	Bacillus subtilis subsp. subtilis strain NDmed contig_2, wh
. Bacillus subtilis PTS-394	124	1 hit	[firmicutes]	Bacillus subtilis PTS-394 contig26, whole genome shotgun se
. Bacillus subtilis QH-1	124	1 hit	[firmicutes]	Bacillus subtilis QH-1 contig3, whole genome shotgun sequen
. Bacillus subtilis PY79	124	1 hit	[firmicutes]	Bacillus subtilis PY79, complete genome
. Bacillus subtilis subsp. subtilis str. BSP1	124	1 hit	[firmicutes]	Bacillus subtilis subsp. subtilis str. BSP1 chromosome, com
. Bacillus subtilis BSn5	124	1 hit	[firmicutes]	Bacillus subtilis BSn5 chromosome, complete genome
. Staphylococcus epidermidis APO35	123	6 hits	[firmicutes]	Staphylococcus epidermidis APO35 genomic scaffold APO35_sca
. Staphylococcus epidermidis Scl22	123	3 hits	[firmicutes]	Staphylococcus epidermidis Scl22 Scl22_scaffold137, whole g
. Staphylococcus epidermidis MC28	123	3 hits	[firmicutes]	Staphylococcus epidermidis MC28 CONTIG_106, whole genome sh
. Staphylococcus epidermidis MC16	123	3 hits	[firmicutes]	Staphylococcus epidermidis MC16 CONTIG_87, whole genome sho
. Staphylococcus epidermidis CIM37	123	3 hits	[firmicutes]	Staphylococcus epidermidis CIM37 CONTIG_85, whole genome sh
. Staphylococcus epidermidis MC19	123	3 hits	[firmicutes]	Staphylococcus epidermidis MC19 CONTIG_34, whole genome sho
. Staphylococcus epidermidis CIM40	123	3 hits	[firmicutes]	Staphylococcus epidermidis CIM40 CONTIG_15, whole genome sh
. Staphylococcus epidermidis Scl19	123	2 hits	[firmicutes]	Staphylococcus epidermidis Scl19 CONTIG_25, whole genome sh
. Staphylococcus epidermidis FRI909	123	3 hits	[firmicutes]	Staphylococcus epidermidis FRI909 contig00033, whole genome
. Staphylococcus epidermidis WI05	123	3 hits	[firmicutes]	Staphylococcus epidermidis WI05 CONTIG_168, whole genome sh
. Staphylococcus epidermidis WI09	123	1 hit	[firmicutes]	Staphylococcus epidermidis WI09 CONTIG_55, whole genome sho
. Staphylococcus epidermidis CIM28	123	1 hit	[firmicutes]	Staphylococcus epidermidis CIM28 CONTIG_137, whole genome s
. Staphylococcus epidermidis VCU144	123	4 hits	[firmicutes]	Staphylococcus epidermidis VCU144 contig00088, whole genome
. Staphylococcus haemolyticus JCSC1435	123	1 hit	[firmicutes]	Staphylococcus haemolyticus JCSC1435 chromosome, complete g
. Staphylococcus epidermidis 12142587	123	5 hits	[firmicutes]	Staphylococcus epidermidis 12142587 contig00004, whole geno
. Bacillus subtilis subsp. spizizenii ATCC 6633	123	2 hits	[firmicutes]	Bacillus subtilis subsp. spizizenii ATCC 6633 contig00011,
. Staphylococcus epidermidis VCU065	123	4 hits	[firmicutes]	Staphylococcus epidermidis VCU065 contig00022, whole genome
. Staphylococcus epidermidis VCU123	123	4 hits	[firmicutes]	Staphylococcus epidermidis VCU123 contig00035, whole genome
. Bacillus subtilis subsp. spizizenii str. W23	123	1 hit	[firmicutes]	Bacillus subtilis subsp. spizizenii str. W23 chromosome, co
. Staphylococcus epidermidis	123	13 hits	[firmicutes]	Staphylococcus epidermidis strain 1457 1457_contig_1, whole
. Staphylococcus epidermidis NIHLM015	123	3 hits	[firmicutes]	Staphylococcus epidermidis NIHLM015 HMPREF9978_contig00004,
. Staphylococcus epidermidis M23864:W2(grey)	123	2 hits	[firmicutes]	Staphylococcus epidermidis M23864:W2(grey) SCAFFOLD1, whole
. Bacillus sp. JS	123	1 hit	[firmicutes]	Bacillus sp. JS chromosome, complete genome
. Bacillus methylotrophicus SK19.001	122	3 hits	[firmicutes]	Bacillus methylotrophicus SK19.001 contig20_scaffold11, who
. Bacillus amyloliquefaciens subsp. amyloliquefaciens DC-12	122	1 hit	[firmicutes]	Bacillus amyloliquefaciens subsp. amyloliquefaciens DC-12 S

. Staphylococcus epidermidis NIHLM037	122	4 hits	[firmicutes]	Staphylococcus epidermidis NIHLM037 HMPREF9984_contig00004,
. Bacillus subtilis Miyagi-4	122	3 hits	[firmicutes]	Bacillus subtilis Miyagi-4, whole genome shotgun sequence
. Halobacillus sp. BBL2006	122	1 hit	[firmicutes]	Halobacillus sp. BBL2006 cont499, whole genome shotgun sequ
. Staphylococcus epidermidis VCU071	122	3 hits	[firmicutes]	Staphylococcus epidermidis VCU071 contig00006, whole genome
. Bacillus subtilis subsp. subtilis str. RO-NN-1	122	1 hit	[firmicutes]	Bacillus subtilis subsp. subtilis str. RO-NN-1 chromosome,
. Bacillus subtilis subsp. natto BEST195	122	1 hit	[firmicutes]	Bacillus subtilis subsp. natto BEST195 DNA, complete genome
. Staphylococcus epidermidis NIHLM031	122	3 hits	[firmicutes]	Staphylococcus epidermidis NIHLM031 HMPREF9980_contig00091,
. Staphylococcus sp. EGD-HP3	122	2 hits	[firmicutes]	Staphylococcus sp. EGD-HP3 contig3, whole genome shotgun se
. Bacillus tequilensis KCTC 13622	122	1 hit	[firmicutes]	Bacillus tequilensis KCTC 13622 contig41, whole genome shot
. Staphylococcus warneri Lyso 1 2011	122	3 hits	[firmicutes]	Staphylococcus warneri Lyso 1 2011 psalyso111.contig.19, wh
. Staphylococcus warneri Lyso 2 2011	122	3 hits	[firmicutes]	Staphylococcus warneri Lyso 2 2011 psaLyso211.contig.18, wh
. Staphylococcus warneri	122	2 hits	[firmicutes]	Staphylococcus warneri strain NGS-ED-1001 Contig_2, whole g
. Staphylococcus warneri SG1	122	1 hit	[firmicutes]	Staphylococcus warneri SG1, complete genome
. Streptomyces aureocirculatus	122	4 hits	[high GC Gram+]	Streptomyces aureocirculatus strain NRRL ISP-5386 contig23.
. Staphylococcus epidermidis UC7032	122	3 hits	[firmicutes]	Staphylococcus epidermidis UC7032 NODE_40_length_56474_cov_
. Staphylococcus warneri A487	122	3 hits	[firmicutes]	Staphylococcus warneri A487, whole genome shotgun sequence
. Bacillus sp. UNC69MF	122	1 hit	[firmicutes]	Bacillus sp. UNC69MF BR62DRAFT_scaffold00001.1, whole genom
. Bacillus amyloliquefaciens subsp. plantarum AS43.3	122	1 hit	[firmicutes]	Bacillus amyloliquefaciens subsp. plantarum AS43.3 chromoso
. Staphylococcus pasteuri SP1	122	1 hit	[firmicutes]	Staphylococcus pasteuri SP1, complete genome
. Bacillus mojavensis RO-H-1 = KCTC 3706	122	4 hits	[firmicutes]	Bacillus mojavensis RO-H-1 = KCTC 3706 strain RO-H-1 gbs2c.
. Bacillus amyloliquefaciens UASWS BA1	122	1 hit	[firmicutes]	Bacillus amyloliquefaciens UASWS BA1 Contig002, whole genom
. Bacillus amyloliquefaciens subsp. plantarum M27	122	1 hit	[firmicutes]	Bacillus amyloliquefaciens subsp. plantarum M27 contig10, w
. Staphylococcus epidermidis VCU118	122	3 hits	[firmicutes]	Staphylococcus epidermidis VCU118 contig00002, whole genome
. Bacillus amyloliquefaciens IT-45	122	1 hit	[firmicutes]	Bacillus amyloliquefaciens IT-45, complete genome
. Bacillus amyloliquefaciens LFB112	122	1 hit	[firmicutes]	Bacillus amyloliquefaciens LFB112, complete genome
. Bacillus sp. 916	122	2 hits	[firmicutes]	Bacillus sp. 916 Contig349, whole genome shotgun sequence
. Bacillus amyloliquefaciens EBL11	122	2 hits	[firmicutes]	Bacillus amyloliquefaciens EBL11 NODE_1, whole genome shotg
. Bacillus amyloliquefaciens subsp. plantarum UCMB5113	122	1 hit	[firmicutes]	Bacillus amyloliquefaciens subsp. plantarum UCMB5113, compl
. Bacillus amyloliquefaciens subsp. plantarum UCMB5033	122	1 hit	[firmicutes]	Bacillus amyloliquefaciens subsp. plantarum UCMB5033, compl
. Staphylococcus arlettae CVD059	122	3 hits	[firmicutes]	Staphylococcus arlettae CVD059 SARL_c69, whole genome shotg
. Staphylococcus epidermidis VCU129	121	3 hits	[firmicutes]	Staphylococcus epidermidis VCU129 contig00007, whole genome
. Bacillus subtilis subsp. spizizenii TU-B-10	121	1 hit	[firmicutes]	Bacillus subtilis subsp. spizizenii TU-B-10 chromosome, com
. Bacillus amyloliquefaciens subsp. plantarum CAU B946	121	1 hit	[firmicutes]	Bacillus amyloliquefaciens subsp. plantarum CAU B946, compl
. Staphylococcus epidermidis VCU128	121	3 hits	[firmicutes]	Staphylococcus epidermidis VCU128 contig00050, whole genome
. Staphylococcus epidermidis NIHLM049	121	4 hits	[firmicutes]	Staphylococcus epidermidis NIHLM049 HMPREF9987_contig00058,
. Staphylococcus epidermidis VCU041	121	4 hits	[firmicutes]	Staphylococcus epidermidis VCU041 contig00018, whole genome
. Staphylococcus epidermidis NIH05001	121	3 hits	[firmicutes]	Staphylococcus epidermidis NIH05001 HMPREF9973_contig00029,
. Staphylococcus warneri L37603	120	3 hits	[firmicutes]	Staphylococcus warneri L37603 contig00005, whole genome sho
. Bacillus mojavensis RRC 101	120	3 hits	[firmicutes]	Bacillus mojavensis RRC 101 contig_48, whole genome shotgun
. Staphylococcus epidermidis NIHLM061	119	3 hits	[firmicutes]	Staphylococcus epidermidis NIHLM061 HMPREF9990_contig00064,
. Staphylococcus epidermidis NIHLM023	119	3 hits	[firmicutes]	Staphylococcus epidermidis NIHLM023 HMPREF9983_contig00238,
. Staphylococcus epidermidis W23144	119	2 hits	[firmicutes]	Staphylococcus epidermidis W23144 SCAFFOLD2, whole genome s
. Staphylococcus capitis VCU116	119	3 hits	[firmicutes]	Staphylococcus capitis VCU116 contig00022, whole genome sho
. Staphylococcus epidermidis ATCC 12228	119	1 hit	[firmicutes]	Staphylococcus epidermidis ATCC 12228 chromosome, complete
. Staphylococcus epidermidis NIH06004	119	3 hits	[firmicutes]	Staphylococcus epidermidis NIH06004 HMPREF1389_contig00010,
. Staphylococcus epidermidis NIH08001	119	3 hits	[firmicutes]	Staphylococcus epidermidis NIH08001 HMPREF1390_contig00028,
. Staphylococcus epidermidis M0026	119	3 hits	[firmicutes]	Staphylococcus epidermidis M0026 adAAZ-supercont1.10, whole
. Staphylococcus epidermidis VCU045	118	3 hits	[firmicutes]	Staphylococcus epidermidis VCU045 contig00083, whole genome
. Staphylococcus epidermidis NIH04003	118	3 hits	[firmicutes]	Staphylococcus epidermidis NIH04003 HMPREF1387_contig00028,
. Staphylococcus epidermidis VCU037	118	3 hits	[firmicutes]	Staphylococcus epidermidis VCU037 contig00006, whole genome
. Bacillus flexus T6186-2	118	2 hits	[firmicutes]	Bacillus flexus T6186-2 contig_167, whole genome shotgun se
. Staphylococcus epidermidis M0881	118	3 hits	[firmicutes]	Staphylococcus epidermidis M0881 acumm-supercont1.6, whole
. Streptomyces sp. PRh5	118	1 hit	[high GC Gram+]	Streptomyces sp. PRh5 contig003, whole genome shotgun seque
. Staphylococcus epidermidis NIHLM001	118	4 hits	[firmicutes]	Staphylococcus epidermidis NIHLM001 HMPREF9975_contig00064,
. Staphylococcus epidermidis RP62A	118	1 hit	[firmicutes]	Staphylococcus epidermidis RP62A, complete genome
. Marinococcus halotolerans DSM 16375	117	1 hit	[firmicutes]	Marinococcus halotolerans DSM 16375 G548DRAFT_scaffold00015
. Staphylococcus sp. URHA0057	117	3 hits	[firmicutes]	Staphylococcus sp. URHA0057 N524DRAFT_scaffold00003.3, whol

. Staphylococcus xylosus NJ	117	2 hits	[firmicutes]	Staphylococcus xylosus NJ contig19, whole genome shotgun se
. Bacillus panaciterrae DSM 19096	117	1 hit	[firmicutes]	Bacillus panaciterrae DSM 19096 H540DRAFT_scaffold00002.2_C
. Staphylococcus capitis	117	3 hits	[firmicutes]	Staphylococcus capitis strain LNZR-1 contig_79, whole genom
. Staphylococcus capitis CR01	117	1 hit	[firmicutes]	Staphylococcus capitis CR01, whole genome shotgun sequence
. Staphylococcus epidermidis NIHLM003	117	3 hits	[firmicutes]	Staphylococcus epidermidis NIHLM003 HMPREF9976_contig00027,
. Nocardiopsis xinjiangensis YIM 90004	117	1 hit	[high GC Gram+]	Nocardiopsis xinjiangensis YIM 90004 contig_6, whole genome
. Staphylococcus epidermidis NIHLM053	117	3 hits	[firmicutes]	Staphylococcus epidermidis NIHLM053 HMPREF9988_contig00125,
. Staphylococcus epidermidis NIHLM057	117	3 hits	[firmicutes]	Staphylococcus epidermidis NIHLM057 HMPREF9989_contig00235,
. Staphylococcus sp. JGI 0001002-I23	117	3 hits	[firmicutes]	Staphylococcus sp. JGI 0001002-I23 B135DRAFT_NODE-unique_23
. Staphylococcus epidermidis NIHLM070	117	3 hits	[firmicutes]	Staphylococcus epidermidis NIHLM070 HMPREF9992_contig00009,
. Staphylococcus sp. MDS7B	117	5 hits	[firmicutes]	Staphylococcus sp. MDS7B Ion2_c59, whole genome shotgun seq
. Staphylococcus epidermidis VCU125	117	3 hits	[firmicutes]	Staphylococcus epidermidis VCU125 contig00019, whole genome
. Staphylococcus epidermidis VCU127	117	3 hits	[firmicutes]	Staphylococcus epidermidis VCU127 contig00011, whole genome
. Staphylococcus epidermidis IS-250	117	3 hits	[firmicutes]	Staphylococcus epidermidis IS-250 contig00063, whole genome
. Staphylococcus epidermidis IS-K	117	3 hits	[firmicutes]	Staphylococcus epidermidis IS-K contig00049, whole genome s
. Staphylococcus epidermidis NIHLM008	117	3 hits	[firmicutes]	Staphylococcus epidermidis NIHLM008 HMPREF9977_contig00059,
. Staphylococcus epidermidis NIHLM088	117	3 hits	[firmicutes]	Staphylococcus epidermidis NIHLM088 HMPREF9994_contig00045,
. Staphylococcus xylosus DMB3-Bh1	117	3 hits	[firmicutes]	Staphylococcus xylosus DMB3-Bh1 DMB3-BHI_contig_6, whole ge
. Staphylococcus capitis QN1	116	3 hits	[firmicutes]	Staphylococcus capitis QN1 Contig3_2, whole genome shotgun
. Staphylococcus epidermidis BVS058A4	116	2 hits	[firmicutes]	Staphylococcus epidermidis BVS058A4 supercont1.2, whole gen
. Staphylococcus epidermidis 14.1.R1.SE	116	3 hits	[firmicutes]	Staphylococcus epidermidis 14.1.R1.SE ctg1131387704530, who
. Actinomadura oligospora ATCC 43269	115	1 hit	[high GC Gram+]	Actinomadura oligospora ATCC 43269 P696DRAFT_scaffold00024.
. Streptomyces sp. NRRL S-920	115	1 hit	[high GC Gram+]	Streptomyces sp. NRRL S-920 contig4.1, whole genome shotgun
. Staphylococcus sp. TE8	115	2 hits	[firmicutes]	Staphylococcus sp. TE8 Scaffold01, whole genome shotgun seq
. Staphylococcus epidermidis NIHLM095	115	3 hits	[firmicutes]	Staphylococcus epidermidis NIHLM095 HMPREF9995_contig00018,
. Staphylococcus epidermidis NIHLM087	115	3 hits	[firmicutes]	Staphylococcus epidermidis NIHLM087 HMPREF9993_contig00219,
. Staphylococcus chromogenes MU 970	115	1 hit	[firmicutes]	Staphylococcus chromogenes MU 970 SCHR_MU970Contig20, whole
. Staphylococcus epidermidis AG42	115	2 hits	[firmicutes]	Staphylococcus epidermidis AG42 BO14DRAFT_scf7180000000023_
. Halobacillus halophilus DSM 2266	115	1 hit	[firmicutes]	Halobacillus halophilus DSM 2266, complete genome
. Staphylococcus epidermidis Scl31	114	3 hits	[firmicutes]	Staphylococcus epidermidis Scl31 CONTIG_182, whole genome s
. Staphylococcus epidermidis VCU126	114	3 hits	[firmicutes]	Staphylococcus epidermidis VCU126 contig00036, whole genome
. Staphylococcus epidermidis VCU111	114	3 hits	[firmicutes]	Staphylococcus epidermidis VCU111 contig00031, whole genome
. Staphylococcus epidermidis VCU117	114	3 hits	[firmicutes]	Staphylococcus epidermidis VCU117 contig00009, whole genome
. Staphylococcus epidermidis NIH05003	114	3 hits	[firmicutes]	Staphylococcus epidermidis NIH05003 HMPREF1388_contig00030,
. Staphylococcus epidermidis NIHLM020	114	3 hits	[firmicutes]	Staphylococcus epidermidis NIHLM020 HMPREF9981_contig00048,
. Staphylococcus epidermidis NIH051668	114	3 hits	[firmicutes]	Staphylococcus epidermidis NIH051668 HMPREF1386_contig00027
. Staphylococcus epidermidis NIHLM039	114	3 hits	[firmicutes]	Staphylococcus epidermidis NIHLM039 HMPREF9985_contig00068,
. Staphylococcus epidermidis VCU105	114	3 hits	[firmicutes]	Staphylococcus epidermidis VCU105 contig00024, whole genome
. Staphylococcus epidermidis VCU120	114	3 hits	[firmicutes]	Staphylococcus epidermidis VCU120 contig00007, whole genome
. Staphylococcus epidermidis NIHLM067	114	3 hits	[firmicutes]	Staphylococcus epidermidis NIHLM067 HMPREF9991_contig00040,
. Staphylococcus epidermidis NIHLM018	114	3 hits	[firmicutes]	Staphylococcus epidermidis NIHLM018 HMPREF9979_contig00047,
. Staphylococcus epidermidis NIH05005	114	3 hits	[firmicutes]	Staphylococcus epidermidis NIH05005 HMPREF9974_contig00039,
. Staphylococcus epidermidis VCU050	114	3 hits	[firmicutes]	Staphylococcus epidermidis VCU050 contig00043, whole genome
. Staphylococcus epidermidis Scl25	114	2 hits	[firmicutes]	Staphylococcus epidermidis Scl25 CONTIG_82, whole genome sh
. Staphylococcus epidermidis VCU109	114	3 hits	[firmicutes]	Staphylococcus epidermidis VCU109 contig00032, whole genome
. Staphylococcus epidermidis VCU081	114	3 hits	[firmicutes]	Staphylococcus epidermidis VCU081 contig00008, whole genome
. Staphylococcus epidermidis VCU036	114	3 hits	[firmicutes]	Staphylococcus epidermidis VCU036 contig00039, whole genome
. Staphylococcus epidermidis AU12-03	114	3 hits	[firmicutes]	Staphylococcus epidermidis AU12-03 contig00033, whole genom
. Staphylococcus epidermidis VCU028	114	3 hits	[firmicutes]	Staphylococcus epidermidis VCU028 contig00052, whole genome
. Staphylococcus epidermidis NIH051475	114	3 hits	[firmicutes]	Staphylococcus epidermidis NIH051475 HMPREF1385_contig00038
. Staphylococcus epidermidis SK135	114	3 hits	[firmicutes]	Staphylococcus epidermidis SK135 ctg1119966626625, whole ge
. Staphylococcus epidermidis BCM-HMP0060	114	2 hits	[firmicutes]	Staphylococcus epidermidis BCM-HMP0060 SCAFFOLD1, whole gen
. Bacillus simplex BA2H3	114	1 hit	[firmicutes]	Bacillus simplex BA2H3 scaffold2, whole genome shotgun sequ
. Streptomyces sp. NRRL S-146	113	1 hit	[high GC Gram+]	Streptomyces sp. NRRL S-146 contig1.1, whole genome shotgun
. Staphylococcus lugdunensis VCU148	113	3 hits	[firmicutes]	Staphylococcus lugdunensis VCU148 contig00041, whole genome
. Staphylococcus lugdunensis VCU139	113	3 hits	[firmicutes]	Staphylococcus lugdunensis VCU139 contig00047, whole genome
. Staphylococcus lugdunensis UCIM6116	113	3 hits	[firmicutes]	Staphylococcus lugdunensis UCIM6116 adpql-supercont1.21, wh

. Staphylococcus xylosus	112	3 hits	[firmicutes]	Staphylococcus xylosus strain LSR_02N contig00021, whole ge
. Thalassobacter arenae DSM 19593	112	1 hit	[a-proteobacteria]	Thalassobacter arenae DSM 19593 Contig34, whole genome shot
. Staphylococcus lugdunensis ACS-027-V-Sch2	112	2 hits	[firmicutes]	Staphylococcus lugdunensis ACS-027-V-Sch2 supercont1.3, who
. Staphylococcus lugdunensis VCU150	112	3 hits	[firmicutes]	Staphylococcus lugdunensis VCU150 contig00052, whole genome
. Staphylococcus lugdunensis M23590	112	2 hits	[firmicutes]	Staphylococcus lugdunensis M23590 SCAFFOLD1, whole genome s
. Staphylococcus lugdunensis N920143	112	1 hit	[firmicutes]	Staphylococcus lugdunensis N920143, complete genome
. Staphylococcus lugdunensis HKU09-01	112	1 hit	[firmicutes]	Staphylococcus lugdunensis HKU09-01 chromosome, complete ge
. Staphylococcus hominis subsp. hominis ZBW5	112	3 hits	[firmicutes]	Staphylococcus hominis subsp. hominis ZBW5 Contig_1, whole
. Staphylococcus sp. M0480	112	3 hits	[firmicutes]	Staphylococcus sp. M0480 adAFb-supercont1.1, whole genome s
. Staphylococcus epidermidis E13A	112	3 hits	[firmicutes]	Staphylococcus epidermidis E13A ctg120009356314, whole geno
. Staphylococcus equorum UMC-CNS-924	111	2 hits	[firmicutes]	Staphylococcus equorum UMC-CNS-924 SEQU924Contig10, whole g
. Oceanobacillus iheyensis HTE831	111	1 hit	[firmicutes]	Oceanobacillus iheyensis HTE831 chromosome, complete genome
. Staphylococcus sp. OJ82	110	2 hits	[firmicutes]	Staphylococcus sp. OJ82 155.SOJ.1_1, whole genome shotgun s
. Halobacillus karajensis	110	1 hit	[firmicutes]	Halobacillus karajensis strain HD-03, whole genome shotgun
. Halobacillus dabanensis	110	1 hit	[firmicutes]	Halobacillus dabanensis strain HD-02, whole genome shotgun
. Halobacillus trueperi	110	1 hit	[firmicutes]	Halobacillus trueperi strain HT-01, whole genome shotgun se
. Desulfotomaculum alcoholivorax DSM 16058	110	1 hit	[firmicutes]	Desulfotomaculum alcoholivorax DSM 16058 H569DRAFT_scaffold
. Desulfobulbus sp. Tol-SR	110	1 hit	[d-proteobacteria]	Desulfobulbus sp. Tol-SR contig_163, whole genome shotgun s
. Bradyrhizobium sp. CCBAU 43298	109	1 hit	[a-proteobacteria]	Bradyrhizobium sp. CCBAU 43298 Scaffold49.contig3, whole ge
. Dinoroseobacter shibae DFL 12 = DSM 16493	108	1 hit	[a-proteobacteria]	Dinoroseobacter shibae DFL 12 chromosome, complete genome
. Staphylococcus hominis VCU122	108	2 hits	[firmicutes]	Staphylococcus hominis VCU122 contig00004, whole genome sho
. Staphylococcus hominis subsp. hominis C80	108	1 hit	[firmicutes]	Staphylococcus hominis subsp. hominis C80 supercont1.9, who
. Staphylococcus simulans ACS-120-V-Sch1	108	1 hit	[firmicutes]	Staphylococcus simulans ACS-120-V-Sch1 supercont1.1, whole
. Staphylococcus simulans UMC-CNS-990	108	1 hit	[firmicutes]	Staphylococcus simulans UMC-CNS-990 SSIM990Contig01, whole
. Sulfitobacter sp. 20_GPM-1509m	108	1 hit	[a-proteobacteria]	Sulfitobacter sp. 20_GPM-1509m N517DRAFT_scaffold00005.5_C,
. Desulfobacterium autotrophicum HRM2	107	1 hit	[d-proteobacteria]	Desulfobacterium autotrophicum HRM2 chromosome, complete ge
. Desulfatirhabdium butyrativorans DSM 18734	107	1 hit	[d-proteobacteria]	Desulfatirhabdium butyrativorans DSM 18734 G492DRAFT_scaffo
. Rhodocyclaceae bacterium RZ94	106	1 hit	[b-proteobacteria]	Rhodocyclaceae bacterium RZ94 B594DRAFT_scaffold1.1, whole
. Methyloversatilis sp. FAM1	106	1 hit	[b-proteobacteria]	Methyloversatilis sp. FAM1 MetFAM1DRAFT_chromosome1.1_C, wh
. Methyloversatilis sp. RZ18-153	105	1 hit	[b-proteobacteria]	Methyloversatilis sp. RZ18-153 MetRZ18153DRAFT_Scaffold1.1_
. Staphylococcus sp. AL1	105	3 hits	[firmicutes]	Staphylococcus sp. AL1 contig16, whole genome shotgun seque
. Staphylococcus agnetis	105	1 hit	[firmicutes]	Staphylococcus agnetis strain CBMRN20813338 SAGN_20813338Co
. Syntrophobacter fumaroxidans MPOB	105	1 hit	[d-proteobacteria]	Syntrophobacter fumaroxidans MPOB chromosome, complete geno
. Staphylococcus caprae M23864:W1	105	2 hits	[firmicutes]	Staphylococcus caprae M23864:W1 SCAFFOLD1, whole genome sho
. Alcanivorax pacificus W11-5	104	1 hit	[g-proteobacteria]	Title was not found
. Nocardiopsis salina YIM 90010	104	1 hit	[high GC Gram+]	Nocardiopsis salina YIM 90010 contig_5, whole genome shotgu
. Agrobacterium rhizogenes	103	2 hits	[a-proteobacteria]	Agrobacterium rhizogenes strain YR147 EX05DRAFT_scaffold000
. Oceanobacillus kimchii X50	103	1 hit	[firmicutes]	Oceanobacillus kimchii X50 chromosome, whole genome shotgun
. Streptomyces sp. NRRL WC-3773	102	2 hits	[high GC Gram+]	Streptomyces sp. NRRL WC-3773 contig53.1, whole genome shot
. Staphylococcus saprophyticus subsp. saprophyticus KACC 16562 .	102	3 hits	[firmicutes]	Staphylococcus saprophyticus subsp. saprophyticus KACC 1656
. Sulfitobacter sp. CB2047	101	1 hit	[a-proteobacteria]	Sulfitobacter sp. CB2047 contig_3, whole genome shotgun seq
. Staphylococcus saprophyticus subsp. saprophyticus ATCC 15305 .	100	1 hit	[firmicutes]	Staphylococcus saprophyticus subsp. saprophyticus ATCC 1530
. Sulfitobacter sp. EE-36	100	1 hit	[a-proteobacteria]	Sulfitobacter sp. EE-36 scf_1099451318008, whole genome sho
. Desulfosporosinus orientis DSM 765	100	1 hit	[firmicutes]	Desulfosporosinus orientis DSM 765 chromosome, complete gen
. Bacillus sp. FJAT-13831	98	2 hits	[firmicutes]	Bacillus sp. FJAT-13831 Scaffold19, whole genome shotgun se
. Staphylococcus sp. E463	98	2 hits	[firmicutes]	Staphylococcus sp. E463 contig00062, whole genome shotgun s
. Thermoactinomyces sp. Gus2-1	98	1 hit	[firmicutes]	Thermoactinomyces sp. Gus2-1 c38, whole genome shotgun sequ
. Dactylosporangium aurantiacum	98	1 hit	[high GC Gram+]	Dactylosporangium aurantiacum strain NRRL B-8018 contig2.1,
. Sulfitobacter pontiacus 3SOLIMAR09	98	1 hit	[a-proteobacteria]	Sulfitobacter pontiacus 3SOLIMAR09 PM01_001, whole genome s
. Acinetobacter baumannii EGD-HP18	93	1 hit	[g-proteobacteria]	Acinetobacter baumannii EGD-HP18 contig104, whole genome sh
. Rhizobium tropici CIAT 899	98	1 hit	[a-proteobacteria]	Rhizobium tropici CIAT 899, complete genome
. Lentzea albidocapillata	97	1 hit	[high GC Gram+]	Lentzea albidocapillata strain NRRL B-24057 contig3.1, whol
. Methyloversatilis universalis EHg5	97	1 hit	[b-proteobacteria]	Methyloversatilis universalis EHg5 Metunv2DRAFT_Scaffold1.1
. Bradyrhizobium elkanii WSM2783	97	1 hit	[a-proteobacteria]	Bradyrhizobium elkanii WSM2783 YY7DRAFT_scaffold_54.55_C, w
. Intestinibacter bartlettii DORA_8_9	95	1 hit	[firmicutes]	Intestinibacter bartlettii DORA_8_9 Q606_CBAC00181, whole g
. Alkalilimnicola ehrlichii MLHE-1	96	1 hit	[g-proteobacteria]	Alkalilimnicola ehrlichii MLHE-1 chromosome, complete genome
. Sulfitobacter sp. NAS-14.1	96	1 hit	[a-proteobacteria]	Sulfitobacter sp. NAS-14.1 scf_1099451320477, whole genome

. Mesorhizobium sp. WSM3224	96	1 hit	[a-proteobacteria]	Mesorhizobium sp. WSM3224 YU3DRAFT_scaffold_0.1_C, whole ge
. Thauera sp. MZ1T	96	1 hit	[b-proteobacteria]	Thauera sp. MZ1T chromosome, complete genome
. Desulfosporosinus youngiae DSM 17734	95	1 hit	[firmicutes]	Desulfosporosinus youngiae DSM 17734 chromosome, whole geno
. Methylotenera sp. 1P/1	95	1 hit	[b-proteobacteria]	Methylotenera sp. 1P/1 A3Q3DRAFT_chromosome1.1_C, whole gen
. Pseudomonas fluorescens F113	95	1 hit	[g-proteobacteria]	Pseudomonas fluorescens F113 chromosome, complete genome
. Streptomyces sp. AW19M42	95	1 hit	[high GC Gram+]	Streptomyces sp. AW19M42, whole genome shotgun sequence
. Methyloversatilis sp. NVD	95	1 hit	[b-proteobacteria]	Methyloversatilis sp. NVD A3Q7DRAFT_scaffold1.1, whole geno
. Intestinibacter bartlettii DSM 16795	95	1 hit	[firmicutes]	Intestinibacter bartlettii DSM 16795 Scfld_02_14, whole gen
. Desulfospira joergensenii DSM 10085	95	1 hit	[d-proteobacteria]	Desulfospira joergensenii DSM 10085 F608DRAFT_scaffold00001
. Methylocystis sp. ATCC 49242	95	1 hit	[a-proteobacteria]	Methylocystis sp. ATCC 49242 strain Rockwell Met49242_MAH.6
. Pseudomonas aeruginosa	95	1 hit	[g-proteobacteria]	Pseudomonas aeruginosa strain AZPAE14505 AZPAE14505_contig_
. Desulfotomaculum gibsoniae DSM 7213	94	1 hit	[firmicutes]	Desulfotomaculum gibsoniae DSM 7213, complete genome
. Mesorhizobium sp. LNJC384A00	94	1 hit	[a-proteobacteria]	Mesorhizobium sp. LNJC384A00 scaffold0020, whole genome sho
. Sulfitobacter sp. NB-68	94	1 hit	[a-proteobacteria]	Sulfitobacter sp. NB-68 NB68_contig2, whole genome shotgun
. Spirochaeta sp. JC202	93	1 hit	[spirochetes]	Spirochaeta sp. JC202, whole genome shotgun sequence
. Streptosporangium roseum	93	1 hit	[high GC Gram+]	Streptosporangium roseum strain NRRL B-2638 contig42.1, who
. Streptacidiphilus oryzae TH49	93	1 hit	[high GC Gram+]	Streptacidiphilus oryzae TH49 BS73DRAFT_unitig_14_quiver.5_
. Streptomyces sp. NRRL F-6131	93	1 hit	[high GC Gram+]	Streptomyces sp. NRRL F-6131 contig51.1, whole genome shotg
. Streptomyces viridochromogenes Tue57	92	1 hit	[high GC Gram+]	Streptomyces viridochromogenes Tue57 Seq52, whole genome sh
. Comamonas badia DSM 17552	92	1 hit	[b-proteobacteria]	Comamonas badia DSM 17552 K320DRAFT_scaffold00001.1_C, whol
. Rhizobium sp. JGI 001013-F22	90	1 hit	[a-proteobacteria]	Rhizobium sp. JGI 001013-F22 C601DRAFT_contig_44.45_C, whol
. Methylocystis parvus OBBP	90	1 hit	[a-proteobacteria]	Methylocystis parvus OBBP contig010, whole genome shotgun s
. Mesorhizobium australicum WSM2073	90	1 hit	[a-proteobacteria]	Mesorhizobium australicum WSM2073, complete genome
. Streptomyces sp. CNS654	90	1 hit	[high GC Gram+]	Streptomyces sp. CNS654 CD02DRAFT_scaffold00035.35_C, whole
. Saccharopolyspora erythraea D	89	1 hit	[high GC Gram+]	Saccharopolyspora erythraea D scaffold00092, whole genome s
. Pseudomonas aeruginosa JD325	89	1 hit	[g-proteobacteria]	Pseudomonas aeruginosa JD325, whole genome shotgun sequence
. Saccharopolyspora erythraea NRRL 2338	89	2 hits	[high GC Gram+]	Saccharopolyspora erythraea NRRL 2338 ctg189, whole genome
. Desulfotomaculum ruminis DSM 2154	90	1 hit	[firmicutes]	Desulfotomaculum ruminis DSM 2154 chromosome, complete geno
. Burkholderia phenoliruptrix AC1100	89	1 hit	[b-proteobacteria]	Burkholderia phenoliruptrix AC1100 contig125, whole genome
. Pantholops hodgsonii	89	1 hit	[even-toed ungulates]	Pantholops hodgsonii unplaced genomic scaffold, PHO1.0 Scaf
. Mesorhizobium sp. LSJC255A00	89	1 hit	[a-proteobacteria]	Mesorhizobium sp. LSJC255A00 scaffold0003, whole genome sho
. Mesorhizobium sp. LSHC414A00	89	1 hit	[a-proteobacteria]	Mesorhizobium sp. LSHC414A00 scaffold0002, whole genome sho
. Mesorhizobium loti USDA 3471	89	1 hit	[a-proteobacteria]	Mesorhizobium loti USDA 3471 A3AUDRAFT_scaffold_12.13_C, wh
. Agrobacterium radiobacter K84	88	2 hits	[a-proteobacteria]	Agrobacterium radiobacter K84 chromosome 1, complete sequen
. Thermoactinomyces daqus	88	1 hit	[firmicutes]	Thermoactinomyces daqus strain H-18 H_18_contig_12, whole g
. Streptomyces purpeofuscus	88	1 hit	[high GC Gram+]	Streptomyces purpeofuscus strain NRRL B-1817 contig12.1, wh
. Rhizobium sp. YR060	87	1 hit	[a-proteobacteria]	Rhizobium sp. YR060 EX03DRAFT_scaffold00002.2_C, whole geno
. Pectobacterium sp. SCC3193	87	1 hit	[enterobacteria]	Pectobacterium sp. SCC3193, complete genome
. Actinoplanes friuliensis DSM 7358	87	1 hit	[high GC Gram+]	Actinoplanes friuliensis DSM 7358, complete genome
. Streptomyces sp. NRRL B-5680	86	1 hit	[high GC Gram+]	Streptomyces sp. NRRL B-5680 contig4.1, whole genome shotgu
. Streptomyces sp. e14	86	1 hit	[high GC Gram+]	Streptomyces sp. e14 genomic scaffold supercont1.2, whole g
. Streptomyces sp. Amel2xE9	86	1 hit	[high GC Gram+]	Streptomyces sp. Amel2xE9 B065DRAFT_scaffold_37.38, whole g
. Streptomyces aureofaciens	86	2 hits	[high GC Gram+]	Streptomyces aureofaciens strain NRRL B-2657 contig14.1, wh
. Streptomyces avellaneus	86	1 hit	[high GC Gram+]	Streptomyces avellaneus strain NRRL B-3447 contig1.1, whole
. Desulfatiglans anilini DSM 4660	86	1 hit	[d-proteobacteria]	Desulfatiglans anilini DSM 4660 H567DRAFT_scaffold00001.1_C
. Desulfitobacterium hafniense TCP-A	85	1 hit	[firmicutes]	Desulfitobacterium hafniense TCP-A DeshafDRAFT_Scaffold2.2,
. Desulfitobacterium hafniense DP7	85	1 hit	[firmicutes]	Desulfitobacterium hafniense DP7 Scfld3, whole genome shotg
. Desulfitobacterium hafniense PCP-1	85	1 hit	[firmicutes]	Desulfitobacterium hafniense PCP-1 A37YDRAFT_scaffold_20.21
. Desulfitobacterium hafniense DCB-2	85	1 hit	[firmicutes]	Desulfitobacterium hafniense DCB-2 chromosome, complete gen
. Halomonas elongata DSM 2581	85	1 hit	[g-proteobacteria]	Halomonas elongata DSM 2581 chromosome, complete genome
. Pseudomonas sp. EGD-AK9	81	1 hit	[g-proteobacteria]	Pseudomonas sp. EGD-AK9 Contig_741, whole genome shotgun se
. Streptomyces sp. NRRL S-244	85	1 hit	[high GC Gram+]	Streptomyces sp. NRRL S-244 contig25.1, whole genome shotgu
. Desulfitobacterium hafniense Y51	84	1 hit	[firmicutes]	Desulfitobacterium hafniense Y51 chromosome, complete genome
. Mesorhizobium sp. LNJC386A00	84	1 hit	[a-proteobacteria]	Mesorhizobium sp. LNJC386A00 scaffold0012, whole genome sho
. Streptomyces sp. NRRL F-5702	84	1 hit	[high GC Gram+]	Streptomyces sp. NRRL F-5702 contig73.1, whole genome shotg
. Streptomyces purpeochromogenes	84	2 hits	[high GC Gram+]	Streptomyces purpeochromogenes strain NRRL B-3012 contig41.
. Streptomyces californicus	84	4 hits	[high GC Gram+]	Streptomyces californicus strain NRRL B-3320 P050_Doro1_sca

. Streptomyces sp. NRRL F-3273	84	1 hit	[high GC Gram+]	Streptomyces sp. NRRL F-3273 contig18.1, whole genome shotg
. Streptomyces brasiliensis	84	1 hit	[high GC Gram+]	Streptomyces brasiliensis strain NRRL B-1626 contig10.1, wh
. Streptomyces sp. NRRL F-3218	84	1 hit	[high GC Gram+]	Streptomyces sp. NRRL F-3218 contig8.1, whole genome shotgu
. Streptomyces griseus subsp. rhodochrous	84	4 hits	[high GC Gram+]	Streptomyces griseus subsp. rhodochrous strain NRRL B-2932
. Streptomyces griseus subsp. griseus	83	2 hits	[high GC Gram+]	Streptomyces griseus subsp. griseus strain NRRL F-2227 cont
. Streptomyces puniceus	83	2 hits	[high GC Gram+]	Streptomyces puniceus strain NRRL B-2895 contig20.1, whole
. Streptomyces sp. NRRL WC-3540	83	1 hit	[high GC Gram+]	Streptomyces sp. NRRL WC-3540 contig14.1, whole genome shot
. Streptomyces sp. SolWspMP-sol2th	83	1 hit	[high GC Gram+]	Streptomyces sp. SolWspMP-sol2th B083DRAFT_scaffold_14.15_C
. Desulfitobacterium sp. PCE1	83	1 hit	[firmicutes]	Desulfitobacterium sp. PCE1 DesPCE1DRAFT_Scaffold1.1, whole
. Streptomyces sp. NRRL F-2202	83	1 hit	[high GC Gram+]	Streptomyces sp. NRRL F-2202 contig16.1, whole genome shotg
. Streptomyces sp. NRRL F-5681	83	1 hit	[high GC Gram+]	Streptomyces sp. NRRL F-5681 contig16.1, whole genome shotg
. Methylibium sp. T29-B	83	1 hit	[b-proteobacteria]	Methylibium sp. T29-B contig000056, whole genome shotgun se
. Methylibium sp. T29	83	1 hit	[b-proteobacteria]	Methylibium sp. T29 contig000034, whole genome shotgun sequ
. Dasania marina DSM 21967	83	1 hit	[g-proteobacteria]	Dasania marina DSM 21967 B067DRAFT_scaffold_6.7, whole geno
. Streptomyces sp. ScaeMP-e10	83	1 hit	[high GC Gram+]	Streptomyces sp. ScaeMP-e10 B061DRAFT_scaffold_1.2, whole g
. Streptomyces sp. NRRL B-1381	82	1 hit	[high GC Gram+]	Streptomyces sp. NRRL B-1381 contig65.1, whole genome shotg
. Streptomyces floridae	82	1 hit	[high GC Gram+]	Streptomyces floridae strain NRRL 2423 contig25.1, whole ge
. Streptomyces sp. Wigar10	82	1 hit	[high GC Gram+]	Streptomyces sp. Wigar10 contig00191, whole genome shotgun
. Ochrobactrum anthropi 60a	82	1 hit	[a-proteobacteria]	Ochrobactrum anthropi 60a Contig22, whole genome shotgun se
. Streptomyces alboviridis	82	1 hit	[high GC Gram+]	Streptomyces alboviridis strain NRRL B-1579 contig7.1, whol
. Streptomyces fulvissimus DSM 40593	82	1 hit	[high GC Gram+]	Streptomyces fulvissimus DSM 40593 chromosome, complete gen
. Streptomyces sp. NRRL S-623	82	1 hit	[high GC Gram+]	Streptomyces sp. NRRL S-623 contig20.1, whole genome shotgu
. Streptomyces sp. SA3_actF	80	1 hit	[high GC Gram+]	Streptomyces sp. SA3_actF contig00693, whole genome shotgun
. Streptomyces cyaneofuscatus	81	1 hit	[high GC Gram+]	Streptomyces cyaneofuscatus strain NRRL B-2570 contig12.1,
. Curvibacter gracilis ATCC BAA-807	81	1 hit	[b-proteobacteria]	Curvibacter gracilis ATCC BAA-807 L880DRAFT_scaffold00018.1
. Streptomyces sp. W007	80	1 hit	[high GC Gram+]	Streptomyces sp. W007 contig00070, whole genome shotgun seq
. Streptomyces flavochromogenes	80	1 hit	[high GC Gram+]	Streptomyces flavochromogenes strain NRRL B-2684 contig29.1
. Mesorhizobium sp. LSJC285A00	80	1 hit	[a-proteobacteria]	Mesorhizobium sp. LSJC285A00 scaffold0014, whole genome sho
. Streptomyces sp. SA3_actG	80	1 hit	[high GC Gram+]	Streptomyces sp. SA3_actG contig00051, whole genome shotgun
. Streptomyces purpureus KA281	80	1 hit	[high GC Gram+]	Streptomyces purpureus KA281 StrpuDRAFT_scaffold1.1, whole
. Streptomyces sp. NRRL F-5135	80	1 hit	[high GC Gram+]	Streptomyces sp. NRRL F-5135 contig16.1, whole genome shotg
. Streptomyces sp. CcalMP-8W	80	1 hit	[high GC Gram+]	Streptomyces sp. CcalMP-8W B053DRAFT_scaffold_11.12, whole
. Streptomyces sp. JS01	80	1 hit	[high GC Gram+]	Streptomyces sp. JS01 contig3, whole genome shotgun sequence
. Mesorhizobium loti MAFF303099	80	1 hit	[a-proteobacteria]	Mesorhizobium loti MAFF303099 chromosome, complete genome
. Streptomyces sp. Tu6071	79	1 hit	[high GC Gram+]	Streptomyces sp. Tu6071 chromosome, whole genome shotgun se
. Streptomyces sp. PCS3-D2	79	1 hit	[high GC Gram+]	Streptomyces sp. PCS3-D2 scaffold00003, whole genome shotgu
. Streptomyces griseus subsp. griseus NBRC 13350	78	1 hit	[high GC Gram+]	Streptomyces griseus subsp. griseus NBRC 13350 chromosome,
. Streptomyces griseus XylebKG-1	78	1 hit	[high GC Gram+]	Streptomyces griseus XylebKG-1 genomic scaffold scaffold_1,
. Streptomyces sp. SPB78	50	1 hit	[high GC Gram+]	Streptomyces sp. SPB78 supercont1.1, whole genome shotgun s
. Streptomyces exfoliatus DSM 41693	77	1 hit	[high GC Gram+]	Streptomyces exfoliatus DSM 41693 Contig324, whole genome s
. Streptomyces thermolilacinus SPC6	77	1 hit	[high GC Gram+]	Streptomyces thermolilacinus SPC6 contig1_1, whole genome s
. Streptomyces somaliensis DSM 40738	76	1 hit	[high GC Gram+]	Streptomyces somaliensis DSM 40738 contig071, whole genome
. Streptomyces viridochromogenes DSM 40736	75	1 hit	[high GC Gram+]	Streptomyces viridochromogenes DSM 40736 supercont1.1, whol
. Uliginosibacterium gangwonense DSM 18521	75	1 hit	[b-proteobacteria]	Uliginosibacterium gangwonense DSM 18521 B145DRAFT_scaffold
. Lechevalieria aerocolonigenes	74	1 hit	[high GC Gram+]	Lechevalieria aerocolonigenes strain NRRL B-3298 contig4.1,
. Streptomyces rimosus subsp. rimosus	73	19 hits	[high GC Gram+]	Streptomyces rimosus subsp. rimosus strain NRRL WC-3927 con
. Streptomyces sp. NRRL B-11253	72	1 hit	[high GC Gram+]	Streptomyces sp. NRRL B-11253 contig6.1, whole genome shotg
. Streptomyces peucetius	72	1 hit	[high GC Gram+]	Streptomyces peucetius strain NRRL WC-3868 contig5.1, whole
. Streptomyces sp. NRRL WC-3719	72	1 hit	[high GC Gram+]	Streptomyces sp. NRRL WC-3719 contig55.1, whole genome shot
. Streptomyces rimosus subsp. rimosus ATCC 10970	71	1 hit	[high GC Gram+]	Streptomyces rimosus subsp. rimosus ATCC 10970 contig00189,
. Streptomyces globisporus subsp. globisporus	71	1 hit	[high GC Gram+]	Streptomyces globisporus subsp. globisporus strain NRRL B-2
. Streptomyces rimosus	71	1 hit	[high GC Gram+]	Streptomyces rimosus strain R6-500MV9 contig022, whole geno
. Streptomyces sp. NRRL WC-3702	71	1 hit	[high GC Gram+]	Streptomyces sp. NRRL WC-3702 contig5.1, whole genome shotg
. Streptomyces capuensis	71	2 hits	[high GC Gram+]	Streptomyces capuensis strain NRRL B-3501 contig5.1, whole
. Streptomyces sp. NRRL WC-3703	71	1 hit	[high GC Gram+]	Streptomyces sp. NRRL WC-3703 contig7.1, whole genome shotg
. Streptomyces lavendulae subsp. lavendulae	71	2 hits	[high GC Gram+]	Streptomyces lavendulae subsp. lavendulae strain NRRL B-277
. Mastigocoleus testarum BC008	70	1 hit	[cyanobacteria]	Mastigocoleus testarum BC008 YY1DRAFT_scaffold_290.291_C, w

. Rhizobium sp. OK494	68	1 hit	[a-proteobacteria]	Rhizobium sp. OK494 EW96DRAFT_scaffold00009.9_C, whole geno
. Streptomyces monomycini	68	1 hit	[high GC Gram+]	Streptomyces monomycini strain NRRL B-24309 P063_Doro1_scaf
. Streptomyces wedmorensis	66	1 hit	[high GC Gram+]	Streptomyces wedmorensis strain NRRL 3426 contig40.1, whole
. Streptomyces sp. SS	65	1 hit	[high GC Gram+]	Streptomyces sp. SS scaffold2, whole genome shotgun sequence
. Streptomyces exfoliatus	65	2 hits	[high GC Gram+]	Streptomyces exfoliatus strain NRRL B-2924 contig10.1, whol
. Streptomyces sp. NRRL F-5727	65	1 hit	[high GC Gram+]	Streptomyces sp. NRRL F-5727 contig53.1, whole genome shotg
. Ricinus communis	64	1 hit	[eudicots]	Ricinus communis scf_1106159305140, whole genome shotgun se
. Streptomyces sclerotialus	64	1 hit	[high GC Gram+]	Streptomyces sclerotialus strain NRRL B-2317 contig43.1, wh
. Halomonas sp. A3H3	62	2 hits	[g-proteobacteria]	Halomonas sp. A3H3 genomic scaffold, HALA3H3_3393_scaffold3
. Streptomyces sp. CNS615	62	1 hit	[high GC Gram+]	Streptomyces sp. CNS615 D583DRAFT_scaffold00022.22, whole g
. Streptomyces griseoluteus	62	1 hit	[high GC Gram+]	Streptomyces griseoluteus strain NRRL ISP-5360 contig40.1,
. Leptolyngbya boryana PCC 6306	62	1 hit	[cyanobacteria]	Leptolyngbya boryana PCC 6306 LepboDRAFT_LPC.2, whole genom
. Streptomyces sp. CNR698	61	1 hit	[high GC Gram+]	Streptomyces sp. CNR698 B101DRAFT_scaffold_1.2_C, whole gen
. Streptomyces sp. CNT302	60	1 hit	[high GC Gram+]	Streptomyces sp. CNT302 D329DRAFT_scaffold00005.5, whole ge
. Sinorhizobium fredii HH103	60	1 hit	[a-proteobacteria]	Sinorhizobium fredii HH103, complete genome
. Rhizobium sp. PDO1-076	58	1 hit	[a-proteobacteria]	Rhizobium sp. PDO1-076 strain PDO-076 ctg220, whole genome
. Pelobacter seleniigenes DSM 18267	55	1 hit	[d-proteobacteria]	Pelobacter seleniigenes DSM 18267 N909DRAFT_scf718000000002
. Rhodococcus opacus B4	54	1 hit	[high GC Gram+]	Rhodococcus opacus B4, complete genome
. marine actinobacterium MedAcidi-G1	54	1 hit	[high GC Gram+]	Marine actinobacterium MedAcidi-G1 MedDCM-JULCOM-C169, whol
. Streptomyces sp. NRRL WC-3725	50	1 hit	[high GC Gram+]	Streptomyces sp. NRRL WC-3725 contig776.1, whole genome sho
. Candidatus Entotheonella sp. TSY1	52	1 hit	[d-proteobacteria]	Candidatus Entotheonella sp. TSY1 TSY1_scaffold00403, whole
. Mycobacterium tuberculosis	52	3 hits	[high GC Gram+]	Mycobacterium tuberculosis strain VRFCWCF MDRTB 17 Contig_4
. Mycobacterium tuberculosis M1906	52	1 hit	[high GC Gram+]	Mycobacterium tuberculosis M1906 adOYD-supercont1.9, whole
. Mycobacterium tuberculosis H2760	52	1 hit	[high GC Gram+]	Mycobacterium tuberculosis H2760 adPaX-supercont1.40, whole
. Mycobacterium tuberculosis H3005	52	1 hit	[high GC Gram+]	Mycobacterium tuberculosis H3005 adPcv-supercont1.39, whole
. Mycobacterium tuberculosis H3361	52	1 hit	[high GC Gram+]	Mycobacterium tuberculosis H3361 adOVm-supercont1.42, whole
. Mycobacterium tuberculosis NA-A0009	52	1 hit	[high GC Gram+]	Mycobacterium tuberculosis NA-A0009 contig060, whole genome
. Mycobacterium tuberculosis NA-A0008	52	1 hit	[high GC Gram+]	Mycobacterium tuberculosis NA-A0008 contig020, whole genome
. Mycobacterium tuberculosis M1893	52	1 hit	[high GC Gram+]	Mycobacterium tuberculosis M1893 adPda-supercont1.15, whole
. Mycobacterium tuberculosis M992	52	1 hit	[high GC Gram+]	Mycobacterium tuberculosis M992 adPeV-supercont1.16, whole
. Mycobacterium tuberculosis 43-16836	52	1 hit	[high GC Gram+]	Mycobacterium tuberculosis 43-16836 contig41, whole genome
. Mycobacterium tuberculosis M1961	52	1 hit	[high GC Gram+]	Mycobacterium tuberculosis M1961 adOUE-supercont1.13, whole
. Mycobacterium tuberculosis PR05	52	1 hit	[high GC Gram+]	Mycobacterium tuberculosis PR05 PR05_contig_13, whole genom
. Mycobacterium tuberculosis M1029	52	1 hit	[high GC Gram+]	Mycobacterium tuberculosis M1029 adOZr-supercont1.12, whole
. Mycobacterium tuberculosis H3367	52	1 hit	[high GC Gram+]	Mycobacterium tuberculosis H3367 adOYn-supercont1.10, whole
. Mycobacterium tuberculosis TKK_04_0085	52	1 hit	[high GC Gram+]	Mycobacterium tuberculosis TKK_04_0085 adWvH-supercont1.11,
. Mycobacterium tuberculosis M1221	52	1 hit	[high GC Gram+]	Mycobacterium tuberculosis M1221 adPdn-supercont1.13, whole
. Mycobacterium tuberculosis M1948	52	1 hit	[high GC Gram+]	Mycobacterium tuberculosis M1948 adOYA-supercont1.12, whole
. Mycobacterium tuberculosis H2463	52	1 hit	[high GC Gram+]	Mycobacterium tuberculosis H2463 adPcI-supercont1.11, whole
. Mycobacterium tuberculosis M13	52	1 hit	[high GC Gram+]	Mycobacterium tuberculosis M13 adOZj-supercont1.14, whole g
. Mycobacterium tuberculosis GM 1503	52	1 hit	[high GC Gram+]	Mycobacterium tuberculosis GM 1503 adCAK-supercont1.13, who
. Mycobacterium tuberculosis M2138	52	1 hit	[high GC Gram+]	Mycobacterium tuberculosis M2138 adPcW-supercont1.11, whole
. Mycobacterium tuberculosis M1429	52	1 hit	[high GC Gram+]	Mycobacterium tuberculosis M1429 adPdv-supercont1.13, whole
. Mycobacterium tuberculosis H3986	52	1 hit	[high GC Gram+]	Mycobacterium tuberculosis H3986 adPel-supercont1.10, whole
. Mycobacterium tuberculosis M1233	52	1 hit	[high GC Gram+]	Mycobacterium tuberculosis M1233 adPcT-supercont1.10, whole
. Mycobacterium tuberculosis M1560	52	1 hit	[high GC Gram+]	Mycobacterium tuberculosis M1560 adOZD-supercont1.9, whole
. Mycobacterium tuberculosis M1703	52	1 hit	[high GC Gram+]	Mycobacterium tuberculosis M1703 adOVL-supercont1.6, whole
. Mycobacterium tuberculosis M1928	52	1 hit	[high GC Gram+]	Mycobacterium tuberculosis M1928 adOYM-supercont1.6, whole
. Mycobacterium tuberculosis T17	52	1 hit	[high GC Gram+]	Mycobacterium tuberculosis T17 adCAF-supercont1.8, whole ge
. Mycobacterium tuberculosis M2134	52	1 hit	[high GC Gram+]	Mycobacterium tuberculosis M2134 adPgv-supercont1.8, whole
. Mycobacterium tuberculosis M2116	52	1 hit	[high GC Gram+]	Mycobacterium tuberculosis M2116 adOWK-supercont1.9, whole
. Mycobacterium tuberculosis M2129	52	1 hit	[high GC Gram+]	Mycobacterium tuberculosis M2129 adPfr-supercont1.10, whole
. Mycobacterium tuberculosis M1976	52	1 hit	[high GC Gram+]	Mycobacterium tuberculosis M1976 adPbH-supercont1.7, whole
. Mycobacterium tuberculosis M1011	52	1 hit	[high GC Gram+]	Mycobacterium tuberculosis M1011 adPfg-supercont1.8, whole
. Mycobacterium tuberculosis M2278	52	1 hit	[high GC Gram+]	Mycobacterium tuberculosis M2278 adPer-supercont1.6, whole
. Mycobacterium tuberculosis M2144	52	1 hit	[high GC Gram+]	Mycobacterium tuberculosis M2144 adOYY-supercont1.8, whole
. Mycobacterium tuberculosis M1979	52	1 hit	[high GC Gram+]	Mycobacterium tuberculosis M1979 adPcQ-supercont1.6, whole

. Mycobacterium tuberculosis M2343	52	1 hit	[high GC Gram+]	Mycobacterium tuberculosis M2343 adPbV-supercont1.6, whole
. Mycobacterium tuberculosis M1734	52	1 hit	[high GC Gram+]	Mycobacterium tuberculosis M1734 adPgC-supercont1.7, whole
. Mycobacterium tuberculosis M1025	52	1 hit	[high GC Gram+]	Mycobacterium tuberculosis M1025 adPae-supercont1.6, whole
. Mycobacterium tuberculosis H1578	52	1 hit	[high GC Gram+]	Mycobacterium tuberculosis H1578 adOVt-supercont1.7, whole
. Mycobacterium tuberculosis M2142	52	1 hit	[high GC Gram+]	Mycobacterium tuberculosis M2142 adOYv-supercont1.6, whole
. Mycobacterium tuberculosis M2416	52	1 hit	[high GC Gram+]	Mycobacterium tuberculosis M2416 adPbY-supercont1.5, whole
. Mycobacterium tuberculosis M2141	52	1 hit	[high GC Gram+]	Mycobacterium tuberculosis M2141 adPbj-supercont1.6, whole
. Mycobacterium tuberculosis M2128	52	1 hit	[high GC Gram+]	Mycobacterium tuberculosis M2128 adPcx-supercont1.5, whole
. Mycobacterium tuberculosis M2192	52	1 hit	[high GC Gram+]	Mycobacterium tuberculosis M2192 adOWF-supercont1.7, whole
. Mycobacterium tuberculosis M2003	52	1 hit	[high GC Gram+]	Mycobacterium tuberculosis M2003 adPaH-supercont1.5, whole
. Mycobacterium tuberculosis M1967	52	1 hit	[high GC Gram+]	Mycobacterium tuberculosis M1967 adPcd-supercont1.6, whole
. Mycobacterium tuberculosis M2021	52	1 hit	[high GC Gram+]	Mycobacterium tuberculosis M2021 adPfj-supercont1.7, whole
. Mycobacterium tuberculosis M2032	52	1 hit	[high GC Gram+]	Mycobacterium tuberculosis M2032 adPap-supercont1.6, whole
. Mycobacterium tuberculosis M2435	52	1 hit	[high GC Gram+]	Mycobacterium tuberculosis M2435 adPcD-supercont1.7, whole
. Mycobacterium tuberculosis H2102	52	1 hit	[high GC Gram+]	Mycobacterium tuberculosis H2102 adPcB-supercont1.5, whole
. Mycobacterium tuberculosis H2754	52	1 hit	[high GC Gram+]	Mycobacterium tuberculosis H2754 adOUL-supercont1.6, whole
. Mycobacterium tuberculosis M1236	52	1 hit	[high GC Gram+]	Mycobacterium tuberculosis M1236 adOUI-supercont1.7, whole
. Mycobacterium tuberculosis M2136	52	1 hit	[high GC Gram+]	Mycobacterium tuberculosis M2136 adOWv-supercont1.6, whole
. Mycobacterium tuberculosis H2438	52	1 hit	[high GC Gram+]	Mycobacterium tuberculosis H2438 adPeI-supercont1.7, whole
. Mycobacterium tuberculosis M1978	52	1 hit	[high GC Gram+]	Mycobacterium tuberculosis M1978 adPce-supercont1.5, whole
. Mycobacterium tuberculosis M1929	52	1 hit	[high GC Gram+]	Mycobacterium tuberculosis M1929 adPcC-supercont1.6, whole
. Mycobacterium tuberculosis H2264	52	1 hit	[high GC Gram+]	Mycobacterium tuberculosis H2264 adOVz-supercont1.5, whole
. Mycobacterium tuberculosis M2442	52	1 hit	[high GC Gram+]	Mycobacterium tuberculosis M2442 adPaq-supercont1.6, whole
. Mycobacterium tuberculosis MAL010134	52	1 hit	[high GC Gram+]	Mycobacterium tuberculosis MAL010134 adPgi-supercont1.4, wh
. Mycobacterium tuberculosis M2139	52	1 hit	[high GC Gram+]	Mycobacterium tuberculosis M2139 adOVd-supercont1.7, whole
. Mycobacterium tuberculosis H1580	52	1 hit	[high GC Gram+]	Mycobacterium tuberculosis H1580 adPch-supercont1.6, whole
. Mycobacterium tuberculosis M1726	52	1 hit	[high GC Gram+]	Mycobacterium tuberculosis M1726 adOXx-supercont1.7, whole
. Mycobacterium tuberculosis M2206	52	1 hit	[high GC Gram+]	Mycobacterium tuberculosis M2206 adPeU-supercont1.7, whole
. Mycobacterium tuberculosis M1384	52	1 hit	[high GC Gram+]	Mycobacterium tuberculosis M1384 adPfe-supercont1.5, whole
. Mycobacterium tuberculosis M2211	52	1 hit	[high GC Gram+]	Mycobacterium tuberculosis M2211 adPaF-supercont1.6, whole
. Mycobacterium tuberculosis M2346	52	1 hit	[high GC Gram+]	Mycobacterium tuberculosis M2346 adPcw-supercont1.4, whole
. Mycobacterium tuberculosis M1034	52	1 hit	[high GC Gram+]	Mycobacterium tuberculosis M1034 adPag-supercont1.9, whole
. Mycobacterium tuberculosis H3033	52	1 hit	[high GC Gram+]	Mycobacterium tuberculosis H3033 adPaW-supercont1.5, whole
. Mycobacterium tuberculosis M2140	52	1 hit	[high GC Gram+]	Mycobacterium tuberculosis M2140 adOWz-supercont1.6, whole
. Mycobacterium tuberculosis M2218	52	1 hit	[high GC Gram+]	Mycobacterium tuberculosis M2218 adOZI-supercont1.7, whole
. Mycobacterium tuberculosis M2137	52	1 hit	[high GC Gram+]	Mycobacterium tuberculosis M2137 adPgE-supercont1.6, whole
. Mycobacterium tuberculosis M2352	52	1 hit	[high GC Gram+]	Mycobacterium tuberculosis M2352 adPbb-supercont1.6, whole
. Mycobacterium tuberculosis M2010	52	1 hit	[high GC Gram+]	Mycobacterium tuberculosis M2010 adPaJ-supercont1.5, whole
. Mycobacterium tuberculosis M2145	52	1 hit	[high GC Gram+]	Mycobacterium tuberculosis M2145 adPcj-supercont1.7, whole
. Mycobacterium tuberculosis M1570	52	1 hit	[high GC Gram+]	Mycobacterium tuberculosis M1570 adPei-supercont1.8, whole
. Mycobacterium tuberculosis M1030	52	1 hit	[high GC Gram+]	Mycobacterium tuberculosis M1030 adPdw-supercont1.7, whole
. Mycobacterium tuberculosis M2203	52	1 hit	[high GC Gram+]	Mycobacterium tuberculosis M2203 adPfd-supercont1.7, whole
. Mycobacterium tuberculosis M2259	52	1 hit	[high GC Gram+]	Mycobacterium tuberculosis M2259 adPdO-supercont1.7, whole
. Mycobacterium tuberculosis H1589	52	1 hit	[high GC Gram+]	Mycobacterium tuberculosis H1589 adOXP-supercont1.7, whole
. Mycobacterium tuberculosis H3004	52	1 hit	[high GC Gram+]	Mycobacterium tuberculosis H3004 adPbA-supercont1.3, whole
. Mycobacterium tuberculosis H1249	52	1 hit	[high GC Gram+]	Mycobacterium tuberculosis H1249 adPey-supercont1.6, whole
. Mycobacterium tuberculosis M1816	52	1 hit	[high GC Gram+]	Mycobacterium tuberculosis M1816 adPdB-supercont1.5, whole
. Mycobacterium tuberculosis H3094	52	1 hit	[high GC Gram+]	Mycobacterium tuberculosis H3094 adOVs-supercont1.5, whole
. Mycobacterium tuberculosis M1959	52	1 hit	[high GC Gram+]	Mycobacterium tuberculosis M1959 adOVC-supercont1.8, whole
. Mycobacterium tuberculosis M2384	52	1 hit	[high GC Gram+]	Mycobacterium tuberculosis M2384 adOVQ-supercont1.3, whole
. Mycobacterium tuberculosis TKK_04_0120	52	1 hit	[high GC Gram+]	Mycobacterium tuberculosis TKK_04_0120 adYHm-supercont1.3,
. Mycobacterium tuberculosis H1996	52	1 hit	[high GC Gram+]	Mycobacterium tuberculosis H1996 adOYI-supercont1.2, whole
. Mycobacterium tuberculosis M2198	52	1 hit	[high GC Gram+]	Mycobacterium tuberculosis M2198 adPdQ-supercont1.2, whole
. Mycobacterium tuberculosis UT0046	52	1 hit	[high GC Gram+]	Mycobacterium tuberculosis UT0046 adDna-supercont1.4, whole
. Mycobacterium tuberculosis H2398	52	1 hit	[high GC Gram+]	Mycobacterium tuberculosis H2398 adOZz-supercont1.3, whole
. Mycobacterium tuberculosis T92	52	1 hit	[high GC Gram+]	Mycobacterium tuberculosis T92 supercont1.7 genomic scaffol
. Mycobacterium tuberculosis H2375	52	1 hit	[high GC Gram+]	Mycobacterium tuberculosis H2375 adPeS-supercont1.2, whole

Organism Report

Bacillus phage phiNIT1 [viruses] taxid 207656

ref|NC_021856.1| Bacillus phage phiNIT1 DNA, complete genome	406	4e-125

Bacillus phage Grass [viruses] taxid 1406785

ref|NC_022771.1| Bacillus phage Grass, complete genome	398	3e-122

Bacillus phage PM1 [viruses] taxid 547228

ref|NC_020883.1| Bacillus phage PM1 DNA, complete genome	192	9e-51

Bacillus phage Bobb [viruses] taxid 1527469

ref|NC_024792.1| Bacillus phage Bobb, complete genome	186	1e-48

Bacillus phage phiAGATE [viruses] taxid 1204533

ref|NC_020081.2| Bacillus phage phiAGATE, complete genome	182	2e-47

Bacillus sp. FJAT-14515 [firmicutes] taxid 1208599

ref|NZ_AYSD01000010.1| Bacillus sp. FJAT-14515 Scaffold2_1...	167	5e-42

Bacillus phage SPP1 [viruses] taxid 10724

ref|NC_004166.2| Bacillus phage SPP1, complete genome	162	1e-40

Bacillus sp. DW5-4 [firmicutes] taxid 1178540

ref|NZ_JOTP01000012.1| Bacillus sp. DW5-4 contig12, whole ...	152	4e-37
ref|NZ_JOTP01000025.1| Bacillus sp. DW5-4 contig25, whole ...	139	2e-32

Bacillus pumilus SAFR-032 [firmicutes] taxid 315750

ref|NC_009848.1| Bacillus pumilus SAFR-032 chromosome, com...	151	1e-36

Bacillus sp. 171095_106 [firmicutes] taxid 1286363

ref|NZ_AZVK01000004.1| Bacillus sp. 171095_106 H139DRAFT_s...	151	1e-36
ref|NZ_AZVK01000005.1| Bacillus sp. 171095_106 H139DRAFT_s...	125	1e-27
ref|NZ_KI911358.1| Bacillus sp. 171095_106 H139DRAFT_scaff...	112	3e-23

Bacillus xiamenensis [firmicutes] taxid 1178537

ref|NZ_AMSH01000031.1| Bacillus xiamenensis strain HYC-10 ...	151	1e-36
ref|NZ_AMSH01000053.1| Bacillus xiamenensis strain HYC-10 ...	137	9e-32

Bacillus megaterium WSH-002 [firmicutes] taxid 1006007

ref|NC_017138.1| Bacillus megaterium WSH-002 chromosome, c...	150	2e-36

Bacillus pumilus [firmicutes] taxid 1408

ref|NZ_JOJX01000006.1| Bacillus pumilus strain 3-19 contig...	150	2e-36
ref|NZ_JHUD01000003.1| Bacillus pumilus strain 7P contig00...	150	2e-36
ref|NZ_JFBY01000025.1| Bacillus pumilus strain Fairview co...	143	7e-34
ref|NZ_JOJX01000003.1| Bacillus pumilus strain 3-19 contig...	140	4e-33
ref|NZ_JFBY01000021.1| Bacillus pumilus strain Fairview co...	140	5e-33

Bacillus sp. JGI 001006-L10 [firmicutes] taxid 1167733

ref|NZ_AYGU01000001.1| Bacillus sp. JGI 001006-L10 C598DRA...	150	2e-36
ref|NZ_AYGU01000023.1| Bacillus sp. JGI 001006-L10 C598DRA...	82	1e-13

Bacillus megaterium DSM 319 [firmicutes] taxid 592022

ref|NC_014103.1| Bacillus megaterium DSM 319 chromosome, c...	150	3e-36

Bacillus megaterium NBRC 15308 = ATCC 14581 [firmicutes] taxid 1348623

ref|NZ_JJMH01000072.1| Bacillus megaterium NBRC 15308 = AT...	150	3e-36
ref|NZ_JJMH01000030.1| Bacillus megaterium NBRC 15308 = AT...	120	3e-26
ref|NZ_JJMH01000025.1| Bacillus megaterium NBRC 15308 = AT...	114	4e-24
ref|NZ_JJMH01000056.1| Bacillus megaterium NBRC 15308 = AT...	86	1e-14

Bacillus megaterium [firmicutes] taxid 1404

ref|NZ_KN125581.1| Bacillus megaterium strain BMS scaffold...	150	3e-36

Bacillus megaterium NCT-2 [firmicutes] taxid 1138452

ref|NZ_AHTF01000053.1| Bacillus megaterium NCT-2 contig53,...	149	3e-36
ref|NZ_AHTF01000065.1| Bacillus megaterium NCT-2 contig65,...	119	1e-25
ref|NZ_AHTF01000092.1| Bacillus megaterium NCT-2 contig92,...	113	8e-24
ref|NZ_AHTF01000068.1| Bacillus megaterium NCT-2 contig68,...	85	3e-14

Bacillus pumilus ATCC 7061 [firmicutes] taxid 536229

ref|NZ_ABRX01000001.1| Bacillus pumilus ATCC 7061 BAT.Cont...	149	4e-36

Bacillus pumilus S-1 [firmicutes] taxid 1076692

ref|NZ_JH137698.1| Bacillus pumilus S-1 scaffold52, whole ...	149	6e-36
ref|NZ_JH137788.1| Bacillus pumilus S-1 scaffold142, whole...	137	6e-32

Bacillus sp. Aph1 [firmicutes] taxid 1202456

ref|NZ_ANNS01000121.1| Bacillus sp. Aph1 Contig122, whole ...	148	8e-36
ref|NZ_ANNS01000350.1| Bacillus sp. Aph1 Contig354, whole ...	120	5e-26
ref|NZ_ANNS01000391.1| Bacillus sp. Aph1 Contig395, whole ...	111	3e-23
ref|NZ_ANNS01000215.1| Bacillus sp. Aph1 Contig218, whole ...	82	2e-13

Bacillus aryabhattai [firmicutes] taxid 412384

ref|NZ_JPIE01000001.1| Bacillus aryabhattai strain GZ03 co...	148	1e-35
ref|NZ_JPIE01000003.1| Bacillus aryabhattai strain GZ03 co...	111	5e-23
ref|NZ_JPIE01000005.1| Bacillus aryabhattai strain GZ03 co...	82	2e-13

Bacillus flexus 27Col1.1E [firmicutes] taxid 1292043

ref|NZ_AZUJ01000009.1| Bacillus flexus 27Col1.1E H543DRAFT...	148	2e-35
ref|NZ_AZUJ01000004.1| Bacillus flexus 27Col1.1E H543DRAFT...	117	5e-25
ref|NZ_AZUJ01000002.1| Bacillus flexus 27Col1.1E H543DRAFT...	114	3e-24

Bacillus sp. RP1137 [firmicutes] taxid 1380110

ref|NZ_AXZS01000012.1| Bacillus sp. RP1137 contig_10, whol...	147	2e-35

Bacillus megaterium QM B1551 [firmicutes] taxid 545693

ref|NC_014019.1| Bacillus megaterium QM B1551 chromosome, ...	147	2e-35

Bacillus altitudinis [firmicutes] taxid 293387

ref|NZ_JOVS01000013.1| Bacillus altitudinis strain B-388 c...	147	2e-35
ref|NZ_JOVS01000004.1| Bacillus altitudinis strain B-388 c...	137	6e-32

Bacillus pumilus INR7 [firmicutes] taxid 1423688

ref|NZ_AYTK01000044.1| Bacillus pumilus INR7 contig44, who...	147	2e-35
ref|NZ_AYTK01000049.1| Bacillus pumilus INR7 contig49, who...	137	6e-32

Bacillus pumilus BA06 [firmicutes] taxid 1227811

ref|NZ_AMDH01000009.1| Bacillus pumilus BA06 BA_1108, whol...	147	4e-35

```
 Bacillus altitudinis 41KF2b [firmicutes] taxid 1178544
ref|NZ_ASJC01000001.1| Bacillus altitudinis 41KF2b contig1...     147  4e-35

 Bacillus sp. UNC125MFCrub1.1 [firmicutes] taxid 1380371
ref|NZ_KL370769.1| Bacillus sp. UNC125MFCrub1.1 N521DRAFT_...     147  4e-35

 Bacillus sp. 278922_107 [firmicutes] taxid 1292019
ref|NZ_KI911354.1| Bacillus sp. 278922_107 H622DRAFT_scaff...     145  9e-35
ref|NZ_KI911355.1| Bacillus sp. 278922_107 H622DRAFT_scaff...     112  1e-23

 Bacillus safensis FO-36b [firmicutes] taxid 1178541
ref|NZ_ASJD01000003.1| Bacillus safensis FO-36b contig3, w...     144  3e-34

 Bacillus phage SP10 [viruses] taxid 941058
ref|NC_019487.1| Bacillus phage SP10, complete genome             144  3e-34

 Bacillus safensis VK [firmicutes] taxid 1355022
ref|NZ_AUPF01000009.1| Bacillus safensis VK contig00009, w...     144  3e-34
ref|NZ_AUPF01000004.1| Bacillus safensis VK contig00004, w...     140  5e-33

 Bacillus pumilus CCMA-560 [firmicutes] taxid 1350468
ref|NZ_AUYP01000028.1| Bacillus pumilus CCMA-560 NODE_36.3...     144  3e-34
ref|NZ_AUYP01000029.1| Bacillus pumilus CCMA-560 NODE_38.1...     140  5e-33

 Bacillus safensis [firmicutes] taxid 561879
ref|NZ_JNBO01000040.1| Bacillus safensis strain CFA06 cont...     144  3e-34
ref|NZ_JNBO01000042.1| Bacillus safensis strain CFA06 cont...     140  5e-33

 Bacillus firmus DS1 [firmicutes] taxid 1307436
ref|NZ_APVL01000012.1| Bacillus firmus DS1 scaffold12, who...     140  6e-33

 Bacillus licheniformis G-1 [firmicutes] taxid 1413213
ref|NZ_AZSK01000029.1| Bacillus licheniformis G-1 CONTIG_3...     138  3e-32
ref|NZ_AZSK01000031.1| Bacillus licheniformis G-1 CONTIG_3...     133  1e-30

 Bacillus sp. CPSM8 [firmicutes] taxid 1202457
ref|NZ_ANNR02000001.1| Bacillus sp. CPSM8 SM8.a5.scaffold1...     138  3e-32
ref|NZ_ANNR02000023.1| Bacillus sp. CPSM8 SM8.a5.scaffold3...     133  1e-30
ref|NZ_ANNR02000033.1| Bacillus sp. CPSM8 SM8.a5.scaffold6...     113  1e-23

 Bacillus sp. MSP5.4 [firmicutes] taxid 1071069
ref|NZ_JXAP01000003.1| Bacillus sp. MSP5.4 contig00003, wh...     138  3e-32
ref|NZ_JXAP01000001.1| Bacillus sp. MSP5.4 contig00001, wh...     133  1e-30
ref|NZ_JXAP01000002.1| Bacillus sp. MSP5.4 contig00002, wh...     113  1e-23

 Bacillus sp. SB47 [firmicutes] taxid 1071079
ref|NZ_KE819959.1| Bacillus sp. SB47 scaffold00001, whole ...     138  3e-32

 Bacillus licheniformis CG-B52 [firmicutes] taxid 1368424
ref|NZ_AVEZ01000033.1| Bacillus licheniformis CG-B52 scaff...     137  5e-32
ref|NZ_AVEZ01000007.1| Bacillus licheniformis CG-B52 scaff...     134  9e-31
ref|NZ_AVEZ01000035.1| Bacillus licheniformis CG-B52 scaff...     118  2e-25

 Bacillus licheniformis 10-1-A [firmicutes] taxid 1169411
ref|NZ_AJLV01000022.1| Bacillus licheniformis 10-1-A conti...     137  5e-32
ref|NZ_AJLV01000021.1| Bacillus licheniformis 10-1-A conti...     134  9e-31
ref|NZ_AJLV01000018.1| Bacillus licheniformis 10-1-A conti...     118  2e-25

 Bacillus licheniformis 5-2-D [firmicutes] taxid 1169412
ref|NZ_AJLW01000043.1| Bacillus licheniformis 5-2-D contig...     137  5e-32
ref|NZ_AJLW01000040.1| Bacillus licheniformis 5-2-D contig...     134  9e-31
ref|NZ_AJLW01000009.1| Bacillus licheniformis 5-2-D contig...     118  2e-25

 Bacillus licheniformis F2-1 [firmicutes] taxid 1433291
ref|NZ_AZSM01000035.1| Bacillus licheniformis F2-1 CONTIG_...     137  5e-32
ref|NZ_AZSM01000048.1| Bacillus licheniformis F2-1 CONTIG_...     134  9e-31

 Bacillus licheniformis F1-1 [firmicutes] taxid 1433290
ref|NZ_AZSL01000001.1| Bacillus licheniformis F1-1 CONTIG_...     137  5e-32
ref|NZ_AZSL01000008.1| Bacillus licheniformis F1-1 CONTIG_...     134  9e-31

 Bacillus licheniformis [firmicutes] taxid 1402
ref|NZ_JFYM01000001.1| Bacillus licheniformis strain 3F-3 ...     137  5e-32
ref|NZ_KN125579.1| Bacillus licheniformis strain 12759 sca...     137  6e-32
ref|NZ_JFYM01000037.1| Bacillus licheniformis strain 3F-3 ...     134  9e-31

 Bacillus licheniformis WX-02 [firmicutes] taxid 1126218
ref|NZ_JH636050.1| Bacillus licheniformis WX-02 MUY_1, who...     137  5e-32

 Bacillus licheniformis DSM 13 = ATCC 14580 [firmicutes] taxid 279010
ref|NC_006270.3| Bacillus licheniformis ATCC 14580 chromos...     137  5e-32
ref|NC_006322.1| Bacillus licheniformis DSM 13 = ATCC 1458...     137  5e-32

 Bacillus sp. BT1B_CT2 [firmicutes] taxid 665958
ref|NZ_GL635726.1| Bacillus sp. BT1B_CT2 supercont1.1, who...     137  6e-32

 Staphylococcus aureus subsp. aureus CO-98 [firmicutes] taxid 904802
ref|NZ_JHTU01000043.1| Staphylococcus aureus subsp. aureus...     136  1e-31
ref|NZ_JHTU01000087.1| Staphylococcus aureus subsp. aureus...     122  9e-27
ref|NZ_JHTU01000031.1| Staphylococcus aureus subsp. aureus...     104  1e-20
ref|NZ_JHTU01000078.1| Staphylococcus aureus subsp. aureus...      95  2e-17

 Bacillus licheniformis 9945A [firmicutes] taxid 766760
ref|NC_021362.1| Bacillus licheniformis 9945A, complete ge...     136  1e-31

 Bacillus cereus AH1272 [firmicutes] taxid 526993
ref|NZ_CM000740.1| Bacillus cereus AH1272 chromosome, whol...     135  3e-31

 Bacillus cereus AH1273 [firmicutes] taxid 526994
ref|NZ_CM000741.1| Bacillus cereus AH1273 chromosome, whol...     135  3e-31

 Bacillus atrophaeus UCMB-5137 [firmicutes] taxid 1239783
ref|NZ_CM001847.1| Bacillus atrophaeus UCMB-5137 chromosom...     135  3e-31

 Bacillus sp. NSP9.1 [firmicutes] taxid 1071078
ref|NZ_KE819964.1| Bacillus sp. NSP9.1 scaffold00001, whol...     135  4e-31
```

```
 Staphylococcus massiliensis CCUG 55927 [firmicutes] taxid 1189613
ref|NZ_JH815593.1| Staphylococcus massiliensis CCUG 55927 ...     135  4e-31

 Bacillus licheniformis CGMCC 3963 [firmicutes] taxid 1232672
ref|NZ_AMWQ01000005.1| Bacillus licheniformis CGMCC 3963 c...     134  6e-31
ref|NZ_AMWQ01000036.1| Bacillus licheniformis CGMCC 3963 c...     134  9e-31
ref|NZ_AMWQ01000032.1| Bacillus licheniformis CGMCC 3963 c...     118  2e-25

 Bacillus atrophaeus C89 [firmicutes] taxid 334727
ref|NZ_AJRJ01000079.1| Bacillus atrophaeus C89 contig00007...     134  1e-30
ref|NZ_AJRJ01000041.1| Bacillus atrophaeus C89 contig00004...     122  7e-27
ref|NZ_AJRJ01000085.1| Bacillus atrophaeus C89 contig00008...     121  2e-26

 Bacillus atrophaeus BACI051-N [firmicutes] taxid 876283
ref|NZ_AEFY01000012.1| Bacillus atrophaeus BACI051-N conti...     134  1e-30
ref|NZ_AEFY01000011.1| Bacillus atrophaeus BACI051-N conti...     122  7e-27
ref|NZ_AEFY01000013.1| Bacillus atrophaeus BACI051-N conti...     121  2e-26

 Bacillus atrophaeus BACI051-E [firmicutes] taxid 882455
ref|NZ_AEFX01000022.1| Bacillus atrophaeus BACI051-E conti...     134  1e-30
ref|NZ_AEFX01000010.1| Bacillus atrophaeus BACI051-E conti...     122  7e-27

 Bacillus atrophaeus 1013-2 [firmicutes] taxid 723889
ref|NZ_AEFT01000020.1| Bacillus atrophaeus 1013-2 contig00...     134  1e-30
ref|NZ_AEFT01000003.1| Bacillus atrophaeus 1013-2 contig00...     122  7e-27
ref|NZ_AEFT01000014.1| Bacillus atrophaeus 1013-2 contig00...     121  2e-26

 Bacillus atrophaeus ATCC 49822-1 [firmicutes] taxid 743710
ref|NZ_AEFV01000026.1| Bacillus atrophaeus ATCC 49822-1 co...     134  1e-30
ref|NZ_AEFV01000016.1| Bacillus atrophaeus ATCC 49822-1 co...     122  7e-27

 Bacillus atrophaeus ATCC 9372-2 [firmicutes] taxid 723892
ref|NZ_AEFU01000004.1| Bacillus atrophaeus ATCC 9372-2 con...     134  1e-30
ref|NZ_AEFU01000032.1| Bacillus atrophaeus ATCC 9372-2 con...     122  7e-27
ref|NZ_AEFU01000003.1| Bacillus atrophaeus ATCC 9372-2 con...     121  2e-26

 Bacillus atrophaeus ATCC 9372-1 [firmicutes] taxid 903520
ref|NZ_AEFM01000013.1| Bacillus atrophaeus ATCC 9372-1 con...     134  1e-30
ref|NZ_AEFM01000010.1| Bacillus atrophaeus ATCC 9372-1 con...     122  7e-27

 Bacillus atrophaeus Detrick-2 [firmicutes] taxid 720557
ref|NZ_AEFQ01000025.1| Bacillus atrophaeus Detrick-2 conti...     134  1e-30
ref|NZ_AEFQ01000012.1| Bacillus atrophaeus Detrick-2 conti...     122  7e-27
ref|NZ_AEFQ01000031.1| Bacillus atrophaeus Detrick-2 conti...     121  2e-26

 Bacillus atrophaeus ATCC 49822-2 [firmicutes] taxid 743711
ref|NZ_AEFW01000001.1| Bacillus atrophaeus ATCC 49822-2 co...     134  1e-30
ref|NZ_AEFW01000002.1| Bacillus atrophaeus ATCC 49822-2 co...     122  7e-27

 Bacillus atrophaeus Detrick-1 [firmicutes] taxid 720556
ref|NZ_AEFP01000026.1| Bacillus atrophaeus Detrick-1 conti...     134  1e-30
ref|NZ_AEFP01000007.1| Bacillus atrophaeus Detrick-1 conti...     122  7e-27

 Bacillus atrophaeus Detrick-3 [firmicutes] taxid 720558
ref|NZ_AEFR01000006.1| Bacillus atrophaeus Detrick-3 conti...     134  1e-30
ref|NZ_AEFR01000024.1| Bacillus atrophaeus Detrick-3 conti...     122  7e-27

 Bacillus atrophaeus 1013-1 [firmicutes] taxid 723888
ref|NZ_AEFS01000009.1| Bacillus atrophaeus 1013-1 contig00...     134  1e-30

 Bacillus subtilis subsp. niger [firmicutes] taxid 1495315
ref|NZ_KN049968.1| Bacillus subtilis subsp. niger strain P...     134  1e-30

 Bacillus atrophaeus 1942 [firmicutes] taxid 720555
ref|NC_014639.1| Bacillus atrophaeus 1942 chromosome, comp...     134  1e-30

 Bacillus atrophaeus subsp. globigii [firmicutes] taxid 1529886
ref|NZ_CP007640.1| Bacillus atrophaeus subsp. globigii str...     134  1e-30

 Staphylococcus epidermidis NIHLM021 [firmicutes] taxid 979212
ref|NZ_AKGV01000037.1| Staphylococcus epidermidis NIHLM021...     133  2e-30
ref|NZ_AKGV01000023.1| Staphylococcus epidermidis NIHLM021...     124  1e-27
ref|NZ_AKGV01000036.1| Staphylococcus epidermidis NIHLM021...     114  4e-24
ref|NZ_AKGV01000058.1| Staphylococcus epidermidis NIHLM021...     112  3e-23
ref|NZ_AKGV01000002.1| Staphylococcus epidermidis NIHLM021...     100  2e-19

 Bacillus sonorensis NBRC 101234 = KCTC 13918 [firmicutes] taxid 1220591
ref|NZ_AYTN01000052.1| Bacillus sonorensis NBRC 101234 = K...     132  5e-30
ref|NZ_AYTN01000014.1| Bacillus sonorensis NBRC 101234 = K...     128  7e-29
ref|NZ_AYTN01000001.1| Bacillus sonorensis NBRC 101234 = K...     122  1e-26

 Bacillus endophyticus 2102 [firmicutes] taxid 1196029
ref|NZ_ALIM01000034.1| Bacillus endophyticus 2102 contig52...     131  5e-30
ref|NZ_ALIM01000023.1| Bacillus endophyticus 2102 contig30...     122  7e-27

 Streptomyces roseochromogenus subsp. oscitans DS 12.976 [high GC Gram+]
taxid 1352936
ref|NZ_CM002285.1| Streptomyces roseochromogenus subsp. os...     131  6e-30

 Bacillus subtilis subsp. subtilis str. SC-8 [firmicutes] taxid 1089443
ref|NZ_AGFW01000009.1| Bacillus subtilis subsp. subtilis s...     129  6e-29
ref|NZ_AGFW01000004.1| Bacillus subtilis subsp. subtilis s...     124  2e-27
ref|NZ_AGFW01000008.1| Bacillus subtilis subsp. subtilis s...     100  2e-19

 Bacillus subtilis [firmicutes] taxid 1423
ref|NZ_JMEF01000089.1| Bacillus subtilis strain KATMIRA193...     127  1e-28
ref|NZ_JNCN01000034.1| Bacillus subtilis strain E72 scaffo...     127  2e-28
ref|NZ_JPYY01000008.1| Bacillus subtilis strain NKYL29 Sca...     124  2e-27
ref|NZ_JNCN01000035.1| Bacillus subtilis strain E72 scaffo...     124  3e-27
ref|NZ_JPNZ01000001.1| Bacillus subtilis strain GXA-28 Con...     124  3e-27
ref|NZ_KN049967.1| Bacillus subtilis strain BST scaffold1,...     123  5e-27
ref|NZ_JMEF01000012.1| Bacillus subtilis strain KATMIRA193...     122  6e-27
ref|NZ_JPYY01000002.1| Bacillus subtilis strain NKYL29 Sca...     122  1e-26
ref|NZ_JMEF01000056.1| Bacillus subtilis strain KATMIRA193...     103  2e-20
ref|NZ_JMEF01000023.1| Bacillus subtilis strain KATMIRA193...      75  4e-20

 Bacillus siamensis KCTC 13613 [firmicutes] taxid 1177185
ref|NZ_AJVF01000013.1| Bacillus siamensis KCTC 13613 conti...     127  1e-28
```

```
 Bacillus phage SPbeta [viruses] taxid 66797
ref|NC_001884.1| Bacillus phage SPBc2, complete genome           127  2e-28

 Bacillus subtilis MB73/2 [firmicutes] taxid 1267547
ref|NZ_AOTY01000004.1| Bacillus subtilis MB73/2 bacsub.con...     127  2e-28
ref|NZ_AOTY01000002.1| Bacillus subtilis MB73/2 bacsub.con...     124  3e-27

 Bacillus sp. EGD-AK10 [firmicutes] taxid 1386080
ref|NZ_AVPM01000001.1| Bacillus sp. EGD-AK10 contig1, whol...     127  2e-28

 Bacillus subtilis E1 [firmicutes] taxid 1290773
ref|NZ_CAUC01000006.1| Bacillus subtilis E1, whole genome ...     127  2e-28
ref|NZ_CAUC01000008.1| Bacillus subtilis E1, whole genome ...     103  3e-20

 Bacillus subtilis subsp. subtilis str. 168 [firmicutes] taxid 224308
ref|NZ_JNCM01000036.1| Bacillus subtilis subsp. subtilis s...     127  2e-28
ref|NZ_CM000487.1| Bacillus subtilis subsp. subtilis str. ...     127  2e-28
ref|NC_000964.3| Bacillus subtilis subsp. subtilis str. 16...     127  2e-28
ref|NZ_JNCM01000035.1| Bacillus subtilis subsp. subtilis s...     124  3e-27

 Bacillus subtilis QB928 [firmicutes] taxid 1220533
ref|NC_018520.1| Bacillus subtilis QB928 chromosome, compl...     127  2e-28

 Bacillus subtilis subsp. subtilis str. JH642 [firmicutes] taxid 535025
ref|NZ_CM000489.1| Bacillus subtilis subsp. subtilis str. ...     127  2e-28

 Bacillus subtilis subsp. subtilis str. NCIB 3610 [firmicutes] taxid 535026
ref|NZ_CM000488.1| Bacillus subtilis subsp. subtilis str. ...     127  2e-28

 Bacillus subtilis subsp. subtilis str. SMY [firmicutes] taxid 535024
ref|NZ_CM000490.1| Bacillus subtilis subsp. subtilis str. ...     127  2e-28

 Bacillus subtilis subsp. subtilis 6051-HGW [firmicutes] taxid 1147161
ref|NC_020507.1| Bacillus subtilis subsp. subtilis 6051-HG...     127  2e-28

 Bacillus subtilis PS216 [firmicutes] taxid 1315975
ref|NZ_AQGR01000090.1| Bacillus subtilis PS216 ctg71800000...     124  2e-28
ref|NZ_AQGR01000044.1| Bacillus subtilis PS216 ctg71800000...     124  3e-27
ref|NZ_AQGR01000124.1| Bacillus subtilis PS216 ctg71800000...     122  6e-27
ref|NZ_AQGR01000039.1| Bacillus subtilis PS216 ctg71800000...     100  2e-19
ref|NZ_AQGR01000028.1| Bacillus subtilis PS216 ctg71800000...      78  1e-12

 Bacillus subtilis Hal1 [firmicutes] taxid 1201039
ref|NZ_AMCA01000128.1| Bacillus subtilis Hal1 Contig130, w...     127  2e-28
ref|NZ_AMCA01000075.1| Bacillus subtilis Hal1 Contig77, wh...     122  6e-27
ref|NZ_AMCA01000033.1| Bacillus subtilis Hal1 Contig35, wh...     103  2e-20

 Staphylococcus epidermidis NIHLM040 [firmicutes] taxid 979208
ref|NZ_AKGR01000023.1| Staphylococcus epidermidis NIHLM040...     127  3e-28
ref|NZ_AKGR01000007.1| Staphylococcus epidermidis NIHLM040...     118  2e-25
ref|NZ_AKGR01000037.1| Staphylococcus epidermidis NIHLM040...     112  2e-23
ref|NZ_AKGR01000026.1| Staphylococcus epidermidis NIHLM040...     100  3e-19

 Bacillus subtilis subsp. spizizenii DV1-B-1 [firmicutes] taxid 1051503
ref|NZ_AFSG01000015.1| Bacillus subtilis subsp. spizizenii...     126  3e-28

 Prauserella sp. Am3 [high GC Gram+] taxid 1515610
ref|NZ_JTJI01000012.1| Prauserella sp. Am3 HQ32_scaffold_1...     126  3e-28

 Bacillus amyloliquefaciens TA208 [firmicutes] taxid 999891
ref|NC_017188.1| Bacillus amyloliquefaciens TA208 chromoso...     126  3e-28

 Bacillus amyloliquefaciens XH7 [firmicutes] taxid 1034836
ref|NC_017191.1| Bacillus amyloliquefaciens XH7 chromosome...     126  3e-28

 Bacillus amyloliquefaciens DSM 7 [firmicutes] taxid 692420
ref|NC_014551.1| Bacillus amyloliquefaciens DSM 7, complet...     126  3e-28

 Bacillus amyloliquefaciens LL3 [firmicutes] taxid 1001582
ref|NC_017190.1| Bacillus amyloliquefaciens LL3 chromosome...     126  3e-28

 Prauserella rugosa [high GC Gram+] taxid 43354
ref|NZ_JOIJ01000001.1| Prauserella rugosa strain NRRL B-22...     126  3e-28

 Bacillus amyloliquefaciens CC178 [firmicutes] taxid 1412898
ref|NC_022653.1| Bacillus amyloliquefaciens CC178, complet...     126  5e-28

 Bacillus amyloliquefaciens subsp. plantarum str. FZB42 [firmicutes] taxid
326423
ref|NC_009725.1| Bacillus amyloliquefaciens FZB42, complet...     126  5e-28

 Bacillus amyloliquefaciens [firmicutes] taxid 1390
ref|NZ_JXAT01000008.1| Bacillus amyloliquefaciens strain L...     125  7e-28
ref|NZ_JMEG01000044.1| Bacillus amyloliquefaciens strain B...     124  3e-27
ref|NZ_JQNZ01000020.1| Bacillus amyloliquefaciens strain X...     124  3e-27
ref|NZ_JXAT01000009.1| Bacillus amyloliquefaciens strain L...     122  1e-26
ref|NZ_JMEG01000008.1| Bacillus amyloliquefaciens strain B...     121  2e-26
ref|NZ_JMEG01000043.1| Bacillus amyloliquefaciens strain B...     115  1e-24

 Bacillus sp. MSP13 [firmicutes] taxid 1071061
ref|NZ_JXAQ01000003.1| Bacillus sp. MSP13 contig00006, who...     125  7e-28
ref|NZ_JXAQ01000004.1| Bacillus sp. MSP13 contig00007, who...     120  5e-26

 Bacillus vallismortis DV1-F-3 [firmicutes] taxid 1051502
ref|NZ_JH600259.1| Bacillus vallismortis DV1-F-3 scf718000...     125  8e-28
ref|NZ_JH600223.1| Bacillus vallismortis DV1-F-3 scf718000...     122  6e-27
ref|NZ_JH600262.1| Bacillus vallismortis DV1-F-3 scf718000...     103  1e-20

 Staphylococcus epidermidis AP027 [firmicutes] taxid 1345000
ref|NZ_ATCU02000103.1| Staphylococcus epidermidis AP027 CO...     124  8e-28
ref|NZ_ATCU02000225.1| Staphylococcus epidermidis AP027 CO...     113  6e-24
ref|NZ_ATCU02000185.1| Staphylococcus epidermidis AP027 CO...     101  1e-19

 Staphylococcus epidermidis VCU014 [firmicutes] taxid 904316
ref|NZ_JHQB01000008.1| Staphylococcus epidermidis VCU014 c...     125  1e-27
ref|NZ_JHQB01000004.1| Staphylococcus epidermidis VCU014 c...     114  4e-24
ref|NZ_JHQB01000007.1| Staphylococcus epidermidis VCU014 c...     109  2e-22
```

ref|NZ_JHQB01000032.1| Staphylococcus epidermidis VCU014 c... 100 2e-19

Staphylococcus epidermidis VCU013 [firmicutes] taxid 904315
ref|NZ_JHTZ01000095.1| Staphylococcus epidermidis VCU013 c... 123 1e-27
ref|NZ_JHTZ01000142.1| Staphylococcus epidermidis VCU013 c... 114 4e-24
ref|NZ_JHTZ01000007.1| Staphylococcus epidermidis VCU013 c... 112 2e-23
ref|NZ_JHTZ01000107.1| Staphylococcus epidermidis VCU013 c... 100 2e-19

Bacillus subtilis GB03 [firmicutes] taxid 1423687
ref|NZ_AYTJ01000008.1| Bacillus subtilis GB03 contig9, who... 125 1e-27

Bacillus sp. 5B6 [firmicutes] taxid 1127743
ref|NZ_AJST01000001.1| Bacillus sp. 5B6 5B6, whole genome ... 125 1e-27

Bacillus subtilis gtP20b [firmicutes] taxid 903507
ref|NZ_AEHM01000034.1| Bacillus subtilis gtP20b, whole gen... 124 1e-27
ref|NZ_AEHM01000035.1| Bacillus subtilis gtP20b, whole gen... 122 1e-26
ref|NZ_AEHM01000036.1| Bacillus subtilis gtP20b, whole gen... 106 2e-21

Bacillus amyloliquefaciens HB-26 [firmicutes] taxid 1366051
ref|NZ_AUWK01000006.1| Bacillus amyloliquefaciens HB-26 co... 125 1e-27
ref|NZ_AUWK01000003.1| Bacillus amyloliquefaciens HB-26 co... 122 8e-27

Bacillus amyloliquefaciens subsp. plantarum [firmicutes] taxid 1054400
ref|NZ_JOKF01000009.1| Bacillus amyloliquefaciens subsp. p... 125 1e-27
ref|NZ_JFBZ01000032.1| Bacillus amyloliquefaciens subsp. p... 123 3e-27
ref|NZ_JOKF01000005.1| Bacillus amyloliquefaciens subsp. p... 122 8e-27
ref|NZ_JFBZ01000008.1| Bacillus amyloliquefaciens subsp. p... 121 2e-26
ref|NZ_JFBZ01000006.1| Bacillus amyloliquefaciens subsp. p... 117 3e-25

Bacillus amyloliquefaciens subsp. plantarum UCMB5036 [firmicutes] taxid 1150475
ref|NC_020410.1| Bacillus amyloliquefaciens subsp. plantar... 125 1e-27

Bacillus subtilis subsp. inaquosorum KCTC 13429 [firmicutes] taxid 1236548
ref|NZ_AMXN01000004.1| Bacillus subtilis subsp. inaquosoru... 124 1e-27
ref|NZ_AMXN01000003.1| Bacillus subtilis subsp. inaquosoru... 123 5e-27

Laceyella sacchari 1-1 [firmicutes] taxid 1341151
ref|NZ_ASZU01000019.1| Laceyella sacchari 1-1 GS_11_TGACCA... 124 1e-27

Staphylococcus haemolyticus R1P1 [firmicutes] taxid 1134914
ref|NZ_AJVA01000007.1| Staphylococcus haemolyticus R1P1 co... 124 1e-27
ref|NZ_AJVA01000002.1| Staphylococcus haemolyticus R1P1 co... 95 2e-17

Staphylococcus haemolyticus DNF00585 [firmicutes] taxid 1401071
ref|NZ_JRNK01000002.1| Staphylococcus haemolyticus DNF0058... 124 1e-27
ref|NZ_JRNK01000023.1| Staphylococcus haemolyticus DNF0058... 94 3e-17

Bacillus subtilis subsp. subtilis str. AUSI98 [firmicutes] taxid 565143
ref|NZ_JH600128.1| Bacillus subtilis subsp. subtilis str. ... 124 2e-27
ref|NZ_JH600148.1| Bacillus subtilis subsp. subtilis str. ... 124 3e-27
ref|NZ_JH600102.1| Bacillus subtilis subsp. subtilis str. ... 122 6e-27
ref|NZ_JH600161.1| Bacillus subtilis subsp. subtilis str. ... 100 2e-19

Staphylococcus capitis C87 [firmicutes] taxid 435838
ref|NZ_GL545272.1| Staphylococcus capitis C87 supercont1.6... 124 2e-27
ref|NZ_GL545278.1| Staphylococcus capitis C87 supercont1.1... 116 8e-25

Staphylococcus haemolyticus [firmicutes] taxid 1283
ref|NZ_JQHB01000009.1| Staphylococcus haemolyticus strain ... 124 2e-27
ref|NZ_JQHA01000016.1| Staphylococcus haemolyticus strain ... 124 2e-27
ref|NZ_JFOJ01000010.1| Staphylococcus haemolyticus strain ... 123 5e-27
ref|NZ_JPRW01000013.1| Staphylococcus haemolyticus strain ... 122 9e-27
ref|NZ_JQHB01000038.1| Staphylococcus haemolyticus strain ... 105 3e-22
ref|NZ_JQHA01000032.1| Staphylococcus haemolyticus strain ... 105 1e-21
ref|NZ_JPRW01000064.1| Staphylococcus haemolyticus strain ... 95 2e-17
ref|NZ_JQHB01000010.1| Staphylococcus haemolyticus strain ... 92 2e-16
ref|NZ_JQHA01000029.1| Staphylococcus haemolyticus strain ... 92 2e-16
ref|NZ_JFOJ01000002.1| Staphylococcus haemolyticus strain ... 90 7e-16

Bacillus subtilis subsp. subtilis str. MP9 [firmicutes] taxid 1310157
ref|NZ_APMW01000149.1| Bacillus subtilis subsp. subtilis s... 124 2e-27
ref|NZ_APMW01000171.1| Bacillus subtilis subsp. subtilis s... 122 6e-27
ref|NZ_APMW01000130.1| Bacillus subtilis subsp. subtilis s... 102 6e-20

Bacillus amyloliquefaciens Y2 [firmicutes] taxid 1126211
ref|NC_017912.1| Bacillus amyloliquefaciens Y2 chromosome,... 124 2e-27

Bacillus amyloliquefaciens subsp. plantarum YAU B9601-Y2 [firmicutes] taxid 1155777
ref|NC_017061.1| Bacillus amyloliquefaciens subsp. plantar... 124 2e-27

Bacillus amyloliquefaciens subsp. plantarum NAU-B3 [firmicutes] taxid 1385727
ref|NC_022530.1| Bacillus amyloliquefaciens subsp. plantar... 124 2e-27

Bacillus subtilis SPZ1 [firmicutes] taxid 1316929
ref|NZ_AQGM01000011.1| Bacillus subtilis SPZ1 contig11, wh... 124 2e-27
ref|NZ_AQGM01000002.1| Bacillus subtilis SPZ1 contig2, who... 122 1e-26
ref|NZ_AQGM01000004.1| Bacillus subtilis SPZ1 contig4, who... 122 1e-26
ref|NZ_AQGM01000023.1| Bacillus subtilis SPZ1 contig24, wh... 112 2e-23

Paenibacillus polymyxa ATCC 12321 [firmicutes] taxid 1206104
ref|NZ_ARYD01000011.1| Paenibacillus polymyxa ATCC 12321 c... 124 2e-27
ref|NZ_ARYD01000003.1| Paenibacillus polymyxa ATCC 12321 c... 122 1e-26
ref|NZ_ARYD01000005.1| Paenibacillus polymyxa ATCC 12321 c... 122 1e-26
ref|NZ_ARYD01000023.1| Paenibacillus polymyxa ATCC 12321 c... 112 2e-23

Bacillus amyloliquefaciens EGD-AQ14 [firmicutes] taxid 1390372
ref|NZ_AVQH01000001.1| Bacillus amyloliquefaciens EGD-AQ14... 124 2e-27
ref|NZ_AVQH01000005.1| Bacillus amyloliquefaciens EGD-AQ14... 122 9e-27

Bacillus subtilis subsp. subtilis str. MP11 [firmicutes] taxid 1310431
ref|NZ_APMX01000205.1| Bacillus subtilis subsp. subtilis s... 124 2e-27
ref|NZ_APMX01000173.1| Bacillus subtilis subsp. subtilis s... 122 6e-27
ref|NZ_APMX01000371.1| Bacillus subtilis subsp. subtilis s... 102 6e-20

```
 Bacillus subtilis subsp. subtilis str. BAB-1 [firmicutes] taxid 1302650
ref|NC_020832.1| Bacillus subtilis subsp. subtilis str. BA...     124  3e-27

 Bacillus subtilis XF-1 [firmicutes] taxid 1233100
ref|NC_020244.1| Bacillus subtilis XF-1, complete genome          124  3e-27

 Bacillus subtilis S1-4 [firmicutes] taxid 1230477
ref|NZ_ANIP01000004.1| Bacillus subtilis S1-4 S1-4_3, whol...     124  3e-27
ref|NZ_ANIP01000002.1| Bacillus subtilis S1-4 S1-4_1, whol...     122  6e-27
ref|NZ_ANIP01000050.1| Bacillus subtilis S1-4 S1-4_50, who...     102  4e-20

 Sporosarcina pasteurii NCIM 2477 [firmicutes] taxid 1415631
ref|NZ_AYOX01000006.1| Sporosarcina pasteurii NCIM 2477 co...     124  3e-27
ref|NZ_AYOX01000011.1| Sporosarcina pasteurii NCIM 2477 co...     122  6e-27
ref|NZ_AYOX01000016.1| Sporosarcina pasteurii NCIM 2477 co...     100  3e-19

 Bacillus subtilis subsp. subtilis [firmicutes] taxid 135461
ref|NZ_JPVW01000002.1| Bacillus subtilis subsp. subtilis s...     124  3e-27
ref|NZ_JPVX01000003.1| Bacillus subtilis subsp. subtilis s...     124  3e-27
ref|NZ_JPVX01000007.1| Bacillus subtilis subsp. subtilis s...     100  2e-19
ref|NZ_JPVW01000001.1| Bacillus subtilis subsp. subtilis s...     100  2e-19

 Bacillus subtilis PTS-394 [firmicutes] taxid 1398775
ref|NZ_AWXG01000021.1| Bacillus subtilis PTS-394 contig26,...     124  3e-27

 Bacillus subtilis QH-1 [firmicutes] taxid 1437006
ref|NZ_AZQS01000003.1| Bacillus subtilis QH-1 contig3, who...     124  3e-27

 Bacillus subtilis PY79 [firmicutes] taxid 1415167
ref|NC_022898.1| Bacillus subtilis PY79, complete genome          124  3e-27

 Bacillus subtilis subsp. subtilis str. BSP1 [firmicutes] taxid 1192196
ref|NC_019896.1| Bacillus subtilis subsp. subtilis str. BS...     124  3e-27

 Bacillus subtilis BSn5 [firmicutes] taxid 936156
ref|NC_014976.1| Bacillus subtilis BSn5 chromosome, comple...     124  3e-27

 Staphylococcus epidermidis APO35 [firmicutes] taxid 1344999
ref|NZ_KE365706.1| Staphylococcus epidermidis APO35 genomi...     123  3e-27
ref|NZ_ATCV02000094.1| Staphylococcus epidermidis APO35 CO...     122  1e-26
ref|NZ_KE365723.1| Staphylococcus epidermidis APO35 genomi...     113  5e-24
ref|NZ_ATCV02000003.1| Staphylococcus epidermidis APO35 CO...     113  8e-24
ref|NZ_KE365923.1| Staphylococcus epidermidis APO35 genomi...     101  1e-19
ref|NZ_ATCV02000198.1| Staphylococcus epidermidis APO35 CO...     101  1e-19

 Staphylococcus phage StB20 [viruses] taxid 1147043
ref|NC_019915.1| Staphylococcus phage StB20, complete genome      123  4e-27

 Staphylococcus epidermidis Scl22 [firmicutes] taxid 1344993
ref|NZ_KE366975.1| Staphylococcus epidermidis Scl22 Scl22_...     123  4e-27
ref|NZ_KE367242.1| Staphylococcus epidermidis Scl22 Scl22_...     113  6e-24
ref|NZ_KE367222.1| Staphylococcus epidermidis Scl22 Scl22_...     101  1e-19

 Staphylococcus epidermidis MC28 [firmicutes] taxid 1344995
ref|NZ_ATCZ02000008.1| Staphylococcus epidermidis MC28 CON...     123  4e-27
ref|NZ_ATCZ02000033.1| Staphylococcus epidermidis MC28 CON...     113  3e-24
ref|NZ_ATCZ02000360.1| Staphylococcus epidermidis MC28 CON...     101  1e-19

 Staphylococcus epidermidis MC16 [firmicutes] taxid 1344997
ref|NZ_ATCX02000236.1| Staphylococcus epidermidis MC16 CON...     123  4e-27
ref|NZ_ATCX02000231.1| Staphylococcus epidermidis MC16 CON...     113  8e-24
ref|NZ_ATCX02000061.1| Staphylococcus epidermidis MC16 CON...     101  1e-19

 Staphylococcus epidermidis CIM37 [firmicutes] taxid 1344990
ref|NZ_ATDE02000267.1| Staphylococcus epidermidis CIM37 CO...     123  4e-27
ref|NZ_ATDE02000255.1| Staphylococcus epidermidis CIM37 CO...     113  8e-24
ref|NZ_ATDE02000033.1| Staphylococcus epidermidis CIM37 CO...     101  1e-19

 Staphylococcus epidermidis MC19 [firmicutes] taxid 1344996
ref|NZ_ATCY02000137.1| Staphylococcus epidermidis MC19 CON...     123  4e-27
ref|NZ_ATCY02000213.1| Staphylococcus epidermidis MC19 CON...     113  7e-24
ref|NZ_ATCY02000104.1| Staphylococcus epidermidis MC19 CON...     101  1e-19

 Staphylococcus epidermidis CIM40 [firmicutes] taxid 1344998
ref|NZ_ATCW02000040.1| Staphylococcus epidermidis CIM40 CO...     123  4e-27
ref|NZ_ATCW02000079.1| Staphylococcus epidermidis CIM40 CO...     113  8e-24
ref|NZ_ATCW02000090.1| Staphylococcus epidermidis CIM40 CO...     101  1e-19

 Staphylococcus epidermidis Scl19 [firmicutes] taxid 1344994
ref|NZ_ATDA02000047.1| Staphylococcus epidermidis Scl19 CO...     123  4e-27
ref|NZ_ATDA02000118.1| Staphylococcus epidermidis Scl19 CO...     113  8e-24

 Staphylococcus epidermidis FRI909 [firmicutes] taxid 764544
ref|NZ_AENR01000028.1| Staphylococcus epidermidis FRI909 c...     123  4e-27
ref|NZ_AENR01000001.1| Staphylococcus epidermidis FRI909 c...     113  8e-24
ref|NZ_AENR01000003.1| Staphylococcus epidermidis FRI909 c...     101  1e-19

 Staphylococcus epidermidis WI05 [firmicutes] taxid 1344988
ref|NZ_ATDG02000039.1| Staphylococcus epidermidis WI05 CON...     123  4e-27
ref|NZ_ATDG02000024.1| Staphylococcus epidermidis WI05 CON...     113  8e-24
ref|NZ_ATDG02000032.1| Staphylococcus epidermidis WI05 CON...     101  2e-19

 Staphylococcus epidermidis WI09 [firmicutes] taxid 1344987
ref|NZ_ATDH02000068.1| Staphylococcus epidermidis WI09 CON...     123  4e-27

 Staphylococcus epidermidis CIM28 [firmicutes] taxid 1344989
ref|NZ_ATDF02000020.1| Staphylococcus epidermidis CIM28 CO...     123  4e-27

 Staphylococcus epidermidis VCU144 [firmicutes] taxid 904347
ref|NZ_AFED01000050.1| Staphylococcus epidermidis VCU144 c...     123  5e-27
ref|NZ_AFED01000013.1| Staphylococcus epidermidis VCU144 c...     114  4e-24
ref|NZ_AFED01000071.1| Staphylococcus epidermidis VCU144 c...     112  2e-23
ref|NZ_AFED01000044.1| Staphylococcus epidermidis VCU144 c...     100  2e-19

 Staphylococcus haemolyticus JCSC1435 [firmicutes] taxid 279808
ref|NC_007168.1| Staphylococcus haemolyticus JCSC1435 chro...     123  5e-27

 Staphylococcus epidermidis 12142587 [firmicutes] taxid 1235440
```

```
ref|NZ_AMSJ01000004.1| Staphylococcus epidermidis 12142587...     123  5e-27
ref|NZ_AMSJ01000013.1| Staphylococcus epidermidis 12142587...     119  9e-26
ref|NZ_AMSJ01000016.1| Staphylococcus epidermidis 12142587...     116  1e-24
ref|NZ_AMSJ01000008.1| Staphylococcus epidermidis 12142587...     112  2e-23
ref|NZ_AMSJ01000001.1| Staphylococcus epidermidis 12142587...     100  2e-19

 Bacillus subtilis subsp. spizizenii ATCC 6633 [firmicutes] taxid 703612
ref|NZ_ADGS01000011.1| Bacillus subtilis subsp. spizizenii...     123  5e-27
ref|NZ_ADGS01000013.1| Bacillus subtilis subsp. spizizenii...     120  5e-26

 Staphylococcus epidermidis VCU065 [firmicutes] taxid 904324
ref|NZ_AHKZ01000069.1| Staphylococcus epidermidis VCU065 c...     123  5e-27
ref|NZ_AHKZ01000078.1| Staphylococcus epidermidis VCU065 c...     119  1e-25
ref|NZ_AHKZ01000006.1| Staphylococcus epidermidis VCU065 c...     109  2e-22
ref|NZ_AHKZ01000054.1| Staphylococcus epidermidis VCU065 c...     100  2e-19

 Staphylococcus epidermidis VCU123 [firmicutes] taxid 904340
ref|NZ_AHLE01000021.1| Staphylococcus epidermidis VCU123 c...     123  5e-27
ref|NZ_AHLE01000028.1| Staphylococcus epidermidis VCU123 c...     114  4e-24
ref|NZ_AHLE01000015.1| Staphylococcus epidermidis VCU123 c...     112  2e-23
ref|NZ_AHLE01000042.1| Staphylococcus epidermidis VCU123 c...     101  1e-19

 Bacillus subtilis subsp. spizizenii str. W23 [firmicutes] taxid 655816
ref|NC_014479.1| Bacillus subtilis subsp. spizizenii str. ...     123  5e-27

 Staphylococcus epidermidis [firmicutes] taxid 1282
ref|NZ_JMID01000001.1| Staphylococcus epidermidis strain 1...     123  5e-27
ref|NZ_JMIF01000013.1| Staphylococcus epidermidis strain 8...     114  4e-24
ref|NZ_JMIE01000037.1| Staphylococcus epidermidis strain 1...     114  4e-24
ref|NZ_JMID01000026.1| Staphylococcus epidermidis strain 1...     114  4e-24
ref|NZ_JGVL01000063.1| Staphylococcus epidermidis strain E...     114  4e-24
ref|NZ_JGVL01000016.1| Staphylococcus epidermidis strain E...     112  2e-23
ref|NZ_JMIF01000020.1| Staphylococcus epidermidis strain 8...     112  2e-23
ref|NZ_JMIE01000045.1| Staphylococcus epidermidis strain 1...     109  2e-22
ref|NZ_JMID01000019.1| Staphylococcus epidermidis strain 1...     109  2e-22
ref|NZ_JGVL01000026.1| Staphylococcus epidermidis strain E...     100  2e-19
ref|NZ_JMID01000021.1| Staphylococcus epidermidis strain 1...     100  2e-19
ref|NZ_JMIF01000001.1| Staphylococcus epidermidis strain 8...     100  2e-19
ref|NZ_JMIE01000004.1| Staphylococcus epidermidis strain 1...     100  2e-19

 Staphylococcus epidermidis NIHLM015 [firmicutes] taxid 979216
ref|NZ_AKGZ01000003.1| Staphylococcus epidermidis NIHLM015...     123  5e-27
ref|NZ_AKGZ01000032.1| Staphylococcus epidermidis NIHLM015...     121  2e-26
ref|NZ_AKGZ01000002.1| Staphylococcus epidermidis NIHLM015...     113  8e-24

 Staphylococcus epidermidis M23864:W2(grey) [firmicutes] taxid 525375
ref|NZ_GG749255.1| Staphylococcus epidermidis M23864:W2(gr...     123  5e-27
ref|NZ_GG749256.1| Staphylococcus epidermidis M23864:W2(gr...     112  2e-23

 Bacillus sp. JS [firmicutes] taxid 1127744
ref|NC_017743.1| Bacillus sp. JS chromosome, complete genome      123  6e-27

 Bacillus methylotrophicus SK19.001 [firmicutes] taxid 1281778
ref|NZ_AOFO01000017.1| Bacillus methylotrophicus SK19.001 ...     122  6e-27
ref|NZ_AOFO01000003.1| Bacillus methylotrophicus SK19.001 ...     122  9e-27
ref|NZ_AOFO01000002.1| Bacillus methylotrophicus SK19.001 ...     122  1e-26

 Bacillus amyloliquefaciens subsp. amyloliquefaciens DC-12 [firmicutes] taxid
1198291
ref|NZ_KB206086.1| Bacillus amyloliquefaciens subsp. amylo...     122  6e-27

 Staphylococcus epidermidis NIHLM037 [firmicutes] taxid 979210
ref|NZ_AKGT01000004.1| Staphylococcus epidermidis NIHLM037...     122  6e-27
ref|NZ_AKGT01000046.1| Staphylococcus epidermidis NIHLM037...     121  2e-26
ref|NZ_AKGT01000040.1| Staphylococcus epidermidis NIHLM037...     113  8e-24
ref|NZ_AKGT01000014.1| Staphylococcus epidermidis NIHLM037...     101  1e-19

 Bacillus subtilis Miyagi-4 [firmicutes] taxid 1227747
ref|NZ_BALZ01000026.1| Bacillus subtilis Miyagi-4, whole g...     122  6e-27
ref|NZ_BALZ01000210.1| Bacillus subtilis Miyagi-4, whole g...     120  1e-26
ref|NZ_BALZ01000287.1| Bacillus subtilis Miyagi-4, whole g...     102  8e-20

 Halobacillus sp. BBL2006 [firmicutes] taxid 1543706
ref|NZ_JRNX01000495.1| Halobacillus sp. BBL2006 cont499, w...     122  6e-27

 Staphylococcus epidermidis VCU071 [firmicutes] taxid 904325
ref|NZ_AGUB01000030.1| Staphylococcus epidermidis VCU071 c...     122  6e-27
ref|NZ_AGUB01000020.1| Staphylococcus epidermidis VCU071 c...     119  9e-26
ref|NZ_AGUB01000008.1| Staphylococcus epidermidis VCU071 c...     100  3e-19

 Bacillus subtilis subsp. subtilis str. RO-NN-1 [firmicutes] taxid 1052588
ref|NC_017195.1| Bacillus subtilis subsp. subtilis str. RO...     122  6e-27

 Bacillus subtilis subsp. natto BEST195 [firmicutes] taxid 645657
ref|NC_017196.2| Bacillus subtilis subsp. natto BEST195 DN...     122  6e-27

 Staphylococcus epidermidis NIHLM031 [firmicutes] taxid 979214
ref|NZ_AKGX01000043.1| Staphylococcus epidermidis NIHLM031...     122  7e-27
ref|NZ_AKGX01000022.1| Staphylococcus epidermidis NIHLM031...     113  8e-24
ref|NZ_AKGX01000009.1| Staphylococcus epidermidis NIHLM031...     101  1e-19

 Staphylococcus sp. EGD-HP3 [firmicutes] taxid 1357269
ref|NZ_AVOQ01000023.1| Staphylococcus sp. EGD-HP3 contig3,...     122  7e-27
ref|NZ_AVOQ01000001.1| Staphylococcus sp. EGD-HP3 contig1,...     102  5e-20

 Bacillus tequilensis KCTC 13622 [firmicutes] taxid 1423686
ref|NZ_AYTO01000041.1| Bacillus tequilensis KCTC 13622 con...     122  8e-27

 Staphylococcus warneri Lyso 1 2011 [firmicutes] taxid 1448849
ref|NZ_JOPU01000020.1| Staphylococcus warneri Lyso 1 2011 ...     122  8e-27
ref|NZ_JOPU01000043.1| Staphylococcus warneri Lyso 1 2011 ...     104  9e-21
ref|NZ_JOPU01000040.1| Staphylococcus warneri Lyso 1 2011 ...      87  5e-15

 Staphylococcus warneri Lyso 2 2011 [firmicutes] taxid 1448850
ref|NZ_JOPV01000018.1| Staphylococcus warneri Lyso 2 2011 ...     122  8e-27
ref|NZ_JOPV01000037.1| Staphylococcus warneri Lyso 2 2011 ...     104  9e-21
ref|NZ_JOPV01000035.1| Staphylococcus warneri Lyso 2 2011 ...      87  5e-15
```

Staphylococcus warneri [firmicutes] taxid 1292
ref|NZ_JPOW01000002.1| Staphylococcus warneri strain NGS-E... 122 8e-27
ref|NZ_JPOW01000001.1| Staphylococcus warneri strain NGS-E... 104 1e-20

Staphylococcus warneri SG1 [firmicutes] taxid 1194526
ref|NC_020164.1| Staphylococcus warneri SG1, complete genome 122 8e-27

Streptomyces aureocirculatus [high GC Gram+] taxid 67275
ref|NZ_JOAP01000023.1| Streptomyces aureocirculatus strain... 122 9e-27
ref|NZ_JOAR01000021.1| Streptomyces aureocirculatus strain... 122 9e-27
ref|NZ_JOAP01000001.1| Streptomyces aureocirculatus strain... 92 2e-16
ref|NZ_JOAR01000001.1| Streptomyces aureocirculatus strain... 92 2e-16

Staphylococcus epidermidis UC7032 [firmicutes] taxid 1327991
ref|NZ_ARWU01000033.1| Staphylococcus epidermidis UC7032 N... 122 9e-27
ref|NZ_ARWU01000110.1| Staphylococcus epidermidis UC7032 N... 113 9e-24
ref|NZ_ARWU01000087.1| Staphylococcus epidermidis UC7032 N... 101 1e-19

Staphylococcus warneri A487 [firmicutes] taxid 904143
ref|NZ_CANQ01000033.1| Staphylococcus warneri A487, whole ... 122 9e-27
ref|NZ_CANQ01000011.1| Staphylococcus warneri A487, whole ... 104 9e-21
ref|NZ_CANQ01000019.1| Staphylococcus warneri A487, whole ... 87 5e-15

Bacillus sp. UNC69MF [firmicutes] taxid 1449047
ref|NZ_KN050798.1| Bacillus sp. UNC69MF BR62DRAFT_scaffold... 122 9e-27

Bacillus amyloliquefaciens subsp. plantarum AS43.3 [firmicutes] taxid 1225788
ref|NC_019842.1| Bacillus amyloliquefaciens subsp. plantar... 122 9e-27

Staphylococcus pasteuri SP1 [firmicutes] taxid 1276282
ref|NC_022737.1| Staphylococcus pasteuri SP1, complete genome 122 1e-26

Bacillus mojavensis RO-H-1 = KCTC 3706 [firmicutes] taxid 1051501
ref|NZ_JH600294.1| Bacillus mojavensis RO-H-1 = KCTC 3706 ... 122 1e-26
ref|NZ_AYTL01000027.1| Bacillus mojavensis RO-H-1 = KCTC 3... 122 1e-26
ref|NZ_JH600286.1| Bacillus mojavensis RO-H-1 = KCTC 3706 ... 114 7e-24
ref|NZ_AYTL01000033.1| Bacillus mojavensis RO-H-1 = KCTC 3... 109 2e-22

Bacillus amyloliquefaciens UASWS BA1 [firmicutes] taxid 1384051
ref|NZ_AWQY01000002.1| Bacillus amyloliquefaciens UASWS BA... 122 1e-26

Bacillus amyloliquefaciens subsp. plantarum M27 [firmicutes] taxid 1177186
ref|NZ_AMPK01000010.1| Bacillus amyloliquefaciens subsp. p... 122 1e-26

Staphylococcus epidermidis VCU118 [firmicutes] taxid 904336
ref|NZ_AHLB01000044.1| Staphylococcus epidermidis VCU118 c... 122 1e-26
ref|NZ_AHLB01000002.1| Staphylococcus epidermidis VCU118 c... 113 8e-24
ref|NZ_AHLB01000066.1| Staphylococcus epidermidis VCU118 c... 101 1e-19

Bacillus amyloliquefaciens IT-45 [firmicutes] taxid 1091041
ref|NC_020272.1| Bacillus amyloliquefaciens IT-45, complet... 122 1e-26

Bacillus amyloliquefaciens LFB112 [firmicutes] taxid 1415165
ref|NC_023073.1| Bacillus amyloliquefaciens LFB112, comple... 122 1e-26

Bacillus sp. 916 [firmicutes] taxid 1007654
ref|NZ_AFSU01000107.1| Bacillus sp. 916 Contig349, whole g... 122 1e-26
ref|NZ_AFSU01000002.1| Bacillus sp. 916 Contig2, whole gen... 121 1e-26

Bacillus amyloliquefaciens EBL11 [firmicutes] taxid 1457158
ref|NZ_JCOC01000001.1| Bacillus amyloliquefaciens EBL11 NO... 122 1e-26
ref|NZ_JCOC01000002.1| Bacillus amyloliquefaciens EBL11 NO... 120 4e-26

Bacillus amyloliquefaciens subsp. plantarum UCMB5113 [firmicutes] taxid 1150476
ref|NC_022081.1| Bacillus amyloliquefaciens subsp. plantar... 122 1e-26

Bacillus amyloliquefaciens subsp. plantarum UCMB5033 [firmicutes] taxid 1338518
ref|NC_022075.1| Bacillus amyloliquefaciens subsp. plantar... 122 1e-26

Staphylococcus arlettae CVD059 [firmicutes] taxid 1212545
ref|NZ_ALWK01000016.1| Staphylococcus arlettae CVD059 SARL... 122 1e-26
ref|NZ_ALWK01000026.1| Staphylococcus arlettae CVD059 SARL... 102 4e-20
ref|NZ_ALWK01000057.1| Staphylococcus arlettae CVD059 SARL... 102 5e-20

Staphylococcus epidermidis VCU129 [firmicutes] taxid 904345
ref|NZ_AHLJ01000004.1| Staphylococcus epidermidis VCU129 c... 121 2e-26
ref|NZ_AHLJ01000018.1| Staphylococcus epidermidis VCU129 c... 113 8e-24
ref|NZ_AHLJ01000026.1| Staphylococcus epidermidis VCU129 c... 101 1e-19

Bacillus subtilis subsp. spizizenii TU-B-10 [firmicutes] taxid 1052585
ref|NC_016047.1| Bacillus subtilis subsp. spizizenii TU-B-... 121 2e-26

Bacillus amyloliquefaciens subsp. plantarum CAU B946 [firmicutes] taxid 1114958
ref|NC_016784.1| Bacillus amyloliquefaciens subsp. plantar... 121 2e-26

Staphylococcus epidermidis VCU128 [firmicutes] taxid 904344
ref|NZ_AHLI01000038.1| Staphylococcus epidermidis VCU128 c... 121 2e-26
ref|NZ_AHLI01000053.1| Staphylococcus epidermidis VCU128 c... 112 3e-23
ref|NZ_AHLI01000001.1| Staphylococcus epidermidis VCU128 c... 100 3e-19

Staphylococcus epidermidis NIHLM049 [firmicutes] taxid 979207
ref|NZ_AKGQ01000040.1| Staphylococcus epidermidis NIHLM049... 121 2e-26
ref|NZ_AKGQ01000050.1| Staphylococcus epidermidis NIHLM049... 114 4e-24
ref|NZ_AKGQ01000039.1| Staphylococcus epidermidis NIHLM049... 112 2e-23
ref|NZ_AKGQ01000042.1| Staphylococcus epidermidis NIHLM049... 100 2e-19

Staphylococcus epidermidis VCU041 [firmicutes] taxid 904320
ref|NZ_AHKX01000053.1| Staphylococcus epidermidis VCU041 c... 121 2e-26
ref|NZ_AHKX01000040.1| Staphylococcus epidermidis VCU041 c... 114 4e-24
ref|NZ_AHKX01000051.1| Staphylococcus epidermidis VCU041 c... 112 2e-23
ref|NZ_AHKX01000061.1| Staphylococcus epidermidis VCU041 c... 100 2e-19

Staphylococcus epidermidis NIH05001 [firmicutes] taxid 979221
ref|NZ_AKHE01000027.1| Staphylococcus epidermidis NIH05001... 121 2e-26

```
ref|NZ_AKHE01000086.1| Staphylococcus epidermidis NIH05001...     114  4e-24
ref|NZ_AKHE01000016.1| Staphylococcus epidermidis NIH05001...     100  2e-19

 Staphylococcus warneri L37603 [firmicutes] taxid 596319
ref|NZ_ACPZ01000067.1| Staphylococcus warneri L37603 conti...     120  4e-26
ref|NZ_ACPZ01000027.1| Staphylococcus warneri L37603 conti...     104  8e-21
ref|NZ_ACPZ01000035.1| Staphylococcus warneri L37603 conti...      88  1e-15

 Bacillus mojavensis RRC 101 [firmicutes] taxid 1329377
ref|NZ_ASJT01000048.1| Bacillus mojavensis RRC 101 contig_...     120  5e-26
ref|NZ_ASJT01000050.1| Bacillus mojavensis RRC 101 contig_...     117  6e-25
ref|NZ_ASJT01000031.1| Bacillus mojavensis RRC 101 contig_...     115  1e-24

 Staphylococcus epidermidis NIHLM061 [firmicutes] taxid 979204
ref|NZ_AKGN01000046.1| Staphylococcus epidermidis NIHLM061...     119  1e-25
ref|NZ_AKGN01000033.1| Staphylococcus epidermidis NIHLM061...     112  3e-23
ref|NZ_AKGN01000026.1| Staphylococcus epidermidis NIHLM061...     101  1e-19

 Staphylococcus epidermidis NIHLM023 [firmicutes] taxid 979211
ref|NZ_AKGU01000058.1| Staphylococcus epidermidis NIHLM023...     119  1e-25
ref|NZ_AKGU01000064.1| Staphylococcus epidermidis NIHLM023...     112  3e-23
ref|NZ_AKGU01000008.1| Staphylococcus epidermidis NIHLM023...     101  1e-19

 Staphylococcus epidermidis W23144 [firmicutes] taxid 525376
ref|NZ_GG696800.1| Staphylococcus epidermidis W23144 SCAFF...     119  1e-25
ref|NZ_GG696799.1| Staphylococcus epidermidis W23144 SCAFF...     112  3e-23

 Staphylococcus capitis VCU116 [firmicutes] taxid 904334
ref|NZ_AFTX01000006.1| Staphylococcus capitis VCU116 conti...     119  1e-25
ref|NZ_AFTX01000018.1| Staphylococcus capitis VCU116 conti...     114  4e-24
ref|NZ_AFTX01000038.1| Staphylococcus capitis VCU116 conti...     103  3e-20

 Staphylococcus epidermidis ATCC 12228 [firmicutes] taxid 176280
ref|NC_004461.1| Staphylococcus epidermidis ATCC 12228 chr...     119  1e-25

 Staphylococcus epidermidis NIH06004 [firmicutes] taxid 1155134
ref|NZ_AKHH01000010.1| Staphylococcus epidermidis NIH06004...     119  1e-25
ref|NZ_AKHH01000029.1| Staphylococcus epidermidis NIH06004...     112  2e-23
ref|NZ_AKHH01000025.1| Staphylococcus epidermidis NIH06004...     100  2e-19

 Staphylococcus epidermidis NIH08001 [firmicutes] taxid 1155135
ref|NZ_AKHG01000028.1| Staphylococcus epidermidis NIH08001...     119  1e-25
ref|NZ_AKHG01000011.1| Staphylococcus epidermidis NIH08001...     112  2e-23
ref|NZ_AKHG01000007.1| Staphylococcus epidermidis NIH08001...     100  2e-19

 Staphylococcus epidermidis M0026 [firmicutes] taxid 1388003
ref|NZ_KI999594.1| Staphylococcus epidermidis M0026 adAAZ-...     119  1e-25
ref|NZ_KI999659.1| Staphylococcus epidermidis M0026 adAAZ-...     112  2e-23
ref|NZ_KI999593.1| Staphylococcus epidermidis M0026 adAAZ-...     100  2e-19

 Staphylococcus epidermidis VCU045 [firmicutes] taxid 904321
ref|NZ_AFEI01000061.1| Staphylococcus epidermidis VCU045 c...     118  2e-25
ref|NZ_AFEI01000011.1| Staphylococcus epidermidis VCU045 c...     112  2e-23
ref|NZ_AFEI01000032.1| Staphylococcus epidermidis VCU045 c...     101  1e-19

 Staphylococcus epidermidis NIH04003 [firmicutes] taxid 1155132
ref|NZ_AKHJ01000028.1| Staphylococcus epidermidis NIH04003...     118  2e-25
ref|NZ_AKHJ01000008.1| Staphylococcus epidermidis NIH04003...     112  2e-23
ref|NZ_AKHJ01000013.1| Staphylococcus epidermidis NIH04003...     100  2e-19

 Staphylococcus epidermidis VCU037 [firmicutes] taxid 904319
ref|NZ_AFTY01000029.1| Staphylococcus epidermidis VCU037 c...     118  2e-25
ref|NZ_AFTY01000039.1| Staphylococcus epidermidis VCU037 c...     112  2e-23
ref|NZ_AFTY01000033.1| Staphylococcus epidermidis VCU037 c...     100  2e-19

 Bacillus flexus T6186-2 [firmicutes] taxid 1444310
ref|NZ_JANV01000167.1| Bacillus flexus T6186-2 contig_167,...     118  2e-25
ref|NZ_JANV01000124.1| Bacillus flexus T6186-2 contig_124,...      82  3e-13

 Staphylococcus epidermidis M0881 [firmicutes] taxid 1213731
ref|NZ_KB821726.1| Staphylococcus epidermidis M0881 acumm-...     118  2e-25
ref|NZ_KB821721.1| Staphylococcus epidermidis M0881 acumm-...     112  2e-23
ref|NZ_KB821722.1| Staphylococcus epidermidis M0881 acumm-...     100  2e-19

 Streptomyces sp. PRh5 [high GC Gram+] taxid 1158056
ref|NZ_JABQ01000003.1| Streptomyces sp. PRh5 contig003, wh...     118  2e-25

 Staphylococcus epidermidis NIHLM001 [firmicutes] taxid 979219
ref|NZ_AKHC01000040.1| Staphylococcus epidermidis NIHLM001...     118  2e-25
ref|NZ_AKHC01000049.1| Staphylococcus epidermidis NIHLM001...     111  4e-23
ref|NZ_AKHC01000045.1| Staphylococcus epidermidis NIHLM001...     110  1e-22
ref|NZ_AKHC01000052.1| Staphylococcus epidermidis NIHLM001...     100  2e-19

 Staphylococcus epidermidis RP62A [firmicutes] taxid 176279
ref|NC_002976.3| Staphylococcus epidermidis RP62A, complet...     118  2e-25

 Marinococcus halotolerans DSM 16375 [firmicutes] taxid 1122203
ref|NZ_ATVM01000015.1| Marinococcus halotolerans DSM 16375...     117  3e-25

 Staphylococcus sp. URHA0057 [firmicutes] taxid 1380374
ref|NZ_KK211097.1| Staphylococcus sp. URHA0057 N524DRAFT_s...     117  3e-25
ref|NZ_KK211096.1| Staphylococcus sp. URHA0057 N524DRAFT_s...      99  6e-19
ref|NZ_JHVS01000001.1| Staphylococcus sp. URHA0057 N524DRA...      89  1e-15

 Staphylococcus xylosus NJ [firmicutes] taxid 1262650
ref|NZ_ANMR01000019.1| Staphylococcus xylosus NJ contig19,...     117  3e-25
ref|NZ_ANMR01000043.1| Staphylococcus xylosus NJ contig43,...      96  6e-18

 Bacillus panaciterrae DSM 19096 [firmicutes] taxid 1121093
ref|NZ_AUMR01000003.1| Bacillus panaciterrae DSM 19096 H54...     117  4e-25

 Staphylococcus capitis [firmicutes] taxid 29388
ref|NZ_JGYJ01000079.1| Staphylococcus capitis strain LNZR-...     117  4e-25
ref|NZ_JGYJ01000014.1| Staphylococcus capitis strain LNZR-...     107  1e-21
ref|NZ_JGYJ01000072.1| Staphylococcus capitis strain LNZR-...     102  5e-20

 Staphylococcus capitis CR01 [firmicutes] taxid 1296619
ref|NZ_HG737333.1| Staphylococcus capitis CR01, whole geno...     117  4e-25
```

```
 Staphylococcus epidermidis NIHLM003 [firmicutes] taxid 979218
ref|NZ_AKHB01000022.1| Staphylococcus epidermidis NIHLM003...     117  4e-25
ref|NZ_AKHB01000030.1| Staphylococcus epidermidis NIHLM003...     112  3e-23
ref|NZ_AKHB01000036.1| Staphylococcus epidermidis NIHLM003...     100  2e-19

 Nocardiopsis xinjiangensis YIM 90004 [high GC Gram+] taxid 1246474
ref|NZ_ANBE01000006.1| Nocardiopsis xinjiangensis YIM 9000...     117  5e-25

 Staphylococcus epidermidis NIHLM053 [firmicutes] taxid 979206
ref|NZ_AKGP01000094.1| Staphylococcus epidermidis NIHLM053...     117  5e-25
ref|NZ_AKGP01000107.1| Staphylococcus epidermidis NIHLM053...     112  3e-23
ref|NZ_AKGP01000014.1| Staphylococcus epidermidis NIHLM053...      99  5e-19

 Staphylococcus epidermidis NIHLM057 [firmicutes] taxid 979205
ref|NZ_AKGO01000075.1| Staphylococcus epidermidis NIHLM057...     117  5e-25
ref|NZ_AKGO01000084.1| Staphylococcus epidermidis NIHLM057...     112  3e-23
ref|NZ_AKGO01000016.1| Staphylococcus epidermidis NIHLM057...      99  5e-19

 Staphylococcus sp. JGI 0001002-I23 [firmicutes] taxid 1157683
ref|NZ_AQWX01000161.1| Staphylococcus sp. JGI 0001002-I23 ...     117  5e-25
ref|NZ_AQWX01000183.1| Staphylococcus sp. JGI 0001002-I23 ...     112  2e-23
ref|NZ_AQWX01000172.1| Staphylococcus sp. JGI 0001002-I23 ...     103  2e-20

 Staphylococcus epidermidis NIHLM070 [firmicutes] taxid 979202
ref|NZ_AKGL01000008.1| Staphylococcus epidermidis NIHLM070...     117  6e-25
ref|NZ_AKGL01000047.1| Staphylococcus epidermidis NIHLM070...     112  3e-23
ref|NZ_AKGL01000013.1| Staphylococcus epidermidis NIHLM070...     100  2e-19

 Staphylococcus sp. MDS7B [firmicutes] taxid 1209359
ref|NZ_AOTH01000059.1| Staphylococcus sp. MDS7B Ion2_c59, ...     117  6e-25
ref|NZ_AOTH01000109.1| Staphylococcus sp. MDS7B Ion2_c109,...     112  2e-23
ref|NZ_AOTH01000002.1| Staphylococcus sp. MDS7B Ion2_c2, w...     109  2e-22
ref|NZ_AOTH01000129.1| Staphylococcus sp. MDS7B Ion2_c129,...     106  3e-21
ref|NZ_AOTH01000008.1| Staphylococcus sp. MDS7B Ion2_c8, w...     100  2e-19

 Staphylococcus epidermidis VCU125 [firmicutes] taxid 904341
ref|NZ_AHLF01000034.1| Staphylococcus epidermidis VCU125 c...     117  7e-25
ref|NZ_AHLF01000015.1| Staphylococcus epidermidis VCU125 c...     109  2e-22
ref|NZ_AHLF01000042.1| Staphylococcus epidermidis VCU125 c...     100  2e-19

 Staphylococcus epidermidis VCU127 [firmicutes] taxid 904343
ref|NZ_AHLH01000022.1| Staphylococcus epidermidis VCU127 c...     117  7e-25
ref|NZ_AHLH01000069.1| Staphylococcus epidermidis VCU127 c...     108  4e-22
ref|NZ_AHLH01000020.1| Staphylococcus epidermidis VCU127 c...     100  2e-19

 Staphylococcus epidermidis IS-250 [firmicutes] taxid 904789
ref|NZ_AJJR01000042.1| Staphylococcus epidermidis IS-250 c...     117  7e-25
ref|NZ_AJJR01000011.1| Staphylococcus epidermidis IS-250 c...     109  2e-22
ref|NZ_AJJR01000006.1| Staphylococcus epidermidis IS-250 c...     100  2e-19

 Staphylococcus epidermidis IS-K [firmicutes] taxid 904791
ref|NZ_AJJS01000034.1| Staphylococcus epidermidis IS-K con...     117  7e-25
ref|NZ_AJJS01000016.1| Staphylococcus epidermidis IS-K con...     109  2e-22
ref|NZ_AJJS01000014.1| Staphylococcus epidermidis IS-K con...     100  2e-19

 Staphylococcus epidermidis NIHLM008 [firmicutes] taxid 979217
ref|NZ_AKHA01000028.1| Staphylococcus epidermidis NIHLM008...     117  7e-25
ref|NZ_AKHA01000014.1| Staphylococcus epidermidis NIHLM008...     112  3e-23
ref|NZ_AKHA01000009.1| Staphylococcus epidermidis NIHLM008...     100  2e-19

 Staphylococcus epidermidis NIHLM088 [firmicutes] taxid 979200
ref|NZ_AKGJ01000032.1| Staphylococcus epidermidis NIHLM088...     117  7e-25
ref|NZ_AKGJ01000036.1| Staphylococcus epidermidis NIHLM088...     112  2e-23
ref|NZ_AKGJ01000014.1| Staphylococcus epidermidis NIHLM088...     100  2e-19

 Staphylococcus xylosus DMB3-Bh1 [firmicutes] taxid 1353979
ref|NZ_AURW01000006.1| Staphylococcus xylosus DMB3-Bh1 DMB...     117  7e-25
ref|NZ_AURW01000002.1| Staphylococcus xylosus DMB3-Bh1 DMB...      97  3e-18
ref|NZ_AURW01000001.1| Staphylococcus xylosus DMB3-Bh1 DMB...      94  3e-17

 Staphylococcus capitis QN1 [firmicutes] taxid 1189311
ref|NZ_AJTH01000004.1| Staphylococcus capitis QN1 Contig3_...     116  8e-25
ref|NZ_AJTH01000012.1| Staphylococcus capitis QN1 Contig16...     112  2e-23
ref|NZ_AJTH01000001.1| Staphylococcus capitis QN1 Contig1,...     102  8e-20

 Staphylococcus epidermidis BVS058A4 [firmicutes] taxid 883130
ref|NZ_KB373336.1| Staphylococcus epidermidis BVS058A4 sup...     116  8e-25
ref|NZ_KB373335.1| Staphylococcus epidermidis BVS058A4 sup...     112  2e-23

 Staphylococcus epidermidis 14.1.R1.SE [firmicutes] taxid 1000590
ref|NZ_AGUC01000011.1| Staphylococcus epidermidis 14.1.R1....     116  9e-25
ref|NZ_AGUC01000099.1| Staphylococcus epidermidis 14.1.R1....      99  6e-19
ref|NZ_AGUC01000069.1| Staphylococcus epidermidis 14.1.R1....      98  8e-19

 Actinomadura oligospora ATCC 43269 [high GC Gram+] taxid 1399798
ref|NZ_JADG01000029.1| Actinomadura oligospora ATCC 43269 ...     115  2e-24

 Streptomyces sp. NRRL S-920 [high GC Gram+] taxid 1463921
ref|NZ_JODF01000004.1| Streptomyces sp. NRRL S-920 contig4...     115  2e-24

 Staphylococcus sp. TE8 [firmicutes] taxid 1472720
ref|NZ_JMGB01000001.1| Staphylococcus sp. TE8 Scaffold01, ...     115  2e-24
ref|NZ_JMGB01000002.1| Staphylococcus sp. TE8 Scaffold02, ...     107  1e-21

 Staphylococcus epidermidis NIHLM095 [firmicutes] taxid 979199
ref|NZ_AKGI01000013.1| Staphylococcus epidermidis NIHLM095...     115  2e-24
ref|NZ_AKGI01000003.1| Staphylococcus epidermidis NIHLM095...     114  6e-24
ref|NZ_AKGI01000011.1| Staphylococcus epidermidis NIHLM095...     100  2e-19

 Staphylococcus epidermidis NIHLM087 [firmicutes] taxid 979201
ref|NZ_AKGK01000058.1| Staphylococcus epidermidis NIHLM087...     115  2e-24
ref|NZ_AKGK01000003.1| Staphylococcus epidermidis NIHLM087...     114  6e-24
ref|NZ_AKGK01000012.1| Staphylococcus epidermidis NIHLM087...     100  2e-19

 Staphylococcus chromogenes MU 970 [firmicutes] taxid 1220551
ref|NZ_JMJF01000020.1| Staphylococcus chromogenes MU 970 S...     115  3e-24
```

```
 Staphylococcus epidermidis AG42 [firmicutes] taxid 1458470
ref|NZ_JNLI01000008.1| Staphylococcus epidermidis AG42 BO1...     115  3e-24
ref|NZ_JNLI01000004.1| Staphylococcus epidermidis AG42 BO1...     114  4e-24

 Halobacillus halophilus DSM 2266 [firmicutes] taxid 866895
ref|NC_017668.1| Halobacillus halophilus DSM 2266, complet...     115  3e-24

 Staphylococcus epidermidis Scl31 [firmicutes] taxid 1344991
ref|NZ_ATDD02000087.1| Staphylococcus epidermidis Scl31 CO...     114  4e-24
ref|NZ_ATDD02000070.1| Staphylococcus epidermidis Scl31 CO...     112  2e-23
ref|NZ_ATDD02000013.1| Staphylococcus epidermidis Scl31 CO...     100  2e-19

 Staphylococcus epidermidis VCU126 [firmicutes] taxid 904342
ref|NZ_AHLG01000070.1| Staphylococcus epidermidis VCU126 c...     114  4e-24
ref|NZ_AHLG01000018.1| Staphylococcus epidermidis VCU126 c...     112  2e-23
ref|NZ_AHLG01000050.1| Staphylococcus epidermidis VCU126 c...     100  2e-19

 Staphylococcus epidermidis VCU111 [firmicutes] taxid 904331
ref|NZ_JHUB01000018.1| Staphylococcus epidermidis VCU111 c...     114  4e-24
ref|NZ_JHUB01000050.1| Staphylococcus epidermidis VCU111 c...     112  3e-23
ref|NZ_JHUB01000017.1| Staphylococcus epidermidis VCU111 c...     100  2e-19

 Staphylococcus epidermidis VCU117 [firmicutes] taxid 904335
ref|NZ_AHLA01000081.1| Staphylococcus epidermidis VCU117 c...     114  4e-24
ref|NZ_AHLA01000020.1| Staphylococcus epidermidis VCU117 c...     112  2e-23
ref|NZ_AHLA01000054.1| Staphylococcus epidermidis VCU117 c...     100  2e-19

 Staphylococcus epidermidis NIH05003 [firmicutes] taxid 1155133
ref|NZ_AKHI01000030.1| Staphylococcus epidermidis NIH05003...     114  4e-24
ref|NZ_AKHI01000007.1| Staphylococcus epidermidis NIH05003...     112  2e-23
ref|NZ_AKHI01000013.1| Staphylococcus epidermidis NIH05003...     100  2e-19

 Staphylococcus epidermidis NIHLM020 [firmicutes] taxid 979213
ref|NZ_AKGW01000039.1| Staphylococcus epidermidis NIHLM020...     114  4e-24
ref|NZ_AKGW01000037.1| Staphylococcus epidermidis NIHLM020...     112  2e-23
ref|NZ_AKGW01000034.1| Staphylococcus epidermidis NIHLM020...     100  2e-19

 Staphylococcus epidermidis NIH051668 [firmicutes] taxid 1155131
ref|NZ_AKHK01000027.1| Staphylococcus epidermidis NIH05166...     114  4e-24
ref|NZ_AKHK01000005.1| Staphylococcus epidermidis NIH05166...     112  2e-23
ref|NZ_AKHK01000012.1| Staphylococcus epidermidis NIH05166...     100  2e-19

 Staphylococcus epidermidis NIHLM039 [firmicutes] taxid 979209
ref|NZ_AKGS01000047.1| Staphylococcus epidermidis NIHLM039...     114  4e-24
ref|NZ_AKGS01000040.1| Staphylococcus epidermidis NIHLM039...     112  2e-23
ref|NZ_AKGS01000035.1| Staphylococcus epidermidis NIHLM039...     101  1e-19

 Staphylococcus epidermidis VCU105 [firmicutes] taxid 904328
ref|NZ_AFTZ01000009.1| Staphylococcus epidermidis VCU105 c...     114  4e-24
ref|NZ_AFTZ01000021.1| Staphylococcus epidermidis VCU105 c...     112  2e-23
ref|NZ_AFTZ01000005.1| Staphylococcus epidermidis VCU105 c...     101  1e-19

 Staphylococcus epidermidis VCU120 [firmicutes] taxid 904337
ref|NZ_AHLC01000004.1| Staphylococcus epidermidis VCU120 c...     114  4e-24
ref|NZ_AHLC01000048.1| Staphylococcus epidermidis VCU120 c...     112  2e-23
ref|NZ_AHLC01000008.1| Staphylococcus epidermidis VCU120 c...     100  2e-19

 Staphylococcus epidermidis NIHLM067 [firmicutes] taxid 979203
ref|NZ_AKGM01000031.1| Staphylococcus epidermidis NIHLM067...     114  4e-24
ref|NZ_AKGM01000009.1| Staphylococcus epidermidis NIHLM067...     109  2e-22
ref|NZ_AKGM01000026.1| Staphylococcus epidermidis NIHLM067...     100  2e-19

 Staphylococcus epidermidis NIHLM018 [firmicutes] taxid 979215
ref|NZ_AKGY01000034.1| Staphylococcus epidermidis NIHLM018...     114  4e-24
ref|NZ_AKGY01000003.1| Staphylococcus epidermidis NIHLM018...     109  2e-22
ref|NZ_AKGY01000033.1| Staphylococcus epidermidis NIHLM018...     100  2e-19

 Staphylococcus epidermidis NIH05005 [firmicutes] taxid 979220
ref|NZ_AKHD01000034.1| Staphylococcus epidermidis NIH05005...     114  4e-24
ref|NZ_AKHD01000044.1| Staphylococcus epidermidis NIH05005...     109  2e-22
ref|NZ_AKHD01000033.1| Staphylococcus epidermidis NIH05005...     100  2e-19

 Staphylococcus epidermidis VCU050 [firmicutes] taxid 904322
ref|NZ_JHQC01000001.1| Staphylococcus epidermidis VCU050 c...     114  4e-24
ref|NZ_JHQC01000002.1| Staphylococcus epidermidis VCU050 c...     112  2e-23
ref|NZ_JHQC01000034.1| Staphylococcus epidermidis VCU050 c...     100  2e-19

 Staphylococcus epidermidis Scl25 [firmicutes] taxid 1344992
ref|NZ_ATDC02000127.1| Staphylococcus epidermidis Scl25 CO...     114  4e-24
ref|NZ_ATDC02000013.1| Staphylococcus epidermidis Scl25 CO...     112  2e-23

 Staphylococcus epidermidis VCU109 [firmicutes] taxid 904330
ref|NZ_AFUA01000010.1| Staphylococcus epidermidis VCU109 c...     114  4e-24
ref|NZ_AFUA01000052.1| Staphylococcus epidermidis VCU109 c...     112  2e-23
ref|NZ_AFUA01000023.1| Staphylococcus epidermidis VCU109 c...     100  2e-19

 Staphylococcus epidermidis VCU081 [firmicutes] taxid 904326
ref|NZ_AHLU01000072.1| Staphylococcus epidermidis VCU081 c...     114  4e-24
ref|NZ_AHLU01000078.1| Staphylococcus epidermidis VCU081 c...     109  3e-22
ref|NZ_AHLU01000049.1| Staphylococcus epidermidis VCU081 c...     100  2e-19

 Staphylococcus epidermidis VCU036 [firmicutes] taxid 904318
ref|NZ_JHUA01000023.1| Staphylococcus epidermidis VCU036 c...     114  4e-24
ref|NZ_JHUA01000034.1| Staphylococcus epidermidis VCU036 c...     112  2e-23
ref|NZ_JHUA01000027.1| Staphylococcus epidermidis VCU036 c...     100  2e-19

 Staphylococcus epidermidis AU12-03 [firmicutes] taxid 1220510
ref|NZ_AMCS01000033.1| Staphylococcus epidermidis AU12-03 ...     114  4e-24
ref|NZ_AMCS01000019.1| Staphylococcus epidermidis AU12-03 ...     112  2e-23
ref|NZ_AMCS01000011.1| Staphylococcus epidermidis AU12-03 ...     100  2e-19

 Staphylococcus epidermidis VCU028 [firmicutes] taxid 904317
ref|NZ_AFEH01000012.1| Staphylococcus epidermidis VCU028 c...     114  4e-24
ref|NZ_AFEH01000021.1| Staphylococcus epidermidis VCU028 c...     109  3e-22
ref|NZ_AFEH01000025.1| Staphylococcus epidermidis VCU028 c...     100  2e-19

 Staphylococcus epidermidis NIH051475 [firmicutes] taxid 1155130
ref|NZ_AKHL01000038.1| Staphylococcus epidermidis NIH05147...     114  5e-24
```

```
ref|NZ_AKHL01000007.1| Staphylococcus epidermidis NIH05147...     112  2e-23
ref|NZ_AKHL01000017.1| Staphylococcus epidermidis NIH05147...     100  2e-19

 Staphylococcus epidermidis SK135 [firmicutes] taxid 596317
ref|NZ_ADEY01000032.1| Staphylococcus epidermidis SK135 ct...     114  5e-24
ref|NZ_ADEY01000017.1| Staphylococcus epidermidis SK135 ct...     112  2e-23
ref|NZ_ADEY01000021.1| Staphylococcus epidermidis SK135 ct...     100  2e-19

 Staphylococcus epidermidis BCM-HMP0060 [firmicutes] taxid 525374
ref|NZ_GG696727.1| Staphylococcus epidermidis BCM-HMP0060 ...     114  5e-24
ref|NZ_GG696728.1| Staphylococcus epidermidis BCM-HMP0060 ...     112  2e-23

 Bacillus simplex BA2H3 [firmicutes] taxid 1403313
ref|NZ_KN360955.1| Bacillus simplex BA2H3 scaffold2, whole...     114  5e-24

 Streptomyces sp. NRRL S-146 [high GC Gram+] taxid 1463884
ref|NZ_JOAW01000001.1| Streptomyces sp. NRRL S-146 contig1...     113  1e-23

 Staphylococcus lugdunensis VCU148 [firmicutes] taxid 904352
ref|NZ_JIBR01000024.1| Staphylococcus lugdunensis VCU148 c...     113  1e-23
ref|NZ_JIBR01000003.1| Staphylococcus lugdunensis VCU148 c...     101  1e-19
ref|NZ_JIBR01000051.1| Staphylococcus lugdunensis VCU148 c...      83  1e-13

 Staphylococcus lugdunensis VCU139 [firmicutes] taxid 904346
ref|NZ_AHLK01000062.1| Staphylococcus lugdunensis VCU139 c...     113  1e-23
ref|NZ_AHLK01000016.1| Staphylococcus lugdunensis VCU139 c...     101  1e-19
ref|NZ_AHLK01000036.1| Staphylococcus lugdunensis VCU139 c...      83  1e-13

 Staphylococcus lugdunensis UCIM6116 [firmicutes] taxid 1409679
ref|NZ_KI978386.1| Staphylococcus lugdunensis UCIM6116 adp...     113  1e-23
ref|NZ_KI978373.1| Staphylococcus lugdunensis UCIM6116 adp...     101  1e-19
ref|NZ_KI978419.1| Staphylococcus lugdunensis UCIM6116 adp...      83  1e-13

 Staphylococcus xylosus [firmicutes] taxid 1288
ref|NZ_JXAU01000021.1| Staphylococcus xylosus strain LSR_0...     112  1e-23
ref|NZ_JXAU01000013.1| Staphylococcus xylosus strain LSR_0...      97  4e-18
ref|NZ_JXAU01000001.1| Staphylococcus xylosus strain LSR_0...      93  3e-17

 Thalassobacter arenae DSM 19593 [a-proteobacteria] taxid 1123360
ref|NZ_KE557312.1| Thalassobacter arenae DSM 19593 Contig3...     112  2e-23

 Staphylococcus lugdunensis ACS-027-V-Sch2 [firmicutes] taxid 883164
ref|NZ_KB373324.1| Staphylococcus lugdunensis ACS-027-V-Sc...     112  2e-23
ref|NZ_KB373322.1| Staphylococcus lugdunensis ACS-027-V-Sc...     101  1e-19

 Staphylococcus lugdunensis VCU150 [firmicutes] taxid 904354
ref|NZ_JIBS01000010.1| Staphylococcus lugdunensis VCU150 c...     112  2e-23
ref|NZ_JIBS01000009.1| Staphylococcus lugdunensis VCU150 c...     101  1e-19
ref|NZ_JIBS01000023.1| Staphylococcus lugdunensis VCU150 c...      83  8e-14

 Staphylococcus lugdunensis M23590 [firmicutes] taxid 525377
ref|NZ_GL622351.1| Staphylococcus lugdunensis M23590 SCAFF...     112  2e-23
ref|NZ_GL622352.1| Staphylococcus lugdunensis M23590 SCAFF...      83  9e-14
```

```
 Staphylococcus lugdunensis N920143 [firmicutes] taxid 1034809
ref|NC_017353.1| Staphylococcus lugdunensis N920143, compl...     112  2e-23

 Staphylococcus lugdunensis HKU09-01 [firmicutes] taxid 698737
ref|NC_013893.1| Staphylococcus lugdunensis HKU09-01 chrom...     112  2e-23

 Staphylococcus hominis subsp. hominis ZBW5 [firmicutes] taxid 1185421
ref|NZ_AKGC01000001.1| Staphylococcus hominis subsp. homin...     112  2e-23
ref|NZ_AKGC01000039.1| Staphylococcus hominis subsp. homin...     105  6e-21
ref|NZ_AKGC01000024.1| Staphylococcus hominis subsp. homin...      97  3e-18

 Staphylococcus sp. M0480 [firmicutes] taxid 1388318
ref|NZ_KK013382.1| Staphylococcus sp. M0480 adAFb-supercon...     112  2e-23
ref|NZ_KK013415.1| Staphylococcus sp. M0480 adAFb-supercon...     105  6e-21
ref|NZ_KK013416.1| Staphylococcus sp. M0480 adAFb-supercon...      96  2e-18

 Staphylococcus epidermidis E13A [firmicutes] taxid 1115805
ref|NZ_AURC01000055.1| Staphylococcus epidermidis E13A ctg...     112  3e-23
ref|NZ_AURC01000046.1| Staphylococcus epidermidis E13A ctg...     111  5e-23
ref|NZ_AURC01000001.1| Staphylococcus epidermidis E13A ctg...     100  2e-19

 Staphylococcus equorum UMC-CNS-924 [firmicutes] taxid 1357294
ref|NZ_AVBD01000010.1| Staphylococcus equorum UMC-CNS-924 ...     111  3e-23
ref|NZ_AVBD01000002.1| Staphylococcus equorum UMC-CNS-924 ...      98  1e-18

 Oceanobacillus iheyensis HTE831 [firmicutes] taxid 221109
ref|NC_004193.1| Oceanobacillus iheyensis HTE831 chromosom...     111  5e-23

 Bacillus phage CampHawk [viruses] taxid 1406783
ref|NC_022761.1| Bacillus phage CampHawk, complete genome         111  5e-23

 Staphylococcus sp. OJ82 [firmicutes] taxid 1202667
ref|NZ_ALPU01000001.1| Staphylococcus sp. OJ82 155.SOJ.1_1...     110  6e-23
ref|NZ_ALPU01000010.1| Staphylococcus sp. OJ82 155.SOJ.1_1...      97  3e-18

 Halobacillus karajensis [firmicutes] taxid 195088
ref|NZ_CCDI010000004.1| Halobacillus karajensis strain HD-...     110  7e-23

 Halobacillus dabanensis [firmicutes] taxid 240302
ref|NZ_CCDH010000002.1| Halobacillus dabanensis strain HD-...     110  7e-23

 Halobacillus trueperi [firmicutes] taxid 156205
ref|NZ_CCDJ010000003.1| Halobacillus trueperi strain HT-01...     110  7e-23

 Desulfotomaculum alcoholivorax DSM 16058 [firmicutes] taxid 1121422
ref|NZ_AUMW01000010.1| Desulfotomaculum alcoholivorax DSM ...     110  1e-22

 Desulfobulbus sp. Tol-SR [d-proteobacteria] taxid 1536652
ref|NZ_JROS01000055.1| Desulfobulbus sp. Tol-SR contig_163...     110  1e-22

 Bradyrhizobium sp. CCBAU 43298 [a-proteobacteria] taxid 1128178
ref|NZ_AJQE01000370.1| Bradyrhizobium sp. CCBAU 43298 Scaf...     109  2e-22

 Dinoroseobacter shibae DFL 12 = DSM 16493 [a-proteobacteria] taxid 398580
```

ref|NC_009952.1| Dinoroseobacter shibae DFL 12 chromosome,... 108 4e-22

Staphylococcus hominis VCU122 [firmicutes] taxid 904339
ref|NZ_AHLD01000066.1| Staphylococcus hominis VCU122 conti... 108 5e-22
ref|NZ_AHLD01000042.1| Staphylococcus hominis VCU122 conti... 103 2e-20

Staphylococcus hominis subsp. hominis C80 [firmicutes] taxid 435837
ref|NZ_GL545260.1| Staphylococcus hominis subsp. hominis C... 108 5e-22

Staphylococcus simulans ACS-120-V-Sch1 [firmicutes] taxid 883166
ref|NZ_KB373326.1| Staphylococcus simulans ACS-120-V-Sch1 ... 108 5e-22

Staphylococcus simulans UMC-CNS-990 [firmicutes] taxid 1405498
ref|NZ_AXDY01000001.1| Staphylococcus simulans UMC-CNS-990... 108 6e-22

Sulfitobacter sp. 20_GPM-1509m [a-proteobacteria] taxid 1380367
ref|NZ_JIBC01000007.1| Sulfitobacter sp. 20_GPM-1509m N517... 108 7e-22

Desulfobacterium autotrophicum HRM2 [d-proteobacteria] taxid 177437
ref|NC_012108.1| Desulfobacterium autotrophicum HRM2 chrom... 107 1e-21

Desulfatirhabdium butyrativorans DSM 18734 [d-proteobacteria] taxid 1121394
ref|NZ_AUCU01000015.1| Desulfatirhabdium butyrativorans DS... 107 1e-21

Rhodocyclaceae bacterium RZ94 [b-proteobacteria] taxid 998429
ref|NZ_KB910520.1| Rhodocyclaceae bacterium RZ94 B594DRAFT... 106 2e-21

Methyloversatilis sp. FAM1 [b-proteobacteria] taxid 1119528
ref|NZ_AZUP01000001.1| Methyloversatilis sp. FAM1 MetFAM1D... 106 2e-21

Methyloversatilis sp. RZ18-153 [b-proteobacteria] taxid 983954
ref|NZ_ARVV01000001.1| Methyloversatilis sp. RZ18-153 MetR... 105 3e-21

Staphylococcus sp. AL1 [firmicutes] taxid 861530
ref|NZ_ALOZ01000075.1| Staphylococcus sp. AL1 contig16, wh... 105 4e-21
ref|NZ_ALOZ01000069.1| Staphylococcus sp. AL1 contig18, wh... 100 3e-19
ref|NZ_ALOZ01000061.1| Staphylococcus sp. AL1 contig48, wh... 95 1e-17

Staphylococcus agnetis [firmicutes] taxid 985762
ref|NZ_JPRT01000004.1| Staphylococcus agnetis strain CBMRN... 105 4e-21

Syntrophobacter fumaroxidans MPOB [d-proteobacteria] taxid 335543
ref|NC_008554.1| Syntrophobacter fumaroxidans MPOB chromos... 105 4e-21

Staphylococcus caprae M23864:W1 [firmicutes] taxid 525378
ref|NZ_GG696773.1| Staphylococcus caprae M23864:W1 SCAFFOL... 105 6e-21
ref|NZ_GG696774.1| Staphylococcus caprae M23864:W1 SCAFFOL... 103 2e-20

Alcanivorax pacificus W11-5 [g-proteobacteria] taxid 391936
SeqId not found Title was not found 104 1e-20

Nocardiopsis salina YIM 90010 [high GC Gram+] taxid 1246485
ref|NZ_ANBF01000005.1| Nocardiopsis salina YIM 90010 conti... 104 1e-20

Agrobacterium rhizogenes [a-proteobacteria] taxid 359
ref|NZ_JQJW01000006.1| Agrobacterium rhizogenes strain YR1... 103 2e-20
ref|NZ_JFZP01000031.1| Agrobacterium rhizogenes strain ATC... 88 2e-15

Oceanobacillus kimchii X50 [firmicutes] taxid 1238184
ref|NZ_CM001792.1| Oceanobacillus kimchii X50 chromosome, ... 103 2e-20

Streptomyces sp. NRRL WC-3773 [high GC Gram+] taxid 1463936
ref|NZ_JOJI01000053.1| Streptomyces sp. NRRL WC-3773 conti... 102 5e-20
ref|NZ_JOJI01000049.1| Streptomyces sp. NRRL WC-3773 conti... 67 1e-08

Staphylococcus saprophyticus subsp. saprophyticus KACC 16562 [firmicutes] taxid 1131257
ref|NZ_AHKB01000037.1| Staphylococcus saprophyticus subsp.... 102 6e-20
ref|NZ_AHKB01000038.1| Staphylococcus saprophyticus subsp.... 100 3e-19
ref|NZ_AHKB01000017.1| Staphylococcus saprophyticus subsp.... 96 4e-18

Sulfitobacter sp. CB2047 [a-proteobacteria] taxid 1525218
ref|NZ_JPOY01000011.1| Sulfitobacter sp. CB2047 contig_3, ... 101 1e-19

Staphylococcus saprophyticus subsp. saprophyticus ATCC 15305 [firmicutes] taxid 342451
ref|NC_007350.1| Staphylococcus saprophyticus subsp. sapro... 100 2e-19

Sulfitobacter sp. EE-36 [a-proteobacteria] taxid 52598
ref|NZ_CH959310.1| Sulfitobacter sp. EE-36 scf_10994513180... 100 2e-19

Desulfosporosinus orientis DSM 765 [firmicutes] taxid 768706
ref|NC_016584.1| Desulfosporosinus orientis DSM 765 chromo... 100 4e-19

Bacillus sp. FJAT-13831 [firmicutes] taxid 1150157
ref|NZ_JH921526.1| Bacillus sp. FJAT-13831 Scaffold19, who... 98 7e-19
ref|NZ_JH921531.1| Bacillus sp. FJAT-13831 Scaffold29, who... 96 4e-18

Staphylococcus sp. E463 [firmicutes] taxid 1234593
ref|NZ_ANBY01000062.1| Staphylococcus sp. E463 contig00062... 98 7e-19
ref|NZ_ANBY01000098.1| Staphylococcus sp. E463 contig00101... 89 1e-15

Thermoactinomyces sp. Gus2-1 [firmicutes] taxid 1535750
ref|NZ_JPZM01000032.1| Thermoactinomyces sp. Gus2-1 c38, w... 98 1e-18

Dactylosporangium aurantiacum [high GC Gram+] taxid 35754
ref|NZ_JNYJ01000002.1| Dactylosporangium aurantiacum strai... 98 1e-18

Bacillus phage SPO1 [viruses] taxid 10685
ref|NC_011421.1| Bacillus phage SPO1, complete genome 98 1e-18

Sulfitobacter pontiacus 3SOLIMAR09 [a-proteobacteria] taxid 1406866
ref|NZ_AXZR01000001.1| Sulfitobacter pontiacus 3SOLIMAR09 ... 98 1e-18

Acinetobacter baumannii EGD-HP18 [g-proteobacteria] taxid 1358412
ref|NZ_AVST01000007.1| Acinetobacter baumannii EGD-HP18 co... 93 1e-18

Rhizobium tropici CIAT 899 [a-proteobacteria] taxid 698761

```
ref|NC_020059.1| Rhizobium tropici CIAT 899, complete genome        98  1e-18

 Lentzea albidocapillata [high GC Gram+] taxid 40571
ref|NZ_JOEA01000003.1| Lentzea albidocapillata strain NRRL...      97  2e-18

 Methyloversatilis universalis EHg5 [b-proteobacteria] taxid 999628
ref|NZ_KB900539.1| Methyloversatilis universalis EHg5 Metu...      97  2e-18

 Bradyrhizobium elkanii WSM2783 [a-proteobacteria] taxid 1038860
ref|NZ_AXAP01000055.1| Bradyrhizobium elkanii WSM2783 YY7D...      97  3e-18

 Intestinibacter bartlettii DORA_8_9 [firmicutes] taxid 1403940
ref|NZ_AZLW01000181.1| Intestinibacter bartlettii DORA_8_9...      95  3e-18

 Alkalilimnicola ehrlichii MLHE-1 [g-proteobacteria] taxid 187272
ref|NC_008340.1| Alkalilimnicola ehrlichii MLHE-1 chromoso...      96  4e-18

 Sulfitobacter sp. NAS-14.1 [a-proteobacteria] taxid 314267
ref|NZ_CH959312.1| Sulfitobacter sp. NAS-14.1 scf_10994513...      96  5e-18

 Mesorhizobium sp. WSM3224 [a-proteobacteria] taxid 1040986
ref|NZ_ATYO01000001.1| Mesorhizobium sp. WSM3224 YU3DRAFT_...      96  6e-18

 Thauera sp. MZ1T [b-proteobacteria] taxid 85643
ref|NC_011662.2| Thauera sp. MZ1T chromosome, complete genome      96  7e-18

 Desulfosporosinus youngiae DSM 17734 [firmicutes] taxid 768710
ref|NZ_CM001441.1| Desulfosporosinus youngiae DSM 17734 ch...      95  7e-18

 Methylotenera sp. 1P/1 [b-proteobacteria] taxid 1131551
ref|NZ_ARWC01000001.1| Methylotenera sp. 1P/1 A3Q3DRAFT_ch...      95  8e-18

 Pseudomonas fluorescens F113 [g-proteobacteria] taxid 1114970
ref|NC_016830.1| Pseudomonas fluorescens F113 chromosome, ...      95  8e-18

 Streptomyces sp. AW19M42 [high GC Gram+] taxid 1379686
ref|NZ_CBRG010000068.1| Streptomyces sp. AW19M42, whole ge...      95  1e-17

 Methyloversatilis sp. NVD [b-proteobacteria] taxid 1117409
ref|NZ_KB889967.1| Methyloversatilis sp. NVD A3Q7DRAFT_sca...      95  1e-17

 Intestinibacter bartlettii DSM 16795 [firmicutes] taxid 445973
ref|NZ_DS499566.1| Intestinibacter bartlettii DSM 16795 Sc...      95  1e-17

 Desulfospira joergensenii DSM 10085 [d-proteobacteria] taxid 1265505
ref|NZ_ATUG01000001.1| Desulfospira joergensenii DSM 10085...      95  1e-17

 Methylocystis sp. ATCC 49242 [a-proteobacteria] taxid 622637
ref|NZ_KE124774.1| Methylocystis sp. ATCC 49242 strain Roc...      95  2e-17

 Pseudomonas aeruginosa [g-proteobacteria] taxid 287
ref|NZ_JTYC01000012.1| Pseudomonas aeruginosa strain AZPAE...      95  2e-17

 Desulfotomaculum gibsoniae DSM 7213 [firmicutes] taxid 767817
ref|NC_021184.1| Desulfotomaculum gibsoniae DSM 7213, comp...      94  2e-17

 Mesorhizobium sp. LNJC384A00 [a-proteobacteria] taxid 1287268
ref|NZ_AYWK01000020.1| Mesorhizobium sp. LNJC384A00 scaffo...      94  3e-17

 Sulfitobacter sp. NB-68 [a-proteobacteria] taxid 1342302
ref|NZ_JASC01000002.1| Sulfitobacter sp. NB-68 NB68_contig...      94  3e-17

 Spirochaeta sp. JC202 [spirochetes] taxid 1329640
ref|NZ_JRAS01000047.1| Spirochaeta sp. JC202, whole genome...      93  3e-17

 Streptosporangium roseum [high GC Gram+] taxid 2001
ref|NZ_JOEP01000042.1| Streptosporangium roseum strain NRR...      93  4e-17

 Streptacidiphilus oryzae TH49 [high GC Gram+] taxid 1449353
ref|NZ_JQMQ01000005.1| Streptacidiphilus oryzae TH49 BS73D...      93  6e-17

 Streptomyces sp. NRRL F-6131 [high GC Gram+] taxid 1463874
ref|NZ_JOHN01000051.1| Streptomyces sp. NRRL F-6131 contig...      93  7e-17

 Streptomyces viridochromogenes Tue57 [high GC Gram+] taxid 1160705
ref|NZ_AMLP01000052.1| Streptomyces viridochromogenes Tue5...      92  1e-16

 Comamonas badia DSM 17552 [b-proteobacteria] taxid 1121348
ref|NZ_AXVM01000001.1| Comamonas badia DSM 17552 K320DRAFT...      92  2e-16

 Rhizobium sp. JGI 001013-F22 [a-proteobacteria] taxid 1167748
ref|NZ_AUSB02000045.1| Rhizobium sp. JGI 001013-F22 C601DR...      90  4e-16

 Methylocystis parvus OBBP [a-proteobacteria] taxid 1134912
ref|NZ_AJTV01000010.1| Methylocystis parvus OBBP contig010...      90  5e-16

 Mesorhizobium australicum WSM2073 [a-proteobacteria] taxid 754035
ref|NC_019973.1| Mesorhizobium australicum WSM2073, comple...      90  5e-16

 Streptomyces sp. CNS654 [high GC Gram+] taxid 1506995
ref|NZ_JNLT01000036.1| Streptomyces sp. CNS654 CD02DRAFT_s...      90  5e-16

 Saccharopolyspora erythraea D [high GC Gram+] taxid 1382595
ref|NZ_AVCN01000092.1| Saccharopolyspora erythraea D scaff...      89  8e-16

 Pseudomonas aeruginosa JD325 [g-proteobacteria] taxid 1403042
ref|NZ_AWYY01002044.1| Pseudomonas aeruginosa JD325, whole...      89  8e-16

 Saccharopolyspora erythraea NRRL 2338 [high GC Gram+] taxid 405948
ref|NZ_ABFV01000070.1| Saccharopolyspora erythraea NRRL 23...      89  8e-16
ref|NC_009142.1| Saccharopolyspora erythraea NRRL 2338 chr...      89  8e-16

 Desulfotomaculum ruminis DSM 2154 [firmicutes] taxid 696281
ref|NC_015589.1| Desulfotomaculum ruminis DSM 2154 chromos...      90  8e-16

 Burkholderia phenoliruptrix AC1100 [b-proteobacteria] taxid 1337996
ref|NZ_ASXI01000125.1| Burkholderia phenoliruptrix AC1100 ...      89  8e-16
```

```
 Pantholops hodgsonii [even-toed ungulates] taxid 59538
ref|NW_005814045.1| Pantholops hodgsonii unplaced genomic ...      89  9e-16

 Mesorhizobium sp. LSJC255A00 [a-proteobacteria] taxid 1287313
ref|NZ_AYVR01000003.1| Mesorhizobium sp. LSJC255A00 scaffo...      89  1e-15

 Mesorhizobium sp. LSHC414A00 [a-proteobacteria] taxid 1287287
ref|NZ_AYWA01000002.1| Mesorhizobium sp. LSHC414A00 scaffo...      89  1e-15

 Mesorhizobium loti USDA 3471 [a-proteobacteria] taxid 1040983
ref|NZ_AXAE01000018.1| Mesorhizobium loti USDA 3471 A3AUDR...      89  1e-15

 Agrobacterium radiobacter K84 [a-proteobacteria] taxid 311403
ref|NC_011985.1| Agrobacterium radiobacter K84 chromosome ...      88  2e-15
ref|NC_011983.1| Agrobacterium radiobacter K84 chromosome ...      86  1e-14

 Thermoactinomyces daqus [firmicutes] taxid 1329516
ref|NZ_JPST01000012.1| Thermoactinomyces daqus strain H-18...      88  3e-15

 Streptomyces purpeofuscus [high GC Gram+] taxid 67352
ref|NZ_JODS01000012.1| Streptomyces purpeofuscus strain NR...      88  3e-15

 Rhizobium sp. YR060 [a-proteobacteria] taxid 1500309
ref|NZ_JQLB01000002.1| Rhizobium sp. YR060 EX03DRAFT_scaff...      87  4e-15

 Pectobacterium sp. SCC3193 [enterobacteria] taxid 1166016
ref|NC_017845.1| Pectobacterium sp. SCC3193, complete genome       87  4e-15

 Actinoplanes friuliensis DSM 7358 [high GC Gram+] taxid 1246995
ref|NC_022657.1| Actinoplanes friuliensis DSM 7358, comple...      87  4e-15

 Streptomyces sp. NRRL B-5680 [high GC Gram+] taxid 1463838
ref|NZ_JOGG01000004.1| Streptomyces sp. NRRL B-5680 contig...      86  1e-14

 Streptomyces sp. e14 [high GC Gram+] taxid 645465
ref|NZ_GG753627.1| Streptomyces sp. e14 genomic scaffold s...      86  1e-14

 Streptomyces sp. Amel2xE9 [high GC Gram+] taxid 1157634
ref|NZ_KB912974.1| Streptomyces sp. Amel2xE9 B065DRAFT_sca...      86  1e-14

 Streptomyces aureofaciens [high GC Gram+] taxid 1894
ref|NZ_JOER01000014.1| Streptomyces aureofaciens strain NR...      86  1e-14
ref|NZ_JNWR01000002.1| Streptomyces aureofaciens strain NR...      82  2e-13

 Streptomyces avellaneus [high GC Gram+] taxid 68178
ref|NZ_JOFK01000001.1| Streptomyces avellaneus strain NRRL...      86  1e-14

 Desulfatiglans anilini DSM 4660 [d-proteobacteria] taxid 1121399
ref|NZ_AULM01000001.1| Desulfatiglans anilini DSM 4660 H56...      86  1e-14

 Desulfitobacterium hafniense TCP-A [firmicutes] taxid 872024
ref|NZ_KB900391.1| Desulfitobacterium hafniense TCP-A Desh...      85  2e-14

 Desulfitobacterium hafniense DP7 [firmicutes] taxid 537010
ref|NZ_JH414435.1| Desulfitobacterium hafniense DP7 Scfld3...      85  2e-14

 Desulfitobacterium hafniense PCP-1 [firmicutes] taxid 1090321
ref|NZ_KB902330.1| Desulfitobacterium hafniense PCP-1 A37Y...      85  2e-14

 Desulfitobacterium hafniense DCB-2 [firmicutes] taxid 272564
ref|NC_011830.1| Desulfitobacterium hafniense DCB-2 chromo...      85  2e-14

 Halomonas elongata DSM 2581 [g-proteobacteria] taxid 768066
ref|NC_014532.1| Halomonas elongata DSM 2581 chromosome, c...      85  2e-14

 Pseudomonas sp. EGD-AK9 [g-proteobacteria] taxid 1386078
ref|NZ_AVOF01000731.1| Pseudomonas sp. EGD-AK9 Contig_741,...      81  2e-14

 Streptomyces sp. NRRL S-244 [high GC Gram+] taxid 1463897
ref|NZ_JOCX01000025.1| Streptomyces sp. NRRL S-244 contig2...      85  3e-14

 Desulfitobacterium hafniense Y51 [firmicutes] taxid 138119
ref|NC_007907.1| Desulfitobacterium hafniense Y51 chromoso...      84  4e-14

 Mesorhizobium sp. LNJC386A00 [a-proteobacteria] taxid 1287270
ref|NZ_AYWJ01000012.1| Mesorhizobium sp. LNJC386A00 scaffo...      84  6e-14

 Streptomyces sp. NRRL F-5702 [high GC Gram+] taxid 1463870
ref|NZ_JOHD01000073.1| Streptomyces sp. NRRL F-5702 contig...      84  6e-14

 Streptomyces purpeochromogenes [high GC Gram+] taxid 1464077
ref|NZ_JODK01000041.1| Streptomyces purpeochromogenes stra...      84  6e-14
ref|NZ_JOBG01000017.1| Streptomyces purpeochromogenes stra...      84  6e-14

 Streptomyces californicus [high GC Gram+] taxid 67281
ref|NZ_KL569912.1| Streptomyces californicus strain NRRL B...      84  6e-14
ref|NZ_KL573626.1| Streptomyces californicus strain NRRL I...      84  6e-14
ref|NZ_JOFG01000015.1| Streptomyces californicus strain NR...      84  6e-14
ref|NZ_JNZZ01000018.1| Streptomyces californicus strain NR...      84  6e-14

 Streptomyces sp. NRRL F-3273 [high GC Gram+] taxid 1463848
ref|NZ_JOIO01000018.1| Streptomyces sp. NRRL F-3273 contig...      84  6e-14

 Streptomyces brasiliensis [high GC Gram+] taxid 1954
ref|NZ_JNXB01000010.1| Streptomyces brasiliensis strain NR...      84  6e-14

 Streptomyces sp. NRRL F-3218 [high GC Gram+] taxid 1463847
ref|NZ_JOIP01000008.1| Streptomyces sp. NRRL F-3218 contig...      84  6e-14

 Streptomyces griseus subsp. rhodochrous [high GC Gram+] taxid 285575
ref|NZ_JOFF01000020.1| Streptomyces griseus subsp. rhodoch...      84  6e-14
ref|NZ_JODO01000040.1| Streptomyces griseus subsp. rhodoch...      83  6e-14
ref|NZ_JOFD01000036.1| Streptomyces griseus subsp. rhodoch...      83  6e-14
ref|NZ_JOFE01000014.1| Streptomyces griseus subsp. rhodoch...      83  7e-14

 Streptomyces griseus subsp. griseus [high GC Gram+] taxid 67263
ref|NZ_JOIT01000036.1| Streptomyces griseus subsp. griseus...      83  6e-14
ref|NZ_JOBR01000015.1| Streptomyces griseus subsp. griseus...      77  8e-12
```

```
 Streptomyces puniceus [high GC Gram+] taxid 67351
ref|NZ_JOFA01000020.1| Streptomyces puniceus strain NRRL B...      83  6e-14
ref|NZ_JOBQ01000018.1| Streptomyces puniceus strain NRRL I...      83  6e-14

 Streptomyces sp. NRRL WC-3540 [high GC Gram+] taxid 1463924
ref|NZ_JOCW01000014.1| Streptomyces sp. NRRL WC-3540 conti...      83  7e-14

 Streptomyces sp. SolWspMP-sol2th [high GC Gram+] taxid 1157636
ref|NZ_ARPF01000016.1| Streptomyces sp. SolWspMP-sol2th B0...      83  7e-14

 Desulfitobacterium sp. PCE1 [firmicutes] taxid 146907
ref|NZ_KB913023.1| Desulfitobacterium sp. PCE1 DesPCE1DRAF...      83  1e-13

 Streptomyces sp. NRRL F-2202 [high GC Gram+] taxid 1463839
ref|NZ_JOIH01000016.1| Streptomyces sp. NRRL F-2202 contig...      83  1e-13

 Streptomyces sp. NRRL F-5681 [high GC Gram+] taxid 1463869
ref|NZ_JOHA01000016.1| Streptomyces sp. NRRL F-5681 contig...      83  1e-13

 Methylibium sp. T29-B [b-proteobacteria] taxid 1437443
ref|NZ_AZSN01000056.1| Methylibium sp. T29-B contig000056,...      83  1e-13

 Methylibium sp. T29 [b-proteobacteria] taxid 1430884
ref|NZ_AZND01000034.1| Methylibium sp. T29 contig000034, w...      83  1e-13

 Dasania marina DSM 21967 [g-proteobacteria] taxid 1121374
ref|NZ_KB891589.1| Dasania marina DSM 21967 B067DRAFT_scaf...      83  1e-13

 Streptomyces sp. ScaeMP-e10 [high GC Gram+] taxid 1156841
ref|NZ_KB891297.1| Streptomyces sp. ScaeMP-e10 B061DRAFT_s...      83  1e-13

 Streptomyces sp. NRRL B-1381 [high GC Gram+] taxid 1463829
ref|NZ_JOHG01000065.1| Streptomyces sp. NRRL B-1381 contig...      82  2e-13

 Streptomyces floridae [high GC Gram+] taxid 68201
ref|NZ_JOAC01000025.1| Streptomyces floridae strain NRRL 2...      82  3e-13

 Streptomyces sp. Wigar10 [high GC Gram+] taxid 1085628
ref|NZ_AGDF01000187.1| Streptomyces sp. Wigar10 contig0019...      82  3e-13

 Ochrobactrum anthropi 60a [a-proteobacteria] taxid 1201037
ref|NZ_AMCC01000022.1| Ochrobactrum anthropi 60a Contig22,...      82  3e-13

 Streptomyces alboviridis [high GC Gram+] taxid 67269
ref|NZ_JNWU01000007.1| Streptomyces alboviridis strain NRR...      82  3e-13

 Streptomyces fulvissimus DSM 40593 [high GC Gram+] taxid 1303692
ref|NC_021177.1| Streptomyces fulvissimus DSM 40593 chromo...      82  3e-13

 Streptomyces sp. NRRL S-623 [high GC Gram+] taxid 1463916
ref|NZ_JOJC01000022.1| Streptomyces sp. NRRL S-623 contig2...      82  4e-13

 Streptomyces sp. SA3_actF [high GC Gram+] taxid 682181
ref|NZ_ADXB01000777.1| Streptomyces sp. SA3_actF contig006...      80  4e-13

 Streptomyces cyaneofuscatus [high GC Gram+] taxid 66883
ref|NZ_JOEM01000012.1| Streptomyces cyaneofuscatus strain ...      81  5e-13

 Curvibacter gracilis ATCC BAA-807 [b-proteobacteria] taxid 1336244
ref|NZ_JADZ01000024.1| Curvibacter gracilis ATCC BAA-807 L...      81  6e-13

 Streptomyces sp. W007 [high GC Gram+] taxid 1055352
ref|NZ_AGSW01000067.1| Streptomyces sp. W007 contig00070, ...      80  6e-13

 Streptomyces flavochromogenes [high GC Gram+] taxid 68199
ref|NZ_JNZO01000029.1| Streptomyces flavochromogenes strai...      80  7e-13

 Mesorhizobium sp. LSJC285A00 [a-proteobacteria] taxid 1287338
ref|NZ_AYVK01000014.1| Mesorhizobium sp. LSJC285A00 scaffo...      80  7e-13

 Streptomyces sp. SA3_actG [high GC Gram+] taxid 683219
ref|NZ_ADXA01000111.1| Streptomyces sp. SA3_actG contig000...      80  8e-13

 Streptomyces purpureus KA281 [high GC Gram+] taxid 1054860
ref|NZ_KB913030.1| Streptomyces purpureus KA281 StrpuDRAFT...      80  8e-13

 Streptomyces sp. NRRL F-5135 [high GC Gram+] taxid 1463858
ref|NZ_JOHR01000016.1| Streptomyces sp. NRRL F-5135 contig...      80  9e-13

 Streptomyces sp. CcalMP-8W [high GC Gram+] taxid 1155715
ref|NZ_KB890918.1| Streptomyces sp. CcalMP-8W B053DRAFT_sc...      80  9e-13

 Streptomyces sp. JS01 [high GC Gram+] taxid 1525753
ref|NZ_JPWW01000003.1| Streptomyces sp. JS01 contig3, whol...      80  1e-12

 Mesorhizobium loti MAFF303099 [a-proteobacteria] taxid 266835
ref|NC_002678.2| Mesorhizobium loti MAFF303099 chromosome,...      80  1e-12

 Streptomyces sp. Tu6071 [high GC Gram+] taxid 355249
ref|NZ_CM001165.1| Streptomyces sp. Tu6071 chromosome, who...      79  2e-12

 Streptomyces sp. PCS3-D2 [high GC Gram+] taxid 1460244
ref|NZ_JDUZ01000003.1| Streptomyces sp. PCS3-D2 scaffold00...      79  3e-12

 Streptomyces griseus subsp. griseus NBRC 13350 [high GC Gram+] taxid 455632
ref|NC_010572.1| Streptomyces griseus subsp. griseus NBRC ...      78  3e-12

 Streptomyces griseus XylebKG-1 [high GC Gram+] taxid 649189
ref|NZ_GL877172.1| Streptomyces griseus XylebKG-1 genomic ...      78  4e-12

 Streptomyces sp. SPB78 [high GC Gram+] taxid 591157
ref|NZ_GG657742.1| Streptomyces sp. SPB78 supercont1.1, wh...      50  5e-12

 Streptomyces exfoliatus DSM 41693 [high GC Gram+] taxid 1440059
ref|NZ_AZSS01000324.1| Streptomyces exfoliatus DSM 41693 C...      77  8e-12

 Streptomyces thermolilacinus SPC6 [high GC Gram+] taxid 1306406
```

```
ref|NZ_ASHX01000001.1| Streptomyces thermolilacinus SPC6 c...      77  1e-11

 Streptomyces somaliensis DSM 40738 [high GC Gram+] taxid 1134445
ref|NZ_AJJM01000071.1| Streptomyces somaliensis DSM 40738 ...      76  2e-11

 Streptomyces viridochromogenes DSM 40736 [high GC Gram+] taxid 591159
ref|NZ_GG657757.1| Streptomyces viridochromogenes DSM 4073...      75  5e-11

 Uliginosibacterium gangwonense DSM 18521 [b-proteobacteria] taxid 1123487
ref|NZ_KB892850.1| Uliginosibacterium gangwonense DSM 1852...      75  6e-11

 Lechevalieria aerocolonigenes [high GC Gram+] taxid 68170
ref|NZ_JOFI01000004.1| Lechevalieria aerocolonigenes strai...      74  6e-11

 Streptomyces rimosus subsp. rimosus [high GC Gram+] taxid 132474
ref|NZ_JOBO01000021.1| Streptomyces rimosus subsp. rimosus...      73  2e-10
ref|NZ_JOES01000011.1| Streptomyces rimosus subsp. rimosus...      73  2e-10
ref|NZ_JOBV01000012.1| Streptomyces rimosus subsp. rimosus...      73  2e-10
ref|NZ_JOBW01000006.1| Streptomyces rimosus subsp. rimosus...      72  4e-10
ref|NZ_JNWX01000009.1| Streptomyces rimosus subsp. rimosus...      71  7e-10
ref|NZ_JOBT01000038.1| Streptomyces rimosus subsp. rimosus...      71  7e-10
ref|NZ_JOCR01000003.1| Streptomyces rimosus subsp. rimosus...      71  7e-10
ref|NZ_JOCO01000006.1| Streptomyces rimosus subsp. rimosus...      71  7e-10
ref|NZ_JOET01000005.1| Streptomyces rimosus subsp. rimosus...      71  7e-10
ref|NZ_JNYK01000007.1| Streptomyces rimosus subsp. rimosus...      71  7e-10
ref|NZ_JOEO01000006.1| Streptomyces rimosus subsp. rimosus...      71  7e-10
ref|NZ_JOBN01000004.1| Streptomyces rimosus subsp. rimosus...      71  7e-10
ref|NZ_JOCL01000006.1| Streptomyces rimosus subsp. rimosus...      71  7e-10
ref|NZ_JOBS01000006.1| Streptomyces rimosus subsp. rimosus...      71  7e-10
ref|NZ_JOCP01000003.1| Streptomyces rimosus subsp. rimosus...      71  7e-10
ref|NZ_JNYR01000002.1| Streptomyces rimosus subsp. rimosus...      71  7e-10
ref|NZ_JOJJ01000002.1| Streptomyces rimosus subsp. rimosus...      71  8e-10
ref|NZ_JOBP01000003.1| Streptomyces rimosus subsp. rimosus...      71  8e-10
ref|NZ_JOCQ01000006.1| Streptomyces rimosus subsp. rimosus...      71  9e-10

 Streptomyces sp. NRRL B-11253 [high GC Gram+] taxid 1463826
ref|NZ_JNWN01000006.1| Streptomyces sp. NRRL B-11253 conti...      72  5e-10

 Streptomyces peucetius [high GC Gram+] taxid 1950
ref|NZ_JOCK01000005.1| Streptomyces peucetius strain NRRL ...      72  5e-10

 Streptomyces sp. NRRL WC-3719 [high GC Gram+] taxid 1463932
ref|NZ_JOCD01000055.1| Streptomyces sp. NRRL WC-3719 conti...      72  6e-10

 Streptomyces rimosus subsp. rimosus ATCC 10970 [high GC Gram+] taxid 1265868
ref|NZ_ANSJ01000148.1| Streptomyces rimosus subsp. rimosus...      71  7e-10

 Streptomyces globisporus subsp. globisporus [high GC Gram+] taxid 66858
ref|NZ_JODW01000056.1| Streptomyces globisporus subsp. glo...      71  7e-10

 Streptomyces rimosus [high GC Gram+] taxid 1927
ref|NZ_JMGX01000022.1| Streptomyces rimosus strain R6-500M...      71  7e-10

 Streptomyces sp. NRRL WC-3702 [high GC Gram+] taxid 1463929
ref|NZ_JOCC01000005.1| Streptomyces sp. NRRL WC-3702 conti...      71  7e-10

 Streptomyces capuensis [high GC Gram+] taxid 1464056
ref|NZ_JOFM01000005.1| Streptomyces capuensis strain NRRL ...      71  7e-10
ref|NZ_JNWP01000008.1| Streptomyces capuensis strain NRRL ...      71  7e-10

 Streptomyces sp. NRRL WC-3703 [high GC Gram+] taxid 1463930
ref|NZ_JOCH01000007.1| Streptomyces sp. NRRL WC-3703 conti...      71  7e-10

 Streptomyces lavendulae subsp. lavendulae [high GC Gram+] taxid 58340
ref|NZ_JOEX01000006.1| Streptomyces lavendulae subsp. lave...      71  7e-10
ref|NZ_JOCN01000008.1| Streptomyces lavendulae subsp. lave...      71  7e-10

 Mastigocoleus testarum BC008 [cyanobacteria] taxid 371196
ref|NZ_AXAQ01000291.1| Mastigocoleus testarum BC008 YY1DRA...      70  2e-09

 Rhizobium sp. OK494 [a-proteobacteria] taxid 1500306
ref|NZ_JQLA01000011.1| Rhizobium sp. OK494 EW96DRAFT_scaff...      68  5e-09

 Streptomyces monomycini [high GC Gram+] taxid 371720
ref|NZ_KL571127.1| Streptomyces monomycini strain NRRL B-2...      68  8e-09

 Streptomyces wedmorensis [high GC Gram+] taxid 43759
ref|NZ_JNWK01000040.1| Streptomyces wedmorensis strain NRR...      66  3e-08

 Streptomyces sp. SS [high GC Gram+] taxid 260742
ref|NZ_JH941042.1| Streptomyces sp. SS scaffold2, whole ge...      65  4e-08

 Streptomyces exfoliatus [high GC Gram+] taxid 1905
ref|NZ_JNZP01000010.1| Streptomyces exfoliatus strain NRRL...      65  4e-08
ref|NZ_JOFB01000006.1| Streptomyces exfoliatus strain NRRL...      65  4e-08

 Streptomyces sp. NRRL F-5727 [high GC Gram+] taxid 1463871
ref|NZ_JOGX01000053.1| Streptomyces sp. NRRL F-5727 contig...      65  5e-08

 Ricinus communis [eudicots] taxid 3988
ref|NW_003003203.1| Ricinus communis scf_1106159305140, wh...      64  7e-08

 Streptomyces sclerotialus [high GC Gram+] taxid 1957
ref|NZ_JODX01000043.1| Streptomyces sclerotialus strain NR...      64  8e-08

 Halomonas sp. A3H3 [g-proteobacteria] taxid 1346287
ref|NZ_HG423312.1| Halomonas sp. A3H3 genomic scaffold, HA...      62  3e-07
ref|NZ_HG423343.1| Halomonas sp. A3H3 genomic chromosome, ...      62  3e-07

 Streptomyces sp. CNS615 [high GC Gram+] taxid 1169158
ref|NZ_KB846736.1| Streptomyces sp. CNS615 D583DRAFT_scaff...      62  4e-07

 Streptomyces griseoluteus [high GC Gram+] taxid 29306
ref|NZ_JOBE01000040.1| Streptomyces griseoluteus strain NR...      62  4e-07

 Leptolyngbya boryana PCC 6306 [cyanobacteria] taxid 272134
ref|NZ_KB731325.1| Leptolyngbya boryana PCC 6306 LepboDRAF...      62  6e-07
```

```
 Streptomyces sp. CNR698 [high GC Gram+] taxid 1206101
ref|NZ_AZXC01000002.1| Streptomyces sp. CNR698 B101DRAFT_s...      61  1e-06

 Streptomyces sp. CNT302 [high GC Gram+] taxid 1169155
ref|NZ_KB898274.1| Streptomyces sp. CNT302 D329DRAFT_scaff...      60  2e-06

 Sinorhizobium fredii HH103 [a-proteobacteria] taxid 1117943
ref|NC_016812.1| Sinorhizobium fredii HH103, complete genome       60  2e-06

 Rhizobium sp. PDO1-076 [a-proteobacteria] taxid 1125979
ref|NZ_AHZC01000134.1| Rhizobium sp. PDO1-076 strain PDO-0...      58  9e-06

 Pelobacter seleniigenes DSM 18267 [d-proteobacteria] taxid 1122946
ref|NZ_JOMG01000001.1| Pelobacter seleniigenes DSM 18267 N...      55  9e-05

 Rhodococcus opacus B4 [high GC Gram+] taxid 632772
ref|NC_012522.1| Rhodococcus opacus B4, complete genome            54  1e-04

 marine actinobacterium MedAcidi-G1 [high GC Gram+] taxid 1550399
ref|NZ_JUEM01000014.1| Marine actinobacterium MedAcidi-G1 ...      54  2e-04

 Streptomyces sp. NRRL WC-3725 [high GC Gram+] taxid 1463933
ref|NZ_JOJH01000776.1| Streptomyces sp. NRRL WC-3725 conti...      50  4e-04

 Candidatus Entotheonella sp. TSY1 [d-proteobacteria] taxid 1429438
ref|NZ_KI932132.1| Candidatus Entotheonella sp. TSY1 TSY1_...      52  0.001

 Mycobacterium tuberculosis [high GC Gram+] taxid 1773
ref|NZ_JNVJ01000045.1| Mycobacterium tuberculosis strain V...      52  0.001
ref|NZ_JMJH01000025.1| Mycobacterium tuberculosis strain V...      52  0.001
ref|NZ_JMEK01000009.1| Mycobacterium tuberculosis strain C...      52  0.001

 Mycobacterium tuberculosis M1906 [high GC Gram+] taxid 1448724
ref|NZ_KK330605.1| Mycobacterium tuberculosis M1906 adOYD-...      52  0.001

 Mycobacterium tuberculosis H2760 [high GC Gram+] taxid 1448814
ref|NZ_KK336968.1| Mycobacterium tuberculosis H2760 adPaX-...      52  0.001

 Mycobacterium tuberculosis H3005 [high GC Gram+] taxid 1448816
ref|NZ_KK337115.1| Mycobacterium tuberculosis H3005 adPcv-...      52  0.001

 Mycobacterium tuberculosis H3361 [high GC Gram+] taxid 1448819
ref|NZ_KK337350.1| Mycobacterium tuberculosis H3361 adOVm-...      52  0.001

 Mycobacterium tuberculosis NA-A0009 [high GC Gram+] taxid 1200348
ref|NZ_ALYH01000060.1| Mycobacterium tuberculosis NA-A0009...      52  0.001

 Mycobacterium tuberculosis NA-A0008 [high GC Gram+] taxid 1200347
ref|NZ_ALYG01000020.1| Mycobacterium tuberculosis NA-A0008...      52  0.001

 Mycobacterium tuberculosis M1893 [high GC Gram+] taxid 1448722
ref|NZ_KK330430.1| Mycobacterium tuberculosis M1893 adPda-...      52  0.001

 Mycobacterium tuberculosis M992 [high GC Gram+] taxid 1448615
ref|NZ_KK321455.1| Mycobacterium tuberculosis M992 adPeV-s...      52  0.001

 Mycobacterium tuberculosis 43-16836 [high GC Gram+] taxid 1348776
ref|NZ_ATNF01000041.1| Mycobacterium tuberculosis 43-16836...      52  0.001

 Mycobacterium tuberculosis M1961 [high GC Gram+] taxid 1448735
ref|NZ_KK331391.1| Mycobacterium tuberculosis M1961 adOUE-...      52  0.001

 Mycobacterium tuberculosis PR05 [high GC Gram+] taxid 1279011
ref|NZ_AOMG02000013.1| Mycobacterium tuberculosis PR05 PR0...      52  0.001

 Mycobacterium tuberculosis M1029 [high GC Gram+] taxid 1448630
ref|NZ_JKPK01000012.1| Mycobacterium tuberculosis M1029 ad...      52  0.001

 Mycobacterium tuberculosis H3367 [high GC Gram+] taxid 1448833
ref|NZ_KK338048.1| Mycobacterium tuberculosis H3367 adOYn-...      52  0.001

 Mycobacterium tuberculosis TKK_04_0085 [high GC Gram+] taxid 1448560
ref|NZ_KK320606.1| Mycobacterium tuberculosis TKK_04_0085 ...      52  0.001

 Mycobacterium tuberculosis M1221 [high GC Gram+] taxid 1448637
ref|NZ_KK321937.1| Mycobacterium tuberculosis M1221 adPdn-...      52  0.001

 Mycobacterium tuberculosis M1948 [high GC Gram+] taxid 1448731
ref|NZ_KK331091.1| Mycobacterium tuberculosis M1948 adOYA-...      52  0.001

 Mycobacterium tuberculosis H2463 [high GC Gram+] taxid 1448821
ref|NZ_KK337388.1| Mycobacterium tuberculosis H2463 adPcI-...      52  0.001

 Mycobacterium tuberculosis M13 [high GC Gram+] taxid 1448838
ref|NZ_KK338411.1| Mycobacterium tuberculosis M13 adOZj-su...      52  0.001

 Mycobacterium tuberculosis GM 1503 [high GC Gram+] taxid 537209
ref|NZ_KK338691.1| Mycobacterium tuberculosis GM 1503 adCA...      52  0.001

 Mycobacterium tuberculosis M2138 [high GC Gram+] taxid 1448763
ref|NZ_KK333410.1| Mycobacterium tuberculosis M2138 adPcW-...      52  0.001

 Mycobacterium tuberculosis M1429 [high GC Gram+] taxid 1448683
ref|NZ_KK328786.1| Mycobacterium tuberculosis M1429 adPdv-...      52  0.001

 Mycobacterium tuberculosis H3986 [high GC Gram+] taxid 1448824
ref|NZ_KK337617.1| Mycobacterium tuberculosis H3986 adPel-...      52  0.001

 Mycobacterium tuberculosis M1233 [high GC Gram+] taxid 1448639
ref|NZ_KK322014.1| Mycobacterium tuberculosis M1233 adPcT-...      52  0.001

 Mycobacterium tuberculosis M1560 [high GC Gram+] taxid 1448704
ref|NZ_KK329077.1| Mycobacterium tuberculosis M1560 adOZD-...      52  0.001

 Mycobacterium tuberculosis M1703 [high GC Gram+] taxid 1448709
ref|NZ_KK329473.1| Mycobacterium tuberculosis M1703 adOVL-...      52  0.001

 Mycobacterium tuberculosis M1928 [high GC Gram+] taxid 1448728
```

```
ref|NZ_KK330840.1| Mycobacterium tuberculosis M1928 adOYM-...      52  0.001

 Mycobacterium tuberculosis T17 [high GC Gram+] taxid 537210
ref|NZ_KK338511.1| Mycobacterium tuberculosis T17 adCAF-su...      52  0.001

 Mycobacterium tuberculosis M2134 [high GC Gram+] taxid 1448759
ref|NZ_KK333109.1| Mycobacterium tuberculosis M2134 adPgv-...      52  0.001

 Mycobacterium tuberculosis M2116 [high GC Gram+] taxid 1448753
ref|NZ_KK332674.1| Mycobacterium tuberculosis M2116 adOWK-...      52  0.001

 Mycobacterium tuberculosis M2129 [high GC Gram+] taxid 1448755
ref|NZ_KK332820.1| Mycobacterium tuberculosis M2129 adPfr-...      52  0.001

 Mycobacterium tuberculosis M1976 [high GC Gram+] taxid 1448738
ref|NZ_KK331624.1| Mycobacterium tuberculosis M1976 adPbH-...      52  0.001

 Mycobacterium tuberculosis M1011 [high GC Gram+] taxid 1448622
ref|NZ_KK321563.1| Mycobacterium tuberculosis M1011 adPfg-...      52  0.001

 Mycobacterium tuberculosis M2278 [high GC Gram+] taxid 1448783
ref|NZ_KK334702.1| Mycobacterium tuberculosis M2278 adPer-...      52  0.001

 Mycobacterium tuberculosis M2144 [high GC Gram+] taxid 1448800
ref|NZ_KK335942.1| Mycobacterium tuberculosis M2144 adOYY-...      52  0.001

 Mycobacterium tuberculosis M1979 [high GC Gram+] taxid 1448740
ref|NZ_KK331790.1| Mycobacterium tuberculosis M1979 adPcQ-...      52  0.001

 Mycobacterium tuberculosis M2343 [high GC Gram+] taxid 1448778
ref|NZ_KK334407.1| Mycobacterium tuberculosis M2343 adPbV-...      52  0.001

 Mycobacterium tuberculosis M1734 [high GC Gram+] taxid 1448713
ref|NZ_KK329789.1| Mycobacterium tuberculosis M1734 adPgC-...      52  0.001

 Mycobacterium tuberculosis M1025 [high GC Gram+] taxid 1448628
ref|NZ_KK321749.1| Mycobacterium tuberculosis M1025 adPae-...      52  0.001

 Mycobacterium tuberculosis H1578 [high GC Gram+] taxid 1448805
ref|NZ_KK336307.1| Mycobacterium tuberculosis H1578 adOVt-...      52  0.001

 Mycobacterium tuberculosis M2142 [high GC Gram+] taxid 1448788
ref|NZ_KK335072.1| Mycobacterium tuberculosis M2142 adOYv-...      52  0.001

 Mycobacterium tuberculosis M2416 [high GC Gram+] taxid 1448794
ref|NZ_KK335473.1| Mycobacterium tuberculosis M2416 adPbY-...      52  0.001

 Mycobacterium tuberculosis M2141 [high GC Gram+] taxid 1448776
ref|NZ_KK334317.1| Mycobacterium tuberculosis M2141 adPbj-...      52  0.001

 Mycobacterium tuberculosis M2128 [high GC Gram+] taxid 1448754
ref|NZ_KK332745.1| Mycobacterium tuberculosis M2128 adPcx-...      52  0.001

 Mycobacterium tuberculosis M2192 [high GC Gram+] taxid 1448774
ref|NZ_KK334168.1| Mycobacterium tuberculosis M2192 adOWF-...      52  0.001

 Mycobacterium tuberculosis M2003 [high GC Gram+] taxid 1448742
ref|NZ_KK331874.1| Mycobacterium tuberculosis M2003 adPaH-...      52  0.001

 Mycobacterium tuberculosis M1967 [high GC Gram+] taxid 1448736
ref|NZ_KK331459.1| Mycobacterium tuberculosis M1967 adPcd-...      52  0.001

 Mycobacterium tuberculosis M2021 [high GC Gram+] taxid 1448747
ref|NZ_KK332299.1| Mycobacterium tuberculosis M2021 adPfj-...      52  0.001

 Mycobacterium tuberculosis M2032 [high GC Gram+] taxid 1448748
ref|NZ_KK332370.1| Mycobacterium tuberculosis M2032 adPap-...      52  0.001

 Mycobacterium tuberculosis M2435 [high GC Gram+] taxid 1448792
ref|NZ_KK335403.1| Mycobacterium tuberculosis M2435 adPcD-...      52  0.001

 Mycobacterium tuberculosis H2102 [high GC Gram+] taxid 1448811
ref|NZ_KK336681.1| Mycobacterium tuberculosis H2102 adPcB-...      52  0.001

 Mycobacterium tuberculosis H2754 [high GC Gram+] taxid 1448822
ref|NZ_KK337458.1| Mycobacterium tuberculosis H2754 adOUL-...      52  0.001

 Mycobacterium tuberculosis M1236 [high GC Gram+] taxid 1448640
ref|NZ_KK322078.1| Mycobacterium tuberculosis M1236 adOUI-...      52  0.001

 Mycobacterium tuberculosis M2136 [high GC Gram+] taxid 1448760
ref|NZ_KK333181.1| Mycobacterium tuberculosis M2136 adOWv-...      52  0.001

 Mycobacterium tuberculosis H2438 [high GC Gram+] taxid 1448813
ref|NZ_KK336855.1| Mycobacterium tuberculosis H2438 adPeI-...      52  0.001

 Mycobacterium tuberculosis M1978 [high GC Gram+] taxid 1448739
ref|NZ_KK331708.1| Mycobacterium tuberculosis M1978 adPce-...      52  0.001

 Mycobacterium tuberculosis M1929 [high GC Gram+] taxid 1448729
ref|NZ_KK330931.1| Mycobacterium tuberculosis M1929 adPcC-...      52  0.001

 Mycobacterium tuberculosis H2264 [high GC Gram+] taxid 1448829
ref|NZ_KK337905.1| Mycobacterium tuberculosis H2264 adOVz-...      52  0.001

 Mycobacterium tuberculosis M2442 [high GC Gram+] taxid 1448790
ref|NZ_KK335231.1| Mycobacterium tuberculosis M2442 adPaq-...      52  0.001

 Mycobacterium tuberculosis MAL010134 [high GC Gram+] taxid 1354155
ref|NZ_KK338979.1| Mycobacterium tuberculosis MAL010134 ad...     52  0.001

 Mycobacterium tuberculosis M2139 [high GC Gram+] taxid 1448762
ref|NZ_KK333335.1| Mycobacterium tuberculosis M2139 adOVd-...      52  0.001

 Mycobacterium tuberculosis H1580 [high GC Gram+] taxid 1448806
ref|NZ_KK336380.1| Mycobacterium tuberculosis H1580 adPch-...      52  0.001

 Mycobacterium tuberculosis M1726 [high GC Gram+] taxid 1448711
```

```
ref|NZ_KK329631.1| Mycobacterium tuberculosis M1726 adOXx-...      52  0.001

 Mycobacterium tuberculosis M2206 [high GC Gram+] taxid 1448768
ref|NZ_KK333796.1| Mycobacterium tuberculosis M2206 adPeU-...      52  0.001

 Mycobacterium tuberculosis M1384 [high GC Gram+] taxid 1448667
ref|NZ_KK328533.1| Mycobacterium tuberculosis M1384 adPfe-...      52  0.001

 Mycobacterium tuberculosis M2211 [high GC Gram+] taxid 1448767
ref|NZ_KK333712.1| Mycobacterium tuberculosis M2211 adPaF-...      52  0.001

 Mycobacterium tuberculosis M2346 [high GC Gram+] taxid 1448836
ref|NZ_KK338246.1| Mycobacterium tuberculosis M2346 adPcw-...      52  0.001

 Mycobacterium tuberculosis M1034 [high GC Gram+] taxid 1448632
ref|NZ_KK321833.1| Mycobacterium tuberculosis M1034 adPag-...      52  0.001

 Mycobacterium tuberculosis H3033 [high GC Gram+] taxid 1448817
ref|NZ_KK337149.1| Mycobacterium tuberculosis H3033 adPaW-...      52  0.001

 Mycobacterium tuberculosis M2140 [high GC Gram+] taxid 1448764
ref|NZ_KK333483.1| Mycobacterium tuberculosis M2140 adOWz-...      52  0.001

 Mycobacterium tuberculosis M2218 [high GC Gram+] taxid 1448766
ref|NZ_KK333640.1| Mycobacterium tuberculosis M2218 adOZI-...      52  0.001

 Mycobacterium tuberculosis M2137 [high GC Gram+] taxid 1448761
ref|NZ_KK333260.1| Mycobacterium tuberculosis M2137 adPgE-...      52  0.001

 Mycobacterium tuberculosis M2352 [high GC Gram+] taxid 1448777
ref|NZ_JKJU01000006.1| Mycobacterium tuberculosis M2352 ad...      52  0.001

 Mycobacterium tuberculosis M2010 [high GC Gram+] taxid 1448745
ref|NZ_KK332146.1| Mycobacterium tuberculosis M2010 adPaJ-...      52  0.001

 Mycobacterium tuberculosis M2145 [high GC Gram+] taxid 1448799
ref|NZ_KK335870.1| Mycobacterium tuberculosis M2145 adPcj-...      52  0.001

 Mycobacterium tuberculosis M1570 [high GC Gram+] taxid 1448705
ref|NZ_KK329158.1| Mycobacterium tuberculosis M1570 adPei-...      52  0.001

 Mycobacterium tuberculosis M1030 [high GC Gram+] taxid 1448631
ref|NZ_JKPJ01000007.1| Mycobacterium tuberculosis M1030 ad...      52  0.001

 Mycobacterium tuberculosis M2203 [high GC Gram+] taxid 1448770
ref|NZ_KK333949.1| Mycobacterium tuberculosis M2203 adPfd-...      52  0.001

 Mycobacterium tuberculosis M2259 [high GC Gram+] taxid 1448786
ref|NZ_KK334929.1| Mycobacterium tuberculosis M2259 adPdO-...      52  0.001

 Mycobacterium tuberculosis H1589 [high GC Gram+] taxid 1448807
ref|NZ_KK336454.1| Mycobacterium tuberculosis H1589 adOXP-...      52  0.001

 Mycobacterium tuberculosis H3004 [high GC Gram+] taxid 1448815
ref|NZ_KK336999.1| Mycobacterium tuberculosis H3004 adPbA-...      52  0.001

 Mycobacterium tuberculosis H1249 [high GC Gram+] taxid 1448825
ref|NZ_KK337684.1| Mycobacterium tuberculosis H1249 adPey-...      52  0.001

 Mycobacterium tuberculosis M1816 [high GC Gram+] taxid 1448719
ref|NZ_KK330178.1| Mycobacterium tuberculosis M1816 adPdB-...      52  0.001

 Mycobacterium tuberculosis H3094 [high GC Gram+] taxid 1448823
ref|NZ_KK337533.1| Mycobacterium tuberculosis H3094 adOVs-...      52  0.001

 Mycobacterium tuberculosis M1959 [high GC Gram+] taxid 1448734
ref|NZ_KK331327.1| Mycobacterium tuberculosis M1959 adOVC-...      52  0.001

 Mycobacterium tuberculosis M2384 [high GC Gram+] taxid 1448802
ref|NZ_KK336096.1| Mycobacterium tuberculosis M2384 adOVQ-...      52  0.001

 Mycobacterium tuberculosis TKK_04_0120 [high GC Gram+] taxid 1448582
ref|NZ_KK321353.1| Mycobacterium tuberculosis TKK_04_0120 ...      52  0.001

 Mycobacterium tuberculosis H1996 [high GC Gram+] taxid 1448826
ref|NZ_KK337744.1| Mycobacterium tuberculosis H1996 adOYI-...      52  0.001

 Mycobacterium tuberculosis M2198 [high GC Gram+] taxid 1448772
ref|NZ_KK334090.1| Mycobacterium tuberculosis M2198 adPdQ-...      52  0.001

 Mycobacterium tuberculosis UT0046 [high GC Gram+] taxid 1408927
ref|NZ_JLPO01000004.1| Mycobacterium tuberculosis UT0046 a...      52  0.001

 Mycobacterium tuberculosis H2398 [high GC Gram+] taxid 1448830
ref|NZ_KK337978.1| Mycobacterium tuberculosis H2398 adOZz-...      52  0.001

 Mycobacterium tuberculosis T92 [high GC Gram+] taxid 515617
ref|NZ_DS985122.1| Mycobacterium tuberculosis T92 supercon...      52  0.001

 Mycobacterium tuberculosis H2375 [high GC Gram+] taxid 1448827
ref|NZ_KL406007.1| Mycobacterium tuberculosis H2375 adPeS-...      52  0.001
```

Taxonomy Report

root	1000 hits	566 orgs	
. Caudovirales	11 hits	11 orgs	[Viruses; dsDNA viruses, no RNA stage]
. . unclassified Caudovirales	2 hits	2 orgs	
. . . Bacillus phage phiNIT1	1 hits	1 orgs	
. . . Bacillus phage Grass	1 hits	1 orgs	
. . Siphoviridae	4 hits	4 orgs	
. . . unclassified Siphoviridae	2 hits	2 orgs	
. . . . Bacillus phage PM1	1 hits	1 orgs	
. . . . Staphylococcus phage StB20	1 hits	1 orgs	
. . . Bacillus phage SPP1	1 hits	1 orgs	[Lambdalikevirus; unclassified Lambda-like viruses]
. . . Bacillus phage SPbeta	1 hits	1 orgs	[Spbetalikevirus]
. . Myoviridae	5 hits	5 orgs	
. . . unclassified Myoviridae	4 hits	4 orgs	
. . . . Bacillus phage Bobb	1 hits	1 orgs	
. . . . Bacillus phage phiAGATE	1 hits	1 orgs	
. . . . Bacillus phage SP10	1 hits	1 orgs	
. . . . Bacillus phage CampHawk	1 hits	1 orgs	
. . . Bacillus phage SPO1	1 hits	1 orgs	[Spounavirinae; Spounalikevirus]
. cellular organisms	989 hits	555 orgs	
. . Bacteria	987 hits	553 orgs	
. . . Firmicutes	696 hits	303 orgs	
. . . . Bacillales	683 hits	290 orgs	[Bacilli]
. . . . . Bacillaceae	283 hits	149 orgs	
. . . . . . Bacillus	275 hits	141 orgs	
. . . . Bacillus sp. FJAT-14515	1 hits	1 orgs	
. . . . Bacillus sp. DW5-4	2 hits	1 orgs	
. . . . Bacillus pumilus	14 hits	7 orgs	
. . . . Bacillus pumilus SAFR-032	1 hits	1 orgs	
. . . . Bacillus pumilus ATCC 7061	1 hits	1 orgs	
. . . . Bacillus pumilus S-1	2 hits	1 orgs	
. . . . Bacillus pumilus INR7	2 hits	1 orgs	
. . . . Bacillus pumilus BA06	1 hits	1 orgs	
. . . . Bacillus pumilus CCMA-560	2 hits	1 orgs	
. . . . Bacillus sp. 171095_106	3 hits	1 orgs	
. . . . Bacillus xiamenensis	2 hits	1 orgs	
. . . . Bacillus megaterium	12 hits	6 orgs	
. . . . Bacillus megaterium WSH-002	1 hits	1 orgs	
. . . . Bacillus megaterium DSM 319	1 hits	1 orgs	
. . . . Bacillus megaterium NBRC 15308 = ATCC 14581	4 hits	1 orgs	
. . . . Bacillus megaterium NCT-2	4 hits	1 orgs	
. . . . Bacillus megaterium QM B1551	1 hits	1 orgs	
. . . . Bacillus sp. JGI 001006-L10	2 hits	1 orgs	
. . . . Bacillus sp. Aph1	4 hits	1 orgs	
. . . . Bacillus aryabhattai	3 hits	1 orgs	
. . . . Bacillus flexus	5 hits	2 orgs	
. . . . Bacillus flexus 27Col1.1E	3 hits	1 orgs	
. . . . Bacillus flexus T6186-2	2 hits	1 orgs	
. . . . Bacillus sp. RP1137	1 hits	1 orgs	
. . . . Bacillus altitudinis	3 hits	2 orgs	
. . . . Bacillus altitudinis 41KF2b	1 hits	1 orgs	
. . . . Bacillus sp. UNC125MFCrub1.1	1 hits	1 orgs	
. . . . Bacillus sp. 278922_107	2 hits	1 orgs	
. . . . Bacillus safensis	5 hits	3 orgs	

```
. . . . . . . . Bacillus safensis FO-36b ....................................     1 hits    1 orgs
. . . . . . . . Bacillus safensis VK ........................................     2 hits    1 orgs
. . . . . . . Bacillus firmus DS1 ............................................     1 hits    1 orgs [Bacillus firmus]
. . . . . . . Bacillus subtilis group ........................................   185 hits   91 orgs
. . . . . . . . Bacillus licheniformis .......................................    25 hits   11 orgs
. . . . . . . . . Bacillus licheniformis G-1 .................................     2 hits    1 orgs
. . . . . . . . . Bacillus licheniformis CG-B52 ..............................     3 hits    1 orgs
. . . . . . . . . Bacillus licheniformis 10-1-A ..............................     3 hits    1 orgs
. . . . . . . . . Bacillus licheniformis 5-2-D ...............................     3 hits    1 orgs
. . . . . . . . . Bacillus licheniformis F2-1 ................................     2 hits    1 orgs
. . . . . . . . . Bacillus licheniformis F1-1 ................................     2 hits    1 orgs
. . . . . . . . . Bacillus licheniformis WX-02 ...............................     1 hits    1 orgs
. . . . . . . . . Bacillus licheniformis DSM 13 = ATCC 14580 .................     2 hits    1 orgs
. . . . . . . . . Bacillus licheniformis 9945A ...............................     1 hits    1 orgs
. . . . . . . . . Bacillus licheniformis CGMCC 3963 ..........................     3 hits    1 orgs
. . . . . . . . Bacillus atrophaeus ..........................................    31 hits   15 orgs
. . . . . . . . . Bacillus atrophaeus UCMB-5137 ..............................     1 hits    1 orgs
. . . . . . . . . Bacillus atrophaeus C89 ....................................     3 hits    1 orgs
. . . . . . . . . Bacillus atrophaeus BACI051-N ..............................     3 hits    1 orgs
. . . . . . . . . Bacillus atrophaeus BACI051-E ..............................     2 hits    1 orgs
. . . . . . . . . Bacillus atrophaeus 1013-2 .................................     3 hits    1 orgs
. . . . . . . . . Bacillus atrophaeus subsp. globigii ........................    10 hits    5 orgs
. . . . . . . . . . Bacillus atrophaeus ATCC 49822 ...........................     4 hits    2 orgs
. . . . . . . . . . . Bacillus atrophaeus ATCC 49822-1 .......................     2 hits    1 orgs
. . . . . . . . . . . Bacillus atrophaeus ATCC 49822-2 .......................     2 hits    1 orgs
. . . . . . . . . . Bacillus atrophaeus ATCC 9372 ............................     5 hits    2 orgs
. . . . . . . . . . . Bacillus atrophaeus ATCC 9372-2 ........................     3 hits    1 orgs
. . . . . . . . . . . Bacillus atrophaeus ATCC 9372-1 ........................     2 hits    1 orgs
. . . . . . . . . Bacillus atrophaeus Detrick-2 ..............................     3 hits    1 orgs
. . . . . . . . . Bacillus atrophaeus Detrick-1 ..............................     2 hits    1 orgs
. . . . . . . . . Bacillus atrophaeus Detrick-3 ..............................     2 hits    1 orgs
. . . . . . . . . Bacillus atrophaeus 1013-1 .................................     1 hits    1 orgs
. . . . . . . . . Bacillus atrophaeus 1942 ...................................     1 hits    1 orgs
. . . . . . . . Bacillus subtilis ............................................    79 hits   36 orgs
. . . . . . . . . Bacillus subtilis subsp. niger .............................     1 hits    1 orgs
. . . . . . . . . Bacillus subtilis subsp. subtilis ..........................    28 hits   13 orgs
. . . . . . . . . . Bacillus subtilis subsp. subtilis str. SC-8 ..............     3 hits    1 orgs
. . . . . . . . . . Bacillus subtilis subsp. subtilis str. 168 ...............     4 hits    1 orgs
. . . . . . . . . . Bacillus subtilis subsp. subtilis str. JH642 .............     1 hits    1 orgs
. . . . . . . . . . Bacillus subtilis subsp. subtilis str. NCIB 3610 ..........     2 hits    2 orgs
. . . . . . . . . . . Bacillus subtilis subsp. subtilis 6051-HGW .............     1 hits    1 orgs
. . . . . . . . . . Bacillus subtilis subsp. subtilis str. SMY ...............     1 hits    1 orgs
. . . . . . . . . . Bacillus subtilis subsp. subtilis str. AUSI98 ............     4 hits    1 orgs
. . . . . . . . . . Bacillus subtilis subsp. subtilis str. MP9 ...............     3 hits    1 orgs
. . . . . . . . . . Bacillus subtilis subsp. subtilis str. MP11 ..............     3 hits    1 orgs
. . . . . . . . . . Bacillus subtilis subsp. subtilis str. BAB-1 .............     1 hits    1 orgs
. . . . . . . . . . Bacillus subtilis subsp. subtilis str. BSP1 ..............     1 hits    1 orgs
. . . . . . . . . . Bacillus subtilis subsp. subtilis str. RO-NN-1 ...........     1 hits    1 orgs
. . . . . . . . . Bacillus subtilis MB73/2 ...................................     2 hits    1 orgs
. . . . . . . . . Bacillus subtilis E1 .......................................     2 hits    1 orgs
. . . . . . . . . Bacillus subtilis QB928 ....................................     1 hits    1 orgs
. . . . . . . . . Bacillus subtilis PS216 ....................................     5 hits    1 orgs
. . . . . . . . . Bacillus subtilis Hal1 .....................................     3 hits    1 orgs
. . . . . . . . . Bacillus subtilis subsp. spizizenii ........................     5 hits    4 orgs
```

```
. . . . . . . . . . Bacillus subtilis subsp. spizizenii DV1-B-1 ..............     1 hits    1 orgs
. . . . . . . . . . Bacillus subtilis subsp. spizizenii ATCC 6633 ............     2 hits    1 orgs
. . . . . . . . . . Bacillus subtilis subsp. spizizenii str. W23 .............     1 hits    1 orgs
. . . . . . . . . . Bacillus subtilis subsp. spizizenii TU-B-10 ...............    1 hits    1 orgs
. . . . . . . . . Bacillus subtilis GB03 .....................................     1 hits    1 orgs
. . . . . . . . . Bacillus subtilis gtP20b ...................................     3 hits    1 orgs
. . . . . . . . . Bacillus subtilis subsp. inaquosorum KCTC 13429 ............     2 hits    1 orgs [Bacillus subtilis subsp. inaquosorum]
. . . . . . . . . Bacillus subtilis SPZ1 .....................................     4 hits    1 orgs
. . . . . . . . . Bacillus subtilis XF-1 .....................................     1 hits    1 orgs
. . . . . . . . . Bacillus subtilis S1-4 .....................................     3 hits    1 orgs
. . . . . . . . . Bacillus subtilis PTS-394 ..................................     1 hits    1 orgs
. . . . . . . . . Bacillus subtilis QH-1 .....................................     1 hits    1 orgs
. . . . . . . . . Bacillus subtilis PY79 .....................................     1 hits    1 orgs
. . . . . . . . . Bacillus subtilis BSn5 .....................................     1 hits    1 orgs
. . . . . . . . . Bacillus subtilis Miyagi-4 .................................     3 hits    1 orgs
. . . . . . . . . Bacillus subtilis subsp. natto BEST195 .....................     1 hits    1 orgs [Bacillus subtilis subsp. natto]
. . . . . . . . Bacillus sonorensis NBRC 101234 = KCTC 13918 .................     3 hits    1 orgs [Bacillus sonorensis]
. . . . . . . . Bacillus amyloliquefaciens ...................................    36 hits   24 orgs
. . . . . . . . . Bacillus amyloliquefaciens TA208 ...........................     1 hits    1 orgs
. . . . . . . . . Bacillus amyloliquefaciens XH7 .............................     1 hits    1 orgs
. . . . . . . . . Bacillus amyloliquefaciens subsp. amyloliquefaciens ........     2 hits    2 orgs
. . . . . . . . . . Bacillus amyloliquefaciens DSM 7 .........................     1 hits    1 orgs
. . . . . . . . . . Bacillus amyloliquefaciens subsp. amyloliquefaciens DC-12 .    1 hits    1 orgs
. . . . . . . . . Bacillus amyloliquefaciens LL3 .............................     1 hits    1 orgs
. . . . . . . . . Bacillus amyloliquefaciens CC178 ...........................     1 hits    1 orgs
. . . . . . . . . Bacillus amyloliquefaciens subsp. plantarum ................    14 hits   10 orgs
. . . . . . . . . . Bacillus amyloliquefaciens subsp. plantarum str. FZB42 ....     1 hits    1 orgs
. . . . . . . . . . Bacillus amyloliquefaciens subsp. plantarum UCMB5036 ......     1 hits    1 orgs
. . . . . . . . . . Bacillus amyloliquefaciens subsp. plantarum YAU B9601-Y2 ..     1 hits    1 orgs
. . . . . . . . . . Bacillus amyloliquefaciens subsp. plantarum NAU-B3 ........     1 hits    1 orgs
. . . . . . . . . . Bacillus amyloliquefaciens subsp. plantarum AS43.3 ........     1 hits    1 orgs
. . . . . . . . . . Bacillus amyloliquefaciens subsp. plantarum M27 ...........     1 hits    1 orgs
. . . . . . . . . . Bacillus amyloliquefaciens subsp. plantarum UCMB5113 ......     1 hits    1 orgs
. . . . . . . . . . Bacillus amyloliquefaciens subsp. plantarum UCMB5033 ......     1 hits    1 orgs
. . . . . . . . . . Bacillus amyloliquefaciens subsp. plantarum CAU B946 ......     1 hits    1 orgs
. . . . . . . . . Bacillus amyloliquefaciens HB-26 ...........................     2 hits    1 orgs
. . . . . . . . . Bacillus amyloliquefaciens Y2 ..............................     1 hits    1 orgs
. . . . . . . . . Bacillus amyloliquefaciens EGD-AQ14 ........................     2 hits    1 orgs
. . . . . . . . . Bacillus amyloliquefaciens UASWS BA1 .......................     1 hits    1 orgs
. . . . . . . . . Bacillus amyloliquefaciens IT-45 ...........................     1 hits    1 orgs
. . . . . . . . . Bacillus amyloliquefaciens LFB112 ..........................     1 hits    1 orgs
. . . . . . . . . Bacillus amyloliquefaciens EBL11 ...........................     2 hits    1 orgs
. . . . . . . . Bacillus vallismortis DV1-F-3 ................................     3 hits    1 orgs [Bacillus vallismortis]
. . . . . . . . Bacillus tequilensis KCTC 13622 ..............................     1 hits    1 orgs [Bacillus tequilensis]
. . . . . . . . Bacillus mojavensis ..........................................     7 hits    2 orgs [Bacillus mojavensis subgroup]
. . . . . . . . . Bacillus mojavensis RO-H-1 = KCTC 3706 .....................     4 hits    1 orgs
. . . . . . . . . Bacillus mojavensis RRC 101 ................................     3 hits    1 orgs
. . . . . . . Bacillus sp. CPSM8 .............................................     3 hits    1 orgs
. . . . . . . Bacillus sp. MSP5.4 ............................................     3 hits    1 orgs
. . . . . . . Bacillus sp. SB47 ..............................................     1 hits    1 orgs
. . . . . . . Bacillus sp. BT1B_CT2 ..........................................     1 hits    1 orgs
. . . . . . . Bacillus cereus ................................................     2 hits    2 orgs [Bacillus cereus group]
. . . . . . . . Bacillus cereus AH1272 .......................................     1 hits    1 orgs
. . . . . . . . Bacillus cereus AH1273 .......................................     1 hits    1 orgs
```

```
. . . . . . . Bacillus sp. NSP9.1 ............................................     1 hits    1 orgs
. . . . . . . Bacillus endophyticus 2102 .....................................     2 hits    1 orgs [Bacillus endophyticus]
. . . . . . . Bacillus siamensis KCTC 13613 ..................................     1 hits    1 orgs [Bacillus siamensis]
. . . . . . . Bacillus sp. EGD-AK10 ..........................................     1 hits    1 orgs
. . . . . . . Bacillus sp. MSP13 .............................................     2 hits    1 orgs
. . . . . . . Bacillus sp. 5B6 ...............................................     1 hits    1 orgs
. . . . . . . Bacillus sp. JS ................................................     1 hits    1 orgs
. . . . . . . Bacillus methylotrophicus SK19.001 .............................     3 hits    1 orgs [Bacillus methylotrophicus]
. . . . . . . Bacillus sp. UNC69MF ...........................................     1 hits    1 orgs
. . . . . . . Bacillus sp. 916 ...............................................     2 hits    1 orgs
. . . . . . . Bacillus panaciterrae DSM 19096 ................................     1 hits    1 orgs [Bacillus panaciterrae]
. . . . . . . Bacillus simplex BA2H3 .........................................     1 hits    1 orgs [Bacillus simplex]
. . . . . . . Bacillus sp. FJAT-13831 ........................................     2 hits    1 orgs
. . . . . . Halobacillus .....................................................     5 hits    5 orgs
. . . . . . . Halobacillus sp. BBL2006 .......................................     1 hits    1 orgs
. . . . . . . Halobacillus halophilus DSM 2266 ...............................     1 hits    1 orgs [Halobacillus halophilus]
. . . . . . . Halobacillus karajensis ........................................     1 hits    1 orgs
. . . . . . . Halobacillus dabanensis ........................................     1 hits    1 orgs
. . . . . . . Halobacillus trueperi ..........................................     1 hits    1 orgs
. . . . . . Marinococcus halotolerans DSM 16375 ..............................     1 hits    1 orgs [Marinococcus; Marinococcus halotolerans]
. . . . . . Oceanobacillus ...................................................     2 hits    2 orgs
. . . . . . . Oceanobacillus iheyensis HTE831 ................................     1 hits    1 orgs [Oceanobacillus iheyensis]
. . . . . . . Oceanobacillus kimchii X50 .....................................     1 hits    1 orgs [Oceanobacillus kimchii]
. . . . . Staphylococcus .....................................................   390 hits  136 orgs [Staphylococcaceae]
. . . . . . Staphylococcus aureus subsp. aureus CO-98 ........................     4 hits    1 orgs [Staphylococcus aureus; Staphylococcus aureus subsp. aureus]
. . . . . . Staphylococcus massiliensis CCUG 55927 ...........................     1 hits    1 orgs [Staphylococcus massiliensis]
. . . . . . Staphylococcus epidermidis .......................................   270 hits   86 orgs
. . . . . . . Staphylococcus epidermidis NIHLM021 ............................     5 hits    1 orgs
. . . . . . . Staphylococcus epidermidis NIHLM040 ............................     4 hits    1 orgs
. . . . . . . Staphylococcus epidermidis AP027 ...............................     3 hits    1 orgs
. . . . . . . Staphylococcus epidermidis VCU014 ..............................     4 hits    1 orgs
. . . . . . . Staphylococcus epidermidis VCU013 ..............................     4 hits    1 orgs
. . . . . . . Staphylococcus epidermidis AP035 ...............................     6 hits    1 orgs
. . . . . . . Staphylococcus epidermidis Scl22 ...............................     3 hits    1 orgs
. . . . . . . Staphylococcus epidermidis MC28 ................................     3 hits    1 orgs
. . . . . . . Staphylococcus epidermidis MC16 ................................     3 hits    1 orgs
. . . . . . . Staphylococcus epidermidis CIM37 ...............................     3 hits    1 orgs
. . . . . . . Staphylococcus epidermidis MC19 ................................     3 hits    1 orgs
. . . . . . . Staphylococcus epidermidis CIM40 ...............................     3 hits    1 orgs
. . . . . . . Staphylococcus epidermidis Scl19 ...............................     2 hits    1 orgs
. . . . . . . Staphylococcus epidermidis FRI909 ..............................     3 hits    1 orgs
. . . . . . . Staphylococcus epidermidis WI05 ................................     3 hits    1 orgs
. . . . . . . Staphylococcus epidermidis WI09 ................................     1 hits    1 orgs
. . . . . . . Staphylococcus epidermidis CIM28 ...............................     1 hits    1 orgs
. . . . . . . Staphylococcus epidermidis VCU144 ..............................     4 hits    1 orgs
. . . . . . . Staphylococcus epidermidis 12142587 ............................     5 hits    1 orgs
. . . . . . . Staphylococcus epidermidis VCU065 ..............................     4 hits    1 orgs
. . . . . . . Staphylococcus epidermidis VCU123 ..............................     4 hits    1 orgs
. . . . . . . Staphylococcus epidermidis NIHLM015 ............................     3 hits    1 orgs
. . . . . . . Staphylococcus epidermidis M23864:W2(grey) .....................     2 hits    1 orgs
. . . . . . . Staphylococcus epidermidis NIHLM037 ............................     4 hits    1 orgs
. . . . . . . Staphylococcus epidermidis VCU071 ..............................     3 hits    1 orgs
. . . . . . . Staphylococcus epidermidis NIHLM031 ............................     3 hits    1 orgs
. . . . . . . Staphylococcus epidermidis UC7032 ..............................     3 hits    1 orgs
```

. . . . Staphylococcus epidermidis VCU118 .... 3 hits 1 orgs
. . . . Staphylococcus epidermidis VCU129 .... 3 hits 1 orgs
. . . . Staphylococcus epidermidis VCU128 .... 3 hits 1 orgs
. . . . Staphylococcus epidermidis NIHLM049 .... 4 hits 1 orgs
. . . . Staphylococcus epidermidis VCU041 .... 4 hits 1 orgs
. . . . Staphylococcus epidermidis NIH05001 .... 3 hits 1 orgs
. . . . Staphylococcus epidermidis NIHLM061 .... 3 hits 1 orgs
. . . . Staphylococcus epidermidis NIHLM023 .... 3 hits 1 orgs
. . . . Staphylococcus epidermidis W23144 .... 2 hits 1 orgs
. . . . Staphylococcus epidermidis ATCC 12228 .... 1 hits 1 orgs
. . . . Staphylococcus epidermidis NIH06004 .... 3 hits 1 orgs
. . . . Staphylococcus epidermidis NIH08001 .... 3 hits 1 orgs
. . . . Staphylococcus epidermidis M0026 .... 3 hits 1 orgs
. . . . Staphylococcus epidermidis VCU045 .... 3 hits 1 orgs
. . . . Staphylococcus epidermidis NIH04003 .... 3 hits 1 orgs
. . . . Staphylococcus epidermidis VCU037 .... 3 hits 1 orgs
. . . . Staphylococcus epidermidis M0881 .... 3 hits 1 orgs
. . . . Staphylococcus epidermidis NIHLM001 .... 4 hits 1 orgs
. . . . Staphylococcus epidermidis RP62A .... 1 hits 1 orgs
. . . . Staphylococcus epidermidis NIHLM003 .... 3 hits 1 orgs
. . . . Staphylococcus epidermidis NIHLM053 .... 3 hits 1 orgs
. . . . Staphylococcus epidermidis NIHLM057 .... 3 hits 1 orgs
. . . . Staphylococcus epidermidis NIHLM070 .... 3 hits 1 orgs
. . . . Staphylococcus epidermidis VCU125 .... 3 hits 1 orgs
. . . . Staphylococcus epidermidis VCU127 .... 3 hits 1 orgs
. . . . Staphylococcus epidermidis IS-250 .... 3 hits 1 orgs
. . . . Staphylococcus epidermidis IS-K .... 3 hits 1 orgs
. . . . Staphylococcus epidermidis NIHLM008 .... 3 hits 1 orgs
. . . . Staphylococcus epidermidis NIHLM088 .... 3 hits 1 orgs
. . . . Staphylococcus epidermidis BVS058A4 .... 2 hits 1 orgs
. . . . Staphylococcus epidermidis 14.1.R1.SE .... 3 hits 1 orgs
. . . . Staphylococcus epidermidis NIHLM095 .... 3 hits 1 orgs
. . . . Staphylococcus epidermidis NIHLM087 .... 3 hits 1 orgs
. . . . Staphylococcus epidermidis AG42 .... 2 hits 1 orgs
. . . . Staphylococcus epidermidis Scl31 .... 3 hits 1 orgs
. . . . Staphylococcus epidermidis VCU126 .... 3 hits 1 orgs
. . . . Staphylococcus epidermidis VCU111 .... 3 hits 1 orgs
. . . . Staphylococcus epidermidis VCU117 .... 3 hits 1 orgs
. . . . Staphylococcus epidermidis NIH05003 .... 3 hits 1 orgs
. . . . Staphylococcus epidermidis NIHLM020 .... 3 hits 1 orgs
. . . . Staphylococcus epidermidis NIH051668 .... 3 hits 1 orgs
. . . . Staphylococcus epidermidis NIHLM039 .... 3 hits 1 orgs
. . . . Staphylococcus epidermidis VCU105 .... 3 hits 1 orgs
. . . . Staphylococcus epidermidis VCU120 .... 3 hits 1 orgs
. . . . Staphylococcus epidermidis NIHLM067 .... 3 hits 1 orgs
. . . . Staphylococcus epidermidis NIHLM018 .... 3 hits 1 orgs
. . . . Staphylococcus epidermidis NIH05005 .... 3 hits 1 orgs
. . . . Staphylococcus epidermidis VCU050 .... 3 hits 1 orgs
. . . . Staphylococcus epidermidis Scl25 .... 2 hits 1 orgs
. . . . Staphylococcus epidermidis VCU109 .... 3 hits 1 orgs
. . . . Staphylococcus epidermidis VCU081 .... 3 hits 1 orgs
. . . . Staphylococcus epidermidis VCU036 .... 3 hits 1 orgs
. . . . Staphylococcus epidermidis AU12-03 .... 3 hits 1 orgs
. . . . Staphylococcus epidermidis VCU028 .... 3 hits 1 orgs

```
. . . . . . . . Staphylococcus epidermidis NIH051475 ............................    3 hits    1 orgs
. . . . . . . . Staphylococcus epidermidis SK135 ................................    3 hits    1 orgs
. . . . . . . . Staphylococcus epidermidis BCM-HMP0060 ..........................    2 hits    1 orgs
. . . . . . . . Staphylococcus epidermidis E13A .................................    3 hits    1 orgs
. . . . . . . Staphylococcus haemolyticus .......................................   15 hits    4 orgs
. . . . . . . . Staphylococcus haemolyticus R1P1 ................................    2 hits    1 orgs
. . . . . . . . Staphylococcus haemolyticus DNF00585 ............................    2 hits    1 orgs
. . . . . . . . Staphylococcus haemolyticus JCSC1435 ............................    1 hits    1 orgs
. . . . . . . Staphylococcus capitis ............................................   12 hits    5 orgs
. . . . . . . . Staphylococcus capitis C87 ......................................    2 hits    1 orgs
. . . . . . . . Staphylococcus capitis VCU116 ...................................    3 hits    1 orgs
. . . . . . . . Staphylococcus capitis CR01 .....................................    1 hits    1 orgs
. . . . . . . . Staphylococcus capitis QN1 ......................................    3 hits    1 orgs
. . . . . . . Staphylococcus sp. EGD-HP3 ........................................    2 hits    1 orgs
. . . . . . . Staphylococcus warneri ............................................   15 hits    6 orgs
. . . . . . . . Staphylococcus warneri Lyso 1 2011 ..............................    3 hits    1 orgs
. . . . . . . . Staphylococcus warneri Lyso 2 2011 ..............................    3 hits    1 orgs
. . . . . . . . Staphylococcus warneri SG1 ......................................    1 hits    1 orgs
. . . . . . . . Staphylococcus warneri A487 .....................................    3 hits    1 orgs
. . . . . . . . Staphylococcus warneri L37603 ...................................    3 hits    1 orgs
. . . . . . . Staphylococcus pasteuri SP1 .......................................    1 hits    1 orgs [Staphylococcus pasteuri]
. . . . . . . Staphylococcus arlettae CVD059 ....................................    3 hits    1 orgs [Staphylococcus arlettae]
. . . . . . . Staphylococcus sp. URHA0057 .......................................    3 hits    1 orgs
. . . . . . . Staphylococcus xylosus ............................................    8 hits    3 orgs
. . . . . . . . Staphylococcus xylosus NJ .......................................    2 hits    1 orgs
. . . . . . . . Staphylococcus xylosus DMB3-Bh1 .................................    3 hits    1 orgs
. . . . . . . Staphylococcus sp. JGI 0001002-I23 ................................    3 hits    1 orgs
. . . . . . . Staphylococcus sp. MDS7B ..........................................    5 hits    1 orgs
. . . . . . . Staphylococcus sp. TE8 ............................................    2 hits    1 orgs
. . . . . . . Staphylococcus chromogenes MU 970 .................................    1 hits    1 orgs [Staphylococcus chromogenes]
. . . . . . . Staphylococcus lugdunensis ........................................   18 hits    8 orgs
. . . . . . . . Staphylococcus lugdunensis VCU148 ...............................    3 hits    1 orgs
. . . . . . . . Staphylococcus lugdunensis VCU139 ...............................    3 hits    1 orgs
. . . . . . . . Staphylococcus lugdunensis UCIM6116 .............................    3 hits    1 orgs
. . . . . . . . Staphylococcus lugdunensis ACS-027-V-Sch2 .......................    2 hits    1 orgs
. . . . . . . . Staphylococcus lugdunensis VCU150 ...............................    3 hits    1 orgs
. . . . . . . . Staphylococcus lugdunensis M23590 ...............................    2 hits    1 orgs
. . . . . . . . Staphylococcus lugdunensis N920143 ..............................    1 hits    1 orgs
. . . . . . . . Staphylococcus lugdunensis HKU09-01 .............................    1 hits    1 orgs
. . . . . . . Staphylococcus hominis ............................................    6 hits    3 orgs
. . . . . . . . Staphylococcus hominis subsp. hominis ...........................    4 hits    2 orgs
. . . . . . . . . Staphylococcus hominis subsp. hominis ZBW5 ....................    3 hits    1 orgs
. . . . . . . . . Staphylococcus hominis subsp. hominis C80 .....................    1 hits    1 orgs
. . . . . . . . Staphylococcus hominis VCU122 ...................................    2 hits    1 orgs
. . . . . . . Staphylococcus sp. M0480 ..........................................    3 hits    1 orgs
. . . . . . . Staphylococcus equorum UMC-CNS-924 ................................    2 hits    1 orgs [Staphylococcus equorum]
. . . . . . . Staphylococcus sp. OJ82 ...........................................    2 hits    1 orgs
. . . . . . . Staphylococcus simulans ...........................................    2 hits    2 orgs
. . . . . . . . Staphylococcus simulans ACS-120-V-Sch1 ..........................    1 hits    1 orgs
. . . . . . . . Staphylococcus simulans UMC-CNS-990 .............................    1 hits    1 orgs
. . . . . . . Staphylococcus sp. AL1 ............................................    3 hits    1 orgs
. . . . . . . Staphylococcus agnetis ............................................    1 hits    1 orgs
. . . . . . . Staphylococcus caprae M23864:W1 ...................................    2 hits    1 orgs [Staphylococcus caprae]
. . . . . . . Staphylococcus saprophyticus subsp. saprophyticus .................    4 hits    2 orgs [Staphylococcus saprophyticus]
```

. . . . Staphylococcus saprophyticus subsp. saprophyticus KACC 16562 .... 3 hits 1 orgs
. . . . Staphylococcus saprophyticus subsp. saprophyticus ATCC 15305 .... 1 hits 1 orgs
. . . . . . Staphylococcus sp. E463 .... 2 hits 1 orgs
. . . . . Thermoactinomycetaceae .... 3 hits 3 orgs
. . . . . . Laceyella sacchari 1-1 .... 1 hits 1 orgs [Laceyella; Laceyella sacchari]
. . . . . . Thermoactinomyces .... 2 hits 2 orgs
. . . . Thermoactinomyces sp. Gus2-1 .... 1 hits 1 orgs
. . . . Thermoactinomyces daqus .... 1 hits 1 orgs
. . . . . Paenibacillus polymyxa ATCC 12321 .... 4 hits 1 orgs [Paenibacillaceae; Paenibacillus; Paenibacillus polymyxa]
. . . . . Sporosarcina pasteurii NCIM 2477 .... 3 hits 1 orgs [Planococcaceae; Sporosarcina; Sporosarcina pasteurii]
. . . . Clostridiales .... 13 hits 13 orgs [Clostridia]
. . . . . Peptococcaceae .... 11 hits 11 orgs
. . . . . . Desulfotomaculum .... 3 hits 3 orgs
. . . . Desulfotomaculum alcoholivorax DSM 16058 .... 1 hits 1 orgs [Desulfotomaculum alcoholivorax]
. . . . Desulfotomaculum gibsoniae DSM 7213 .... 1 hits 1 orgs [Desulfotomaculum gibsoniae]
. . . . Desulfotomaculum ruminis DSM 2154 .... 1 hits 1 orgs [Desulfotomaculum ruminis]
. . . . . . Desulfosporosinus .... 2 hits 2 orgs
. . . . Desulfosporosinus orientis DSM 765 .... 1 hits 1 orgs [Desulfosporosinus orientis]
. . . . Desulfosporosinus youngiae DSM 17734 .... 1 hits 1 orgs [Desulfosporosinus youngiae]
. . . . . . Desulfitobacterium .... 6 hits 6 orgs
. . . . Desulfitobacterium hafniense .... 5 hits 5 orgs
. . . . Desulfitobacterium hafniense TCP-A .... 1 hits 1 orgs
. . . . Desulfitobacterium hafniense DP7 .... 1 hits 1 orgs
. . . . Desulfitobacterium hafniense PCP-1 .... 1 hits 1 orgs
. . . . Desulfitobacterium hafniense DCB-2 .... 1 hits 1 orgs
. . . . Desulfitobacterium hafniense Y51 .... 1 hits 1 orgs
. . . . Desulfitobacterium sp. PCE1 .... 1 hits 1 orgs
. . . . . Intestinibacter bartlettii .... 2 hits 2 orgs [Peptostreptococcaceae; Intestinibacter]
. . . . . . Intestinibacter bartlettii DORA_8_9 .... 1 hits 1 orgs
. . . . . . Intestinibacter bartlettii DSM 16795 .... 1 hits 1 orgs
. . . Actinobacteria .... 223 hits 185 orgs [Actinobacteria]
. . . . Actinomycetales .... 222 hits 184 orgs [Actinobacteridae]
. . . . . Streptomycetaceae .... 113 hits 78 orgs [Streptomycineae]
. . . . . . Streptomyces .... 112 hits 77 orgs
. . . . Streptomyces roseochromogenus subsp. oscitans DS 12.976 .... 1 hits 1 orgs [Streptomyces roseochromogenus; Streptomyces roseochromogenus subsp. oscitans]
. . . . Streptomyces aureocirculatus .... 4 hits 1 orgs
. . . . Streptomyces sp. PRh5 .... 1 hits 1 orgs
. . . . Streptomyces sp. NRRL S-920 .... 1 hits 1 orgs
. . . . Streptomyces sp. NRRL S-146 .... 1 hits 1 orgs
. . . . Streptomyces sp. NRRL WC-3773 .... 2 hits 1 orgs
. . . . Streptomyces sp. AW19M42 .... 1 hits 1 orgs
. . . . Streptomyces sp. NRRL F-6131 .... 1 hits 1 orgs
. . . . Streptomyces viridochromogenes .... 2 hits 2 orgs
. . . . Streptomyces viridochromogenes Tue57 .... 1 hits 1 orgs
. . . . Streptomyces viridochromogenes DSM 40736 .... 1 hits 1 orgs
. . . . Streptomyces sp. CNS654 .... 1 hits 1 orgs
. . . . Streptomyces purpeofuscus .... 1 hits 1 orgs
. . . . Streptomyces sp. NRRL B-5680 .... 1 hits 1 orgs
. . . . Streptomyces sp. e14 .... 1 hits 1 orgs
. . . . Streptomyces sp. Amel2xE9 .... 1 hits 1 orgs
. . . . Streptomyces aureofaciens .... 2 hits 1 orgs
. . . . Streptomyces avellaneus .... 1 hits 1 orgs
. . . . Streptomyces sp. NRRL S-244 .... 1 hits 1 orgs

```
. . . . . . . Streptomyces sp. NRRL F-5702 .... 1 hits 1 orgs
. . . . . . . Streptomyces purpeochromogenes .... 2 hits 1 orgs
. . . . . . . Streptomyces griseus group .... 17 hits 9 orgs
. . . . . . . . Streptomyces puniceus subgroup .... 7 hits 3 orgs
. . . . . . . . . Streptomyces californicus .... 4 hits 1 orgs
. . . . . . . . . Streptomyces puniceus .... 2 hits 1 orgs
. . . . . . . . . Streptomyces floridae .... 1 hits 1 orgs
. . . . . . . . Streptomyces griseus .... 8 hits 4 orgs [Streptomyces griseus subgroup]
. . . . . . . . . Streptomyces griseus subsp. rhodochrous .... 4 hits 1 orgs
. . . . . . . . . Streptomyces griseus subsp. griseus .... 3 hits 2 orgs
. . . . . . . . . . Streptomyces griseus subsp. griseus NBRC 13350 .... 1 hits 1 orgs
. . . . . . . . . Streptomyces griseus XylebKG-1 .... 1 hits 1 orgs
. . . . . . . . Streptomyces alboviridis .... 1 hits 1 orgs [Streptomyces microflavus subgroup]
. . . . . . . . Streptomyces cyaneofuscatus .... 1 hits 1 orgs
. . . . . . . Streptomyces sp. NRRL F-3273 .... 1 hits 1 orgs
. . . . . . . Streptomyces brasiliensis .... 1 hits 1 orgs
. . . . . . . Streptomyces sp. NRRL F-3218 .... 1 hits 1 orgs
. . . . . . . Streptomyces sp. NRRL WC-3540 .... 1 hits 1 orgs
. . . . . . . Streptomyces sp. SolWspMP-sol2th .... 1 hits 1 orgs
. . . . . . . Streptomyces sp. NRRL F-2202 .... 1 hits 1 orgs
. . . . . . . Streptomyces sp. NRRL F-5681 .... 1 hits 1 orgs
. . . . . . . Streptomyces sp. ScaeMP-e10 .... 1 hits 1 orgs
. . . . . . . Streptomyces sp. NRRL B-1381 .... 1 hits 1 orgs
. . . . . . . Streptomyces sp. Wigar10 .... 1 hits 1 orgs
. . . . . . . Streptomyces fulvissimus DSM 40593 .... 1 hits 1 orgs [Streptomyces fulvissimus]
. . . . . . . Streptomyces sp. NRRL S-623 .... 1 hits 1 orgs
. . . . . . . Streptomyces sp. SA3_actF .... 1 hits 1 orgs
. . . . . . . Streptomyces sp. W007 .... 1 hits 1 orgs
. . . . . . . Streptomyces flavochromogenes .... 1 hits 1 orgs
. . . . . . . Streptomyces sp. SA3_actG .... 1 hits 1 orgs
. . . . . . . Streptomyces purpureus KA281 .... 1 hits 1 orgs [Streptomyces purpureus]
. . . . . . . Streptomyces sp. NRRL F-5135 .... 1 hits 1 orgs
. . . . . . . Streptomyces sp. CcalMP-8W .... 1 hits 1 orgs
. . . . . . . Streptomyces sp. JS01 .... 1 hits 1 orgs
. . . . . . . Streptomyces sp. Tu6071 .... 1 hits 1 orgs
. . . . . . . Streptomyces sp. PCS3-D2 .... 1 hits 1 orgs
. . . . . . . Streptomyces sp. SPB78 .... 1 hits 1 orgs
. . . . . . . Streptomyces exfoliatus .... 3 hits 2 orgs
. . . . . . . . Streptomyces exfoliatus DSM 41693 .... 1 hits 1 orgs
. . . . . . . Streptomyces thermolilacinus SPC6 .... 1 hits 1 orgs [Streptomyces thermolilacinus]
. . . . . . . Streptomyces somaliensis DSM 40738 .... 1 hits 1 orgs [Streptomyces somaliensis]
. . . . . . . Streptomyces rimosus .... 21 hits 3 orgs
. . . . . . . . Streptomyces rimosus subsp. rimosus .... 20 hits 2 orgs
. . . . . . . . . Streptomyces rimosus subsp. rimosus ATCC 10970 .... 1 hits 1 orgs
. . . . . . . Streptomyces sp. NRRL B-11253 .... 1 hits 1 orgs
. . . . . . . Streptomyces peucetius .... 1 hits 1 orgs
. . . . . . . Streptomyces sp. NRRL WC-3719 .... 1 hits 1 orgs
. . . . . . . Streptomyces globisporus subsp. globisporus .... 1 hits 1 orgs [Streptomyces globisporus]
. . . . . . . Streptomyces sp. NRRL WC-3702 .... 1 hits 1 orgs
. . . . . . . Streptomyces capuensis .... 2 hits 1 orgs
. . . . . . . Streptomyces sp. NRRL WC-3703 .... 1 hits 1 orgs
. . . . . . . Streptomyces lavendulae subsp. lavendulae .... 2 hits 1 orgs [Streptomyces lavendulae]
. . . . . . . Streptomyces monomycini .... 1 hits 1 orgs
. . . . . . . Streptomyces wedmorensis .... 1 hits 1 orgs
```

```
. . . . . . . Streptomyces sp. SS ............................................     1 hits    1 orgs
. . . . . . . Streptomyces sp. NRRL F-5727 ...................................     1 hits    1 orgs
. . . . . . . Streptomyces sclerotialus ......................................     1 hits    1 orgs
. . . . . . . Streptomyces sp. CNS615 ........................................     1 hits    1 orgs
. . . . . . . Streptomyces griseoluteus ......................................     1 hits    1 orgs
. . . . . . . Streptomyces sp. CNR698 ........................................     1 hits    1 orgs
. . . . . . . Streptomyces sp. CNT302 ........................................     1 hits    1 orgs
. . . . . . . Streptomyces sp. NRRL WC-3725 ..................................     1 hits    1 orgs
. . . . . . Streptacidiphilus oryzae TH49 ....................................     1 hits    1 orgs [Streptacidiphilus; Streptacidiphilus oryzae]
. . . . . Pseudonocardiaceae .................................................     7 hits    6 orgs [Pseudonocardineae]
. . . . . . Prauserella ......................................................     2 hits    2 orgs
. . . . . . . Prauserella sp. Am3 ............................................     1 hits    1 orgs
. . . . . . . Prauserella rugosa .............................................     1 hits    1 orgs
. . . . . . Lentzea albidocapillata ..........................................     1 hits    1 orgs [Lentzea]
. . . . . . Saccharopolyspora erythraea ......................................     3 hits    2 orgs [Saccharopolyspora]
. . . . . . . Saccharopolyspora erythraea D ..................................     1 hits    1 orgs
. . . . . . . Saccharopolyspora erythraea NRRL 2338 ..........................     2 hits    1 orgs
. . . . . . Lechevalieria aerocolonigenes ....................................     1 hits    1 orgs [Lechevalieria]
. . . . . Streptosporangineae ................................................     4 hits    4 orgs
. . . . . . Nocardiopsis .....................................................     2 hits    2 orgs [Nocardiopsaceae]
. . . . . . . Nocardiopsis xinjiangensis YIM 90004 ...........................     1 hits    1 orgs [Nocardiopsis xinjiangensis]
. . . . . . . Nocardiopsis salina YIM 90010 ..................................     1 hits    1 orgs [Nocardiopsis salina]
. . . . . . Actinomadura oligospora ATCC 43269 ...............................     1 hits    1 orgs [Thermomonosporaceae; Actinomadura; Actinomadura oligospora]
. . . . . . Streptosporangium roseum .........................................     1 hits    1 orgs [Streptosporangiaceae; Streptosporangium]
. . . . . Micromonosporaceae .................................................     2 hits    2 orgs [Micromonosporineae]
. . . . . . Dactylosporangium aurantiacum ....................................     1 hits    1 orgs [Dactylosporangium]
. . . . . . Actinoplanes friuliensis DSM 7358 ................................     1 hits    1 orgs [Actinoplanes; Actinoplanes friuliensis]
. . . . . Corynebacterineae ..................................................    96 hits   94 orgs
. . . . . . Rhodococcus opacus B4 ............................................     1 hits    1 orgs [Nocardiaceae; Rhodococcus; Rhodococcus opacus]
. . . . . . Mycobacterium tuberculosis .......................................    95 hits   93 orgs [Mycobacteriaceae; Mycobacterium; Mycobacterium tuberculosis
complex]
. . . . . . . Mycobacterium tuberculosis M1906 ...............................     1 hits    1 orgs
. . . . . . . Mycobacterium tuberculosis H2760 ...............................     1 hits    1 orgs
. . . . . . . Mycobacterium tuberculosis H3005 ...............................     1 hits    1 orgs
. . . . . . . Mycobacterium tuberculosis H3361 ...............................     1 hits    1 orgs
. . . . . . . Mycobacterium tuberculosis NA-A0009 ............................     1 hits    1 orgs
. . . . . . . Mycobacterium tuberculosis NA-A0008 ............................     1 hits    1 orgs
. . . . . . . Mycobacterium tuberculosis M1893 ...............................     1 hits    1 orgs
. . . . . . . Mycobacterium tuberculosis M992 ................................     1 hits    1 orgs
. . . . . . . Mycobacterium tuberculosis 43-16836 ............................     1 hits    1 orgs
. . . . . . . Mycobacterium tuberculosis M1961 ...............................     1 hits    1 orgs
. . . . . . . Mycobacterium tuberculosis PR05 ................................     1 hits    1 orgs
. . . . . . . Mycobacterium tuberculosis M1029 ...............................     1 hits    1 orgs
. . . . . . . Mycobacterium tuberculosis H3367 ...............................     1 hits    1 orgs
. . . . . . . Mycobacterium tuberculosis TKK_04_0085 .........................     1 hits    1 orgs
. . . . . . . Mycobacterium tuberculosis M1221 ...............................     1 hits    1 orgs
. . . . . . . Mycobacterium tuberculosis M1948 ...............................     1 hits    1 orgs
. . . . . . . Mycobacterium tuberculosis H2463 ...............................     1 hits    1 orgs
. . . . . . . Mycobacterium tuberculosis M13 .................................     1 hits    1 orgs
. . . . . . . Mycobacterium tuberculosis GM 1503 .............................     1 hits    1 orgs
. . . . . . . Mycobacterium tuberculosis M2138 ...............................     1 hits    1 orgs
. . . . . . . Mycobacterium tuberculosis M1429 ...............................     1 hits    1 orgs
. . . . . . . Mycobacterium tuberculosis H3986 ...............................     1 hits    1 orgs
. . . . . . . Mycobacterium tuberculosis M1233 ...............................     1 hits    1 orgs
```

```
. . . . . . . Mycobacterium tuberculosis M1560 ................................     1 hits    1 orgs
. . . . . . . Mycobacterium tuberculosis M1703 ................................     1 hits    1 orgs
. . . . . . . Mycobacterium tuberculosis M1928 ................................     1 hits    1 orgs
. . . . . . . Mycobacterium tuberculosis T17 ..................................     1 hits    1 orgs
. . . . . . . Mycobacterium tuberculosis M2134 ................................     1 hits    1 orgs
. . . . . . . Mycobacterium tuberculosis M2116 ................................     1 hits    1 orgs
. . . . . . . Mycobacterium tuberculosis M2129 ................................     1 hits    1 orgs
. . . . . . . Mycobacterium tuberculosis M1976 ................................     1 hits    1 orgs
. . . . . . . Mycobacterium tuberculosis M1011 ................................     1 hits    1 orgs
. . . . . . . Mycobacterium tuberculosis M2278 ................................     1 hits    1 orgs
. . . . . . . Mycobacterium tuberculosis M2144 ................................     1 hits    1 orgs
. . . . . . . Mycobacterium tuberculosis M1979 ................................     1 hits    1 orgs
. . . . . . . Mycobacterium tuberculosis M2343 ................................     1 hits    1 orgs
. . . . . . . Mycobacterium tuberculosis M1734 ................................     1 hits    1 orgs
. . . . . . . Mycobacterium tuberculosis M1025 ................................     1 hits    1 orgs
. . . . . . . Mycobacterium tuberculosis H1578 ................................     1 hits    1 orgs
. . . . . . . Mycobacterium tuberculosis M2142 ................................     1 hits    1 orgs
. . . . . . . Mycobacterium tuberculosis M2416 ................................     1 hits    1 orgs
. . . . . . . Mycobacterium tuberculosis M2141 ................................     1 hits    1 orgs
. . . . . . . Mycobacterium tuberculosis M2128 ................................     1 hits    1 orgs
. . . . . . . Mycobacterium tuberculosis M2192 ................................     1 hits    1 orgs
. . . . . . . Mycobacterium tuberculosis M2003 ................................     1 hits    1 orgs
. . . . . . . Mycobacterium tuberculosis M1967 ................................     1 hits    1 orgs
. . . . . . . Mycobacterium tuberculosis M2021 ................................     1 hits    1 orgs
. . . . . . . Mycobacterium tuberculosis M2032 ................................     1 hits    1 orgs
. . . . . . . Mycobacterium tuberculosis M2435 ................................     1 hits    1 orgs
. . . . . . . Mycobacterium tuberculosis H2102 ................................     1 hits    1 orgs
. . . . . . . Mycobacterium tuberculosis H2754 ................................     1 hits    1 orgs
. . . . . . . Mycobacterium tuberculosis M1236 ................................     1 hits    1 orgs
. . . . . . . Mycobacterium tuberculosis M2136 ................................     1 hits    1 orgs
. . . . . . . Mycobacterium tuberculosis H2438 ................................     1 hits    1 orgs
. . . . . . . Mycobacterium tuberculosis M1978 ................................     1 hits    1 orgs
. . . . . . . Mycobacterium tuberculosis M1929 ................................     1 hits    1 orgs
. . . . . . . Mycobacterium tuberculosis H2264 ................................     1 hits    1 orgs
. . . . . . . Mycobacterium tuberculosis M2442 ................................     1 hits    1 orgs
. . . . . . . Mycobacterium tuberculosis MAL010134 ............................     1 hits    1 orgs
. . . . . . . Mycobacterium tuberculosis M2139 ................................     1 hits    1 orgs
. . . . . . . Mycobacterium tuberculosis H1580 ................................     1 hits    1 orgs
. . . . . . . Mycobacterium tuberculosis M1726 ................................     1 hits    1 orgs
. . . . . . . Mycobacterium tuberculosis M2206 ................................     1 hits    1 orgs
. . . . . . . Mycobacterium tuberculosis M1384 ................................     1 hits    1 orgs
. . . . . . . Mycobacterium tuberculosis M2211 ................................     1 hits    1 orgs
. . . . . . . Mycobacterium tuberculosis M2346 ................................     1 hits    1 orgs
. . . . . . . Mycobacterium tuberculosis M1034 ................................     1 hits    1 orgs
. . . . . . . Mycobacterium tuberculosis H3033 ................................     1 hits    1 orgs
. . . . . . . Mycobacterium tuberculosis M2140 ................................     1 hits    1 orgs
. . . . . . . Mycobacterium tuberculosis M2218 ................................     1 hits    1 orgs
. . . . . . . Mycobacterium tuberculosis M2137 ................................     1 hits    1 orgs
. . . . . . . Mycobacterium tuberculosis M2352 ................................     1 hits    1 orgs
. . . . . . . Mycobacterium tuberculosis M2010 ................................     1 hits    1 orgs
. . . . . . . Mycobacterium tuberculosis M2145 ................................     1 hits    1 orgs
. . . . . . . Mycobacterium tuberculosis M1570 ................................     1 hits    1 orgs
. . . . . . . Mycobacterium tuberculosis M1030 ................................     1 hits    1 orgs
. . . . . . . Mycobacterium tuberculosis M2203 ................................     1 hits    1 orgs
```

```
. . . . . . . Mycobacterium tuberculosis M2259 ...............................     1 hits    1 orgs
. . . . . . . Mycobacterium tuberculosis H1589 ...............................     1 hits    1 orgs
. . . . . . . Mycobacterium tuberculosis H3004 ...............................     1 hits    1 orgs
. . . . . . . Mycobacterium tuberculosis H1249 ...............................     1 hits    1 orgs
. . . . . . . Mycobacterium tuberculosis M1816 ...............................     1 hits    1 orgs
. . . . . . . Mycobacterium tuberculosis H3094 ...............................     1 hits    1 orgs
. . . . . . . Mycobacterium tuberculosis M1959 ...............................     1 hits    1 orgs
. . . . . . . Mycobacterium tuberculosis M2384 ...............................     1 hits    1 orgs
. . . . . . . Mycobacterium tuberculosis TKK_04_0120 .........................     1 hits    1 orgs
. . . . . . . Mycobacterium tuberculosis H1996 ...............................     1 hits    1 orgs
. . . . . . . Mycobacterium tuberculosis M2198 ...............................     1 hits    1 orgs
. . . . . . . Mycobacterium tuberculosis UT0046 ..............................     1 hits    1 orgs
. . . . . . . Mycobacterium tuberculosis H2398 ...............................     1 hits    1 orgs
. . . . . . . Mycobacterium tuberculosis T92 .................................     1 hits    1 orgs
. . . . . . . Mycobacterium tuberculosis H2375 ...............................     1 hits    1 orgs
. . . . marine actinobacterium MedAcidi-G1 ...................................     1 hits    1 orgs [unclassified Actinobacteria; unclassified Actinobacteria
(miscellaneous)]
. . . Proteobacteria .........................................................    65 hits   62 orgs
. . . . Alphaproteobacteria ..................................................    32 hits   30 orgs
. . . . . Rhodobacteraceae ...................................................     8 hits    8 orgs [Rhodobacterales]
. . . . . . Thalassobacter arenae DSM 19593 ..................................     1 hits    1 orgs [Thalassobacter; Thalassobacter arenae]
. . . . . . Dinoroseobacter shibae DFL 12 = DSM 16493 ........................     1 hits    1 orgs [Dinoroseobacter; Dinoroseobacter shibae]
. . . . . . Sulfitobacter ....................................................     6 hits    6 orgs
. . . . . . . Sulfitobacter sp. 20_GPM-1509m .................................     1 hits    1 orgs
. . . . . . . Sulfitobacter sp. CB2047 .......................................     1 hits    1 orgs
. . . . . . . Sulfitobacter sp. EE-36 ........................................     1 hits    1 orgs
. . . . . . . Sulfitobacter pontiacus 3SOLIMAR09 .............................     1 hits    1 orgs [Sulfitobacter pontiacus]
. . . . . . . Sulfitobacter sp. NAS-14.1 .....................................     1 hits    1 orgs
. . . . . . . Sulfitobacter sp. NB-68 ........................................     1 hits    1 orgs
. . . . . Rhizobiales ........................................................    24 hits   22 orgs
. . . . . . Bradyrhizobium ...................................................     2 hits    2 orgs [Bradyrhizobiaceae]
. . . . . . . Bradyrhizobium sp. CCBAU 43298 .................................     1 hits    1 orgs
. . . . . . . Bradyrhizobium elkanii WSM2783 .................................     1 hits    1 orgs [Bradyrhizobium elkanii]
. . . . . . Rhizobiaceae .....................................................    10 hits    8 orgs
. . . . . . . Rhizobium/Agrobacterium group ..................................     9 hits    7 orgs
. . . . . . . . Agrobacterium ................................................     4 hits    2 orgs
. . . . . . . . . Agrobacterium rhizogenes ...................................     2 hits    1 orgs
. . . . . . . . . Agrobacterium radiobacter K84 ..............................     2 hits    1 orgs [Agrobacterium tumefaciens complex; Agrobacterium tumefaciens]
. . . . . . . . Rhizobium ....................................................     5 hits    5 orgs
. . . . . . . . . Rhizobium tropici CIAT 899 .................................     1 hits    1 orgs [Rhizobium tropici]
. . . . . . . . . Rhizobium sp. JGI 001013-F22 ...............................     1 hits    1 orgs
. . . . . . . . . Rhizobium sp. YR060 ........................................     1 hits    1 orgs
. . . . . . . . . Rhizobium sp. OK494 ........................................     1 hits    1 orgs
. . . . . . . . . Rhizobium sp. PDO1-076 .....................................     1 hits    1 orgs
. . . . . . . Sinorhizobium fredii HH103 .....................................     1 hits    1 orgs [Sinorhizobium/Ensifer group; Sinorhizobium; Sinorhizobium
fredii group; Sinorhizobium fredii]
. . . . . . Mesorhizobium ....................................................     9 hits    9 orgs [Phyllobacteriaceae]
. . . . . . . Mesorhizobium sp. WSM3224 ......................................     1 hits    1 orgs
. . . . . . . Mesorhizobium sp. LNJC384A00 ...................................     1 hits    1 orgs
. . . . . . . Mesorhizobium australicum WSM2073 ..............................     1 hits    1 orgs [Mesorhizobium australicum]
. . . . . . . Mesorhizobium sp. LSJC255A00 ...................................     1 hits    1 orgs
. . . . . . . Mesorhizobium sp. LSHC414A00 ...................................     1 hits    1 orgs
. . . . . . . Mesorhizobium loti .............................................     2 hits    2 orgs
. . . . . . . . Mesorhizobium loti USDA 3471 .................................     1 hits    1 orgs
```

. . . . Mesorhizobium loti MAFF303099 ... 1 hits 1 orgs
. . . . Mesorhizobium sp. LNJC386A00 ... 1 hits 1 orgs
. . . . Mesorhizobium sp. LSJC285A00 ... 1 hits 1 orgs
. . . . . . Methylocystis ... 2 hits 2 orgs [Methylocystaceae]
. . . . Methylocystis sp. ATCC 49242 ... 1 hits 1 orgs
. . . . Methylocystis parvus OBBP ... 1 hits 1 orgs [Methylocystis parvus]
. . . . . . Ochrobactrum anthropi 60a ... 1 hits 1 orgs [Brucellaceae; Ochrobactrum; Ochrobactrum anthropi]
. . . . Deltaproteobacteria ... 8 hits 8 orgs [delta/epsilon subdivisions]
. . . . . Desulfobacterales ... 5 hits 5 orgs
. . . . . . Desulfobulbus sp. Tol-SR ... 1 hits 1 orgs [Desulfobulbaceae; Desulfobulbus]
. . . . . . Desulfobacteraceae ... 4 hits 4 orgs
. . . . Desulfobacterium autotrophicum HRM2 ... 1 hits 1 orgs [Desulfobacterium; Desulfobacterium autotrophicum]
. . . . Desulfatirhabdium butyrativorans DSM 18734 ... 1 hits 1 orgs [Desulfatirhabdium; Desulfatirhabdium butyrativorans]
. . . . Desulfospira joergensenii DSM 10085 ... 1 hits 1 orgs [Desulfospira; Desulfospira joergensenii]
. . . . Desulfatiglans anilini DSM 4660 ... 1 hits 1 orgs [Desulfatiglans; Desulfatiglans anilini]
. . . . . Syntrophobacter fumaroxidans MPOB ... 1 hits 1 orgs [Syntrophobacterales; Syntrophobacteraceae; Syntrophobacter; Syntrophobacter fumaroxidans]
. . . . . Pelobacter seleniigenes DSM 18267 ... 1 hits 1 orgs [Desulfuromonadales; Pelobacteraceae; Pelobacter; Pelobacter seleniigenes]
. . . . . Candidatus Entotheonella sp. TSY1 ... 1 hits 1 orgs [unclassified Deltaproteobacteria; Candidatus Entotheonella]
. . . . Betaproteobacteria ... 13 hits 13 orgs
. . . . . Rhodocyclaceae ... 7 hits 7 orgs [Rhodocyclales]
. . . . . . Rhodocyclaceae bacterium RZ94 ... 1 hits 1 orgs [unclassified Rhodocyclaceae]
. . . . . . Methyloversatilis ... 4 hits 4 orgs
. . . . Methyloversatilis sp. FAM1 ... 1 hits 1 orgs
. . . . Methyloversatilis universalis ... 2 hits 2 orgs
. . . . Methyloversatilis sp. RZ18-153 ... 1 hits 1 orgs
. . . . Methyloversatilis universalis EHg5 ... 1 hits 1 orgs
. . . . Methyloversatilis sp. NVD ... 1 hits 1 orgs
. . . . . . Thauera sp. MZ1T ... 1 hits 1 orgs [Thauera]
. . . . . . Uliginosibacterium gangwonense DSM 18521 ... 1 hits 1 orgs [Uliginosibacterium; Uliginosibacterium gangwonense]
. . . . . Methylotenera sp. 1P/1 ... 1 hits 1 orgs [Methylophilales; Methylophilaceae; Methylotenera]
. . . . . Burkholderiales ... 5 hits 5 orgs
. . . . . . Comamonadaceae ... 2 hits 2 orgs
. . . . Comamonas badia DSM 17552 ... 1 hits 1 orgs [Comamonas; Comamonas badia]
. . . . Curvibacter gracilis ATCC BAA-807 ... 1 hits 1 orgs [Curvibacter; Curvibacter gracilis]
. . . . . . Burkholderia phenoliruptrix AC1100 ... 1 hits 1 orgs [Burkholderiaceae; Burkholderia; Burkholderia phenoliruptrix]
. . . . . . Methylibium ... 2 hits 2 orgs [unclassified Burkholderiales; Burkholderiales Genera incertae sedis]
. . . . Methylibium sp. T29-B ... 1 hits 1 orgs
. . . . Methylibium sp. T29 ... 1 hits 1 orgs
. . . . Gammaproteobacteria ... 12 hits 11 orgs
. . . . . Oceanospirillales ... 4 hits 3 orgs
. . . . . . Alcanivorax pacificus W11-5 ... 1 hits 1 orgs [Alcanivoracaceae; Alcanivorax; Alcanivorax pacificus]
. . . . . . Halomonas ... 3 hits 2 orgs [Halomonadaceae]
. . . . Halomonas elongata DSM 2581 ... 1 hits 1 orgs [Halomonas elongata]
. . . . Halomonas sp. A3H3 ... 2 hits 1 orgs
. . . . . Pseudomonadales ... 6 hits 6 orgs
. . . . . . Acinetobacter baumannii EGD-HP18 ... 1 hits 1 orgs [Moraxellaceae; Acinetobacter; Acinetobacter calcoaceticus/baumannii complex; Acinetobacter baumannii]
. . . . . . Pseudomonas ... 4 hits 4 orgs [Pseudomonadaceae]
. . . . Pseudomonas fluorescens F113 ... 1 hits 1 orgs [Pseudomonas fluorescens group; Pseudomonas fluorescens]
. . . . Pseudomonas aeruginosa ... 2 hits 2 orgs [Pseudomonas aeruginosa group]
. . . . Pseudomonas aeruginosa JD325 ... 1 hits 1 orgs

```
. . . . . . . Pseudomonas sp. EGD-AK9 .........................................     1 hits    1 orgs
. . . . . . Dasania marina DSM 21967 ..........................................     1 hits    1 orgs [unclassified Pseudomonadales; Dasania; Dasania marina]
. . . . . Alkalilimnicola ehrlichii MLHE-1 ....................................     1 hits    1 orgs [Chromatiales; Ectothiorhodospiraceae; Alkalilimnicola;
Alkalilimnicola ehrlichii]
. . . . . Pectobacterium sp. SCC3193 ..........................................     1 hits    1 orgs [Enterobacteriales; Enterobacteriaceae; Pectobacterium]
. . . Spirochaeta sp. JC202 ...................................................     1 hits    1 orgs [Spirochaetes; Spirochaetia; Spirochaetales; Spirochaetaceae;
Spirochaeta]
. . . Cyanobacteria ...........................................................     2 hits    2 orgs
. . . . Mastigocoleus testarum BC008 ..........................................     1 hits    1 orgs [Stigonematales; Mastigocoleus; Mastigocoleus testarum]
. . . . Leptolyngbya boryana PCC 6306 .........................................     1 hits    1 orgs [Oscillatoriophycideae; Oscillatoriales; Leptolyngbya;
Leptolyngbya boryana]
. . Eukaryota .................................................................     2 hits    2 orgs
. . . Pantholops hodgsonii ....................................................     1 hits    1 orgs [Opisthokonta; Metazoa; Eumetazoa; Bilateria; Deuterostomia;
Chordata; Craniata; Vertebrata; Gnathostomata; Teleostomi; Euteleostomi; Sarcopterygii; Dipnotetrapodomorpha; Tetrapoda; Amniota; Mammalia; Theria; Eutheria;
Boreoeutheria; Laurasiatheria; Cetartiodactyla; Ruminantia; Pecora; Bovidae; Antilopinae; Pantholops]
. . . Ricinus communis ........................................................     1 hits    1 orgs [Viridiplantae; Streptophyta; Streptophytina; Embryophyta;
Tracheophyta; Euphyllophyta; Spermatophyta; Magnoliophyta; Mesangiospermae; eudicotyledons; Gunneridae; Pentapetalae; rosids; fabids; Malpighiales; Euphorbiaceae;
Acalyphoideae; Acalypheae; Ricinus]
```
