# Supplementary material for: γ-PGA Hydrolases of Phage Origin in Bacillus subtilis and Other Microbial Genomes
Source: PLoS One. 2015 Jul 9;10(7):e0130810. doi: 10.1371/journal.pone.0130810 (PMC4497714; doi:10.1371/journal.pone.0130810)
Supplement: S2 File — Pfam web page reporting the description (and length) of the DUF867/ PF05908 domain, including the seed alignment used to generate the domain signature. (PDF) [file pone.0130810.s002.pdf]

Family: *DUF867* (PF05908)

3 architectures | 384 sequences | 0 interactions | 199 species | 2 structures

Summary
Domain organisation
Clan
Alignments
HMM logo
Trees
Curation & model
Species
Interactions
Structures

Jump to...
enter ID/acc Go

Summary: Protein of unknown function (DUF867)

Pfam includes annotations and additional family information from a range of different sources. These sources can be accessed via the tabs below.

Wikipedia: Domain of unknown function | Pfam | InterPro

"DUF" families are annotated with the Domain of unknown function Wikipedia article. This is a general article, with no specific information about individual Pfam DUFs. If you have information about this particular DUF, please let us know using the "Add annotation" button below.

Protein of unknown function (DUF867) Provide feedback

This family consists of a number of bacterial and phage proteins with no known function and is present in Bacillus species and the Lambda-like viruses.

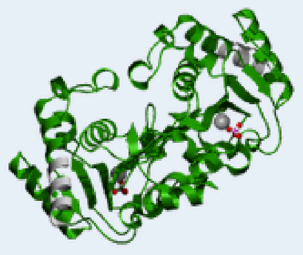

PDB entry 3A9L: Structure of Bacteriophage poly-gamma-glutamate hydrolase

Internal database links

SCOOP:	FleQ

External database links

PANDIT:	PF05908
Pseudofam:	PF05908
SYSTERS:	DUF867

Family: *DUF867* (PF05908)

Curation and family details

This section shows the detailed information about the Pfam family. You can see the definitions of many of the terms in this section in the glossary and a fuller explanation of the scoring system that we use in the scores section of the help pages.

Curation

**Seed source:**	Pfam-B_7778 (release 8.0)
**Previous IDs:**	none
**Type:**	Family
**Author:**	Moxon SJ
**Number in seed:**	37
**Number in full:**	384
**Average length of the domain:**	188.60 aa
**Average identity of full alignment:**	34 %
**Average coverage of the sequence by the domain:**	69.65 %

HMM information

**HMM build commands:**	*build method:* hmmbuild -o /dev/null HMM SEED<br>*search method:* hmmsearch -Z 23193494 -E 1000 --cpu 4 HMM pfamseq
**Model details:**	see table below
**Model length:**	194
**Family (HMM) version:**	6
**Download:**	download the raw HMM for this family

Model details:

Parameter	Sequence	Domain
Gathering cut-off	27.1	27.1
Trusted cut-off	27.6	30.1
Noise cut-off	26.3	26.7

EMBL-EBI

Seed sequence alignment for PF05908

```
Q24P60_DESHY/17-211       YHSFAELSAAEAP.ADYTIKTNDI.G.SDTTVLALHGGGVERGTSELVEALN...GYGKYNTYSFEGLKTAD.NGSLFLR
B8FYB5_DESHD/50-229       LDD..EVNINVKNFGDYEIRTGVT.D.SKVAVIAIHGGKIEQGTSELAYALS...ARNHYNYYTYLGVRDKD.NASLHIP
Q8EL86_OCEIH/31-218       YGSYEELKEHEEQNEDYEINYSQ.RD.SDITVIAIHGGGIEPGTSEVAKGLA...NRMDLSYYLFEGIKPTG.NMILHIE
Q49X79_STAS1/5-199        FMSMSELIDHTEEHKDWQILYNE.RV.AKSLITAVHGGAIEPGTSEIAQLIS...EIGGYSFYTFKGIRKNK.NHELHVT
Q8CPF1_STAES/4-198        FKSMTELKELTKEGKDWEIECEN.RS.SIVTILALHGGGIEPATTELAYTIA...HCGDYNYFSFKGMRSKG.NNELHVT
Q4L5Z4_STAHJ/4-198        YHSMKELQNETIENEDWEIITED.RD.SNVTILAIHGGGIEPATTELARVIA...NDGQFNYFAFNGMRTKG.NNELHVT
Q49US1_STAS1/42-237       YKNFTELKSDTKEHRDWQTNAKTTNN.KDILVTAIHGGGIEPGTSELAKLIS...KKGDYNLYSFEGLMKSN.NQKLHIT
Q8CPX5_STAES/45-240       YADFTELKKDTIKNKDWRIKTKHRKN.KDILVTAIHGGGIEPGTTEIARRIS...NVGKYNFYTFEGLRKSN.NDQLHVT
Q4L4P8_STAHJ/45-240       YSNFEELKENTTEGKDWRIKTKNRKD.NHILVTAIHGGGIEPGTTELARRVA...NIGEYDFYTFEGLMPKH.NERLHIT
Q49UW3_STAS1/43-237       YQSMTDLMDNTNEGTDWELSYND.NN.SDTLIGAIHGGNIEAGTSEAAKLTA...NKGAYRYYAFEGIRPSD.NSDLHVT
Q8CMW8_STAES/242-436      YQSMTQLEKETTEGVDWRKDTKN.TG.NQVLIVAPHGGSIEQGTTELTKALA...DKGNYDYYSFEGIRPKN.NSELHVT
A0QBL0_MYCA1/159-347      PQSLSALLRVTGVTESSQLR.......SGFGFLAIHGGGLEEMTDVIAERAA...AAAGASVYV...VRHPA.AYPHHLP
O05915_MYCTU/159-347      PQSLSELLSQPGVIETSQLR.......SRFGFLAIHGGGLEQVTDLIAERSA...EAAGASVYL...LRHPD.NYPHHLP
A4T704_MYCGI/167-355      PQTLSDLLADPAVTEESVLR.......SRFGFLAIHGGGLERMTDVVAERAA...DAAGASVYV...VRHPD.EYPHHLS
A1ULD7_MYCSK/158-346      PKSLSELLTDPAVTEHSVLR.......SRFGFLAIHGGGLEQMTDVIAERAA...EAADASVYV...VRHPE.QYPHHLP
A0R3T7_MYCS2/154-340      PQSLAELLADPAVRETSTLR.......SRFGFMAIHGGGLEQMTDVIAERAA...AASDASVYV...VRHPD.RYPHHLP
Q852V1_9VIRU/7-199        YPNIEALENAETVGVAYNIEVKRQ.N.PSMIYFSPHAGGIEVGTIELIYRVV...ELTGGSLYLFQGLLPSG.NSRLHVT
Q852V1_9VIRU/7-199 (SS)   SSSHHHHHHHS-TTTSEEEEEEE-.-.SSEEEEEEEEETTSTTHHHHHHHHH...HHH--EEEEEEE--SS-.GGGG---
Q38147_BPSPP/5-190        FDNFFIMESKAELCRDYWFEMRRR.S.RDLVLLAIHGGGIERGTTELLRGMA...AEWNDSYYLFEGR.....NFDYHLT
YOQZ_BACSU/69-258         YSNFEELKENESP.LSYNILTTDV.D.RRVLILAPHGGGIEGGTSELARELS....KSYSAYLFEGLRIPG.ASELHIT
YNDL_BACSU/55-244         YQNFEELKNNEDP.SDYGVVTKET.G.SPVLVLAIHGGGIEGGTSEVARELS....KEYSMYLFEGLKSAG.NSVLHIT
Q65HV4_BACLD/62-251       YSSFEELERNEDP.ASYRITEKNA.R.VPMLIMAIHGGGIEPGTSEIANEVS....KNYSLYLFEGLKSSG.NTDLHIT
A7Z548_BACA2/76-265       YGSFEELKLNEDP.FDFSISTREH.D.PSVLILAIHGGGIEPGTSELASEIA....ENRSLYLFEGLKPAG.NAALHLT
A8FC73_BACP2/8-199        YQSYEELSAHERKGIDYQILHERK.G.DQLLVLSPHAGGIESGVSELIHEIS....SDYSMYLFEGLKVKG.NHVLHIT
Q65HP4_BACLD/41-231       YANFAELAEHETEGEDYQIEYTDM.G.TELLILSPHGGGIEGGVSELVRAFS....ADYSTYLFEGIKPSQ.NWDLHIT
A8FDI2_BACP2/44-235       YRNFKELEENEST.SSYQITANPVPG.SRLLVMSPHGGRIEGGVSEIVHFFD....NDFSTYLFEGLR.EN.ASELHVT
A7Z4Z5_BACA2/36-226       YTDFKDLAEHEDP.DDYHISYNKRKG.SPVLIMSPHGGRIEGGVSEIVRSFR....DDYSTYLFEGLKTHD.NQTLHIT
YMAC_BACSU/36-226         YSNFSSLAENESP.ADYDISYNEKKG.SKVLIMSPHGGRIEGGVSELVRYFN....NEYSTYLFEGLKSHD.NQTLHIT
Q65GP0_BACLD/39-228       YKNFKELQENEK..SGYDIDYHEKAG.SDCLIFSPHGGRIEGGVSELVRAFN....DDYSTYLFEGKKDEN.NSDLHIT
A8FDH9_BACP2/52-238       YESFKQLIRHEK..EGYEIEFHEKGG.SDLLVFSPHGGEIEPGTSEIVEAFQ....QRYSTYLFEGTKQEN.NRDLHIT
Q65L44_BACLD/9-200        YGSFTEMAENEIEGTDYSVAARDA.G.SALLVMAPHGGGIEPGISEIVKAFA....EGASVYLLEGLKRRG.NKSLHVT
A7Z3N6_BACA2/5-194        YDNFAALSAAE...SEYRIIYEEKEG.SKCIVLAPHGGRVEPGVSELVRAFS....DQSSIYLFEGTKRCD.NRSLHLT
YJQB_BACSU/5-194          YDSFAALSASE...SEYRIIYEEKNG.SELIVLGPHGGRIEPGVSELVRAFS....DRCSIYLFEGLKQRN.NRSLHLT
A4F5Y8_SACEN/29-226       YADPALVH.....GTDYRIRHRRHRTPSPPAVIAPHGGEIEAGTSELCLAIATWPDGAAFDYWAFEGLRSPS...ALHVT
A0LGQ9_SYNFM/8-195        FHSYAELKAAEREGTDYRITVRPGK..LPIAVMAPHGGGIEPGTSELARAVA....DGKFTCYCFDGVKPRG.NGSLHLT
Q0A7Q0_ALHEH/4-194        YSNFKALKAEQTEGRDFRVQVAVRDE.AAVAVIAPHGGAIEPGTSELAFAIA....GEQFSFAIFEGTKVAK.NRDLHIT
A3SEN6_9RHOB/5-191        YPDFATLAAAHDQDRDYRITVQDRG..TCVVILAPHGGTIEPETASIARAVA....GNDLSFYLFEALRAGA.HGDYHIT
Q989S0_RHILO/16-205       YMAFADLAAEKTQGVDYDFLVLD.RA.SAVAIVAPHGGWIEHGTSELATAVA...GDD.FSIYLFEGLKPKRPHSELHIP
```

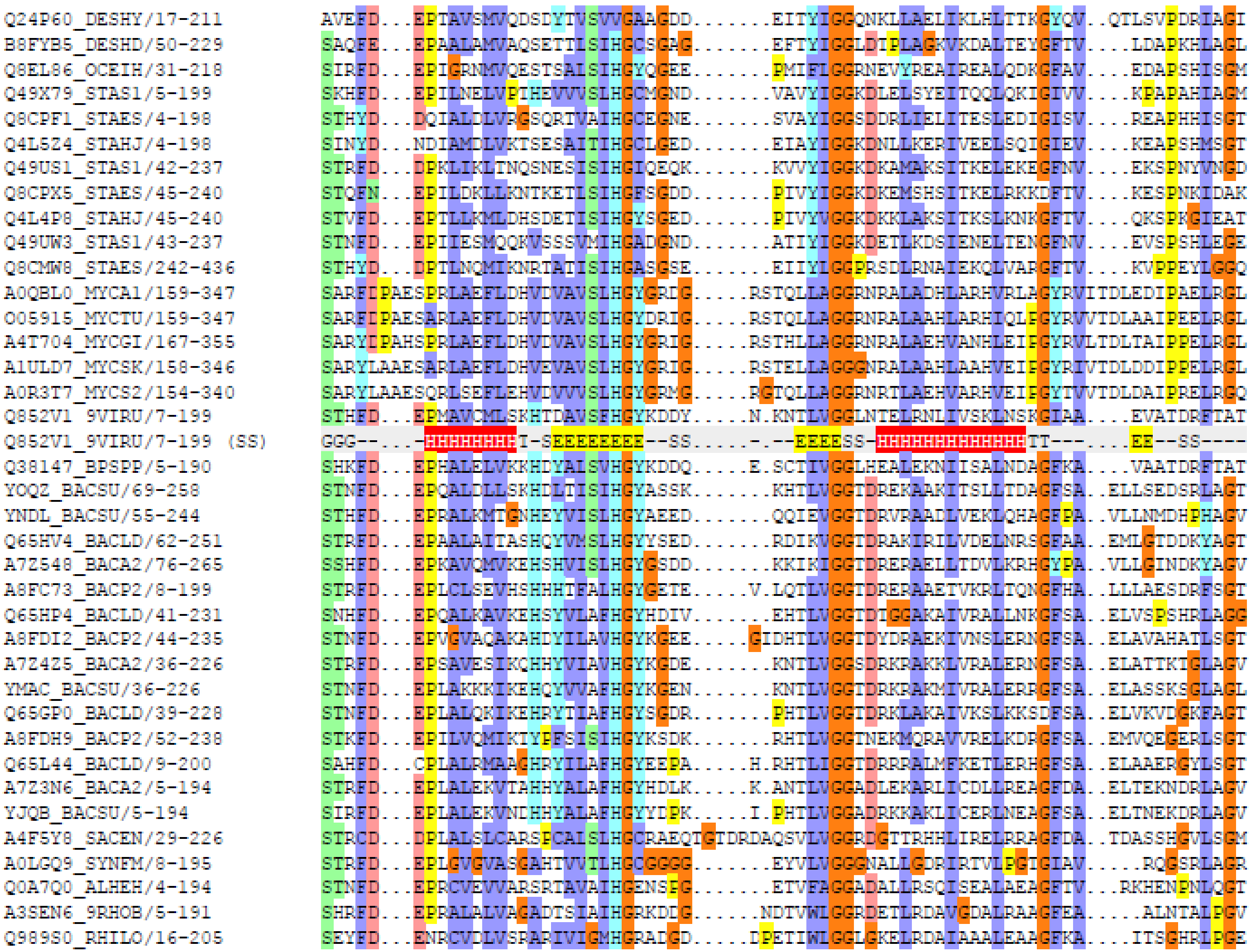
Q24P60_DESHY/17-211
B8FYB5_DESHD/50-229
Q8EL86_OCEIH/31-218
Q49X79_STAS1/5-199
Q8CPF1_STAES/4-198
Q4L5Z4_STAHJ/4-198
Q49US1_STAS1/42-237
Q8CPX5_STAES/45-240
Q4L4P8_STAHJ/45-240
Q49UW3_STAS1/43-237
Q8CMW8_STAES/242-436
A0QBL0_MYCA1/159-347
O05915_MYCTU/159-347
A4T704_MYCGI/167-355
A1ULD7_MYCSK/158-346
A0R3T7_MYCS2/154-340
Q852V1_9VIRU/7-199
Q852V1_9VIRU/7-199 (SS)
Q38147_BPSPP/5-190
YOQZ_BACSU/69-258
YNDL_BACSU/55-244
Q65HV4_BACLD/62-251
A7Z548_BACA2/76-265
A8FC73_BACP2/8-199
Q65HP4_BACLD/41-231
A8FDI2_BACP2/44-235
A7Z4Z5_BACA2/36-226
YMAC_BACSU/36-226
Q65GP0_BACLD/39-228
A8FDH9_BACP2/52-238
Q65L44_BACLD/9-200
A7Z3N6_BACA2/5-194
YJQB_BACSU/5-194
A4F5Y8_SACEN/29-226
A0LGQ9_SYNFM/8-195
Q0A7Q0_ALHEH/4-194
A3SEN6_9RHOB/5-191
Q989S0_RHILO/16-205

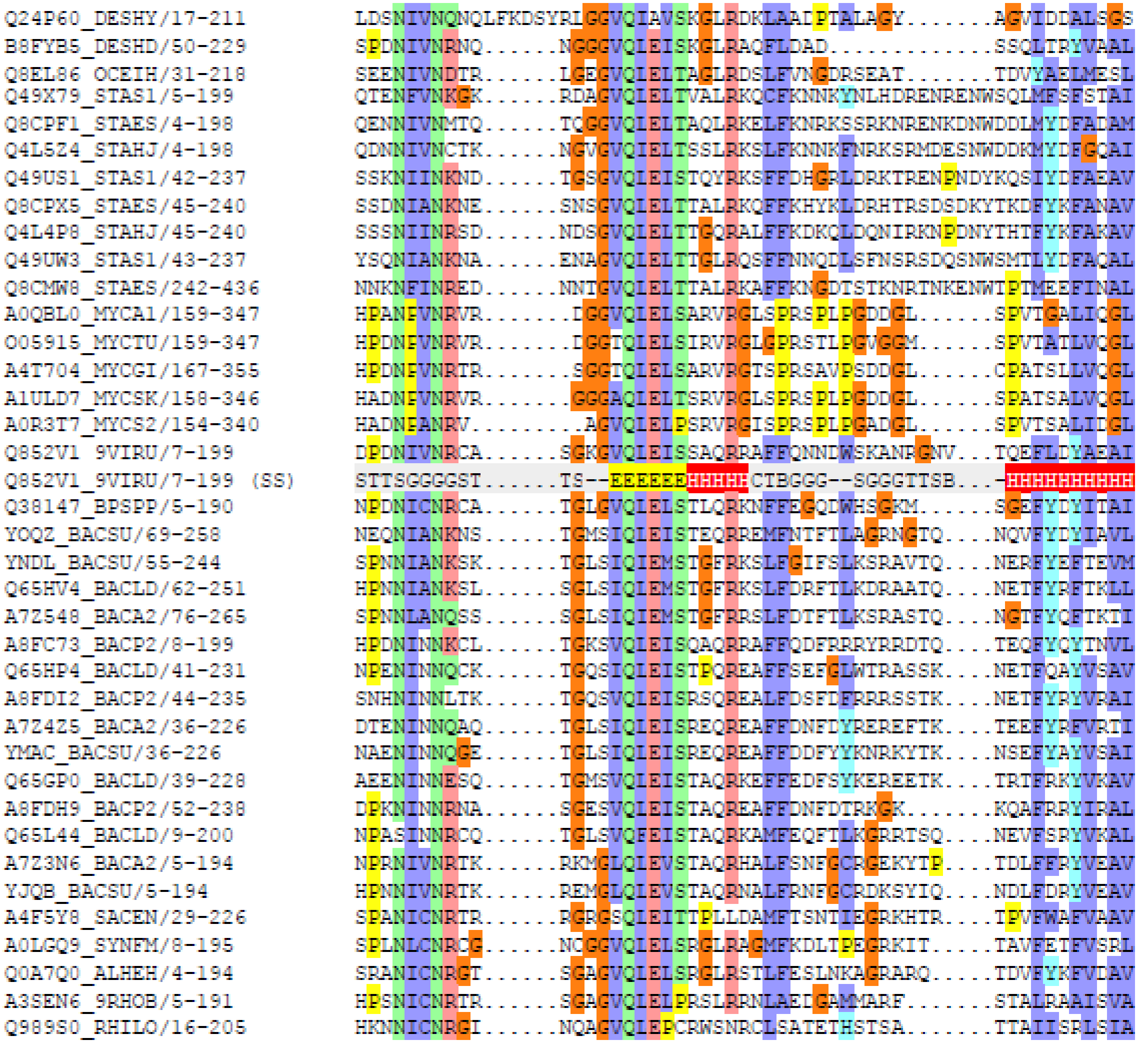

This alignment is coloured according to the ClustalX colouring scheme:

- Glycine (G)
- Proline (P)
- Small or hydrophobic (C,A,V,L,I,M,F,W)
- Hydroxyl or amine amino acids (S,T,N,Q)
- Charged amino-acids (D,E,R,K)
- Histidine or tyrosine (H,Y)

Some UniProt sequences can be mapped to protein structures, in which case we also show the secondary structure definition. These lines are shown below the sequence to which they apply and are marked (SS). The meaning of each of the symbols is as follows:

C Random coil
H Alpha-helix
G 3(10) helix
I Pi-helix
E Hydrogen bonded beta-strand (extended strand)
B Residue in isolated beta-bridge
T H-bonded turn (3-turn, 4-turn, or 5-turn)
S Bend (five-residue bend centered at residue i)
